# Characterization of Composition and Structure–Property Relationships of Commercial Post-Consumer Polyethylene and Polypropylene Recyclates

**DOI:** 10.3390/polym13101574

**Published:** 2021-05-14

**Authors:** Markus Gall, Paul J. Freudenthaler, Joerg Fischer, Reinhold W. Lang

**Affiliations:** Institute of Polymeric Materials and Testing, Johannes Kepler University Linz, Altenberger Straße 69, 4040 Linz, Austria; paul.freudenthaler@jku.at (P.J.F.); joerg.fischer@jku.at (J.F.); reinhold.lang@jku.at (R.W.L.)

**Keywords:** circular economy, composition, contamination, mechanical recycling, polyethylene, polyolefin, polypropylene, post-consumer, quality, structure–property relationship

## Abstract

The current efforts in moving closer towards a circular plastics economy puts massive pressure on recycled plastics, especially recycled polyethylene (rPE) and recycled polypropylene (rPP) to enter new markets. Their market penetration remained low so far, despite PE and PP constituting the largest share of plastic wastes. However, with the current imperative of more circularity comes a new focus on performance of recyclates. Hence, a detailed understanding of composition and structure–property relationships of post-consumer recyclates has to be developed. Five recycling companies from the Austrian and German markets were asked to supply their purest high-quality rPE and rPP grades. These were characterized by differential scanning calorimetry (DSC), thermo-gravimetric analysis (TGA), and Fourier-transform infrared (FTIR) spectroscopy, and micro-imaging. Technological characterization included density measurements, determination of the melt flow rate (MFR), and Charpy impact testing. All recyclates contained diverse contaminants and inclusions ranging from legacy fillers like calcium carbonate to polymeric contaminants like polyamides or polyolefin cross-contamination. The overall amount, size, and distribution of contaminants varied significantly among suppliers. Furthermore, first structure–property relationships for polyolefin recyclates that link inorganic content and polymeric purity with density and impact performance could be derived.

## 1. Introduction

Currently, significant efforts are undertaken to redesign the plastics industry in pursuit of the vision of a circular plastics economy. In the European Union (EU), it is the declared goal of the European Commission to boost the utilization of recycled plastics for the manufacture of new products to a level of 10 million tons per year in 2025 [1] up from about 4 million tons in 2018 [2]. This effort is backed up by new recycling targets for plastics in the EU member states including recycling of 55% of all plastic packaging waste by 2030 [3] and a minimum recycled content target of 30% in all beverage bottles by 2029 [4]. Furthermore, the commission has announced to put forward additional goals for minimum recycled content in applications yet to be defined [5].

The polyolefins (PO), such as polyethylene (PE) and polypropylene (PP), make up about half of all plastics processed in the EU [6]. Due to their dominance in short lived products and packaging [6] they constitute the largest share of all available post-consumer plastic wastes [2,7], making up more than half of the 29 million tons of plastic wastes collected annually in the EU. Very contrary to the waste arising, the mechanical recycling of PO materials is rather underdeveloped in the EU. It has recently been estimated that the output of European recycling operations is satisfying not more than 8% and 3% of the European plastics converter demand for PE-HD and PP, respectively [8], based on data from 2018.

With a view on these numbers, it is obvious that materials based on recycled PE (rPE) and recycled PP (rPP) have an outstanding potential of contributing a significant share to the Commission’s 10 megaton target [1]. In order to do so, however, rPE and rPP will have to enter new markets with more demanding and more critical applications. Many of these applications and markets demand strict adherence to tight material specifications [9,10,11,12]. They may even require compliance with regulatory and legislative frameworks such as in food-contact applications [13,14]. Generally speaking, many of these applications will pose more diverse, more complex, and more challenging performance requirements compared to what has been the standard in mechanical PO recycling until now [12]. Still, the highest shares of recyclates are used in lower quality applications in the agricultural and building sectors [2,6].

For virgin PO materials, tailor-made property profiles and high reliability of in-service performance have become a reality due to a vast body of research and development in the field of material–structure–processing–property–performance relationships [15,16,17,18]. Until now, such an understanding is greatly lacking for recycled polyolefins, particularly from post-consumer waste streams. An overview of typical properties of virgin PE and PP is provided in Table 1.

It is often argued that degradation of the molecular structure of both PE and PP is the main issue when it comes to mechanical recycling [21]. As a matter of facts, there is a considerable number of publications on the effects of reprocessing and “simulated recycling” of PE and PP [22,23,24,25,26,27,28]. While this work is definitely an essential pillar for our understanding of recycled PO materials, the picture is far from complete. Most of these works have actually not investigated real recyclates obtained from recycling operations, but virgin PE and PP. Vilaplana & Karlsson [29] were among the first to develop a more comprehensive view on the quality of recycled polyolefins. Besides degradation and aging, they explicitly took compositional aspects of recycled materials such as the presence of low-molecular weight compounds and the degree of mixing (of different polymers) into consideration [29].

In the meanwhile, the discussion of quality of recyclates has gained momentum [30,31] and more and more effort is being put into investigations of different plastic waste fractions, their composition, and the associated implications for recycling [32,33,34,35,36]. It is increasingly being understood that there is an intrinsic connection between recyclate composition, including minor fractions and contaminants, and the resulting structure–property relationships [37,38]. Recent developments, such as the design-from-recycling concept [39,40,41,42], try to account for these peculiarities of (post-consumer) recyclates. However, it has to be admitted that the classical situation when it comes to using rPE and rPP materials is still such that we have only very rudimentary information regarding their composition and we are still far from being in the position of tailoring structure–property profiles of PO recyclates for challenging applications.

It is hence of paramount importance to shed light onto the actual composition of real commercial PO recyclates that reflect the current status quo in the market. Elucidating their composition and investigating their fundamental structure–property relationships that govern their technical utility are key for more widespread use of recyclates. The present work is a first attempt in this regard.

## 2. Materials and Methods

Five plastic recycling companies, one located in Austria and four located in Germany, supplied their purest high-quality PE and PP recyclates to be included in this work. Confidentiality was agreed as a prerequisite for participation in this multi-firm survey. Nevertheless, the participating companies, the names of their analyzed products and corresponding data sheets are known to the authors of this article. The materials received for analysis are recyclates derived from a variety of commercially available post-consumer plastic waste fractions using the proprietary treatment, recycling, and converting technologies of the individual companies.

The sampling strategy focused on low-melt-flow-rate grades of high-density polyethylene (PE-HD) suitable for extrusion and blow-molding purposes and more easy-flowing polypropylene (PP) grades suitable for injection molding applications. The sample code convention used is *rPE-X* and *rPP-X* for PE-HD and PP recyclates, respectively, where X represents the individual supplying company denoted with A, C, D, E, or F. In total, six PE-HD recyclates (rPE-A, rPE-C, rPE-D, rPE-E1, rPE-E2, and rPE-F) and four PP recyclates (rPP-A, rPP-C, rPP-D, and rPP-F) were analyzed. Company E supplied two different grades of PE-HD recyclates (rPE-E1 and rPE-E2) and no PP recyclate. An overview of all sample materials including relevant data sheet information is provided in Table 2.

All materials were received in the form of granules. Multi-purpose specimens of Type A according to ISO 3167 [43] were produced by injection molding using a hydraulic Victory 60 (Engel, Schwertberg, Austria) injection molding machine. The injection parameters were chosen in accordance with the polymer-specific material standard to fit the required conditions for PE and PP, respectively. Hence, rPE materials were processed according to the injection parameters outlined in ISO 17855-2:2016 [44], including a melt temperature of 210 °C, an average injection velocity of 100 mm/s, a mold temperature of 40 °C, a cooling time of 35 s, and a total cycle time of 40 s. Likewise, rPP materials were processed according to ISO 1873-2:2007 [45] that demands a melt temperature of 200 °C, an average injection velocity of 200 mm/s, a mold temperature of 40 °C, a holding pressure time of 40 s, and a total cycle time of 60 s for PP materials with a melt flow rate value greater than 7 g/10 min.

The set of methods employed for compositional recyclate analysis included Fourier-transform infrared (FTIR) spectroscopy in the attenuated total reflection (ATR) mode, differential scanning calorimetry (DSC), thermo-gravimetric analysis (TGA), density measurements, melt flow rate (MFR) measurements, optical microscopy, and spatially resolved ATR-FTIR imaging (scanning microscopy).

ATR-FTIR spectra were recorded from the surfaces of multi-purpose specimens. A spectrum 100 FTIR analyzer (PerkinElmer, Waltham, MA, USA) equipped with an ATR unit with a diamond/ZnSe crystal was used for this purpose. For each material, spectra were collected from arbitrary surface locations of three individual multi-purpose specimens. Each measurement included four scans of the wavenumber range from 4000 cm^−1^ to 650 cm^−1^ with a spectral resolution of 4 cm^−1^. A contact force of about 100 N was used.

A DSC 8000 power compensation differential scanning calorimeter (PerkinElmer, Waltham, MA, USA) was used for thermal analysis. Samples of 4 ± 1 mg were cut from injection molded multi-purpose specimens and put into aluminum pans with holes and a lid. The temperature program included an initial isotherm of 1 min at 0 °C, a first heating scan from 0 °C to 200 °C, a cooling scan from 200 °C down to 0 °C, and a second heating scan from 0 °C to 200 °C. The heating and cooling rates were set to 10 K/min and nitrogen was used as a purge gas with a flow rate of 20 mL/min. Triplets were done for each recyclate grade and an empty pan was used for baseline correction. The melting enthalpy ∆H_m_ observed during the second heating scan was evaluated in the temperature ranges from 60 °C to 136 °C for the PE fraction and from 136 °C to 170 °C for the PP fraction of the recyclates, respectively. Temperature intervals from 125 °C to 60 °C and from 135 °C to 100 °C were used for evaluating crystallization enthalpy ∆H_c_ of PE and PP fractions, respectively.

The TGA was performed in an STA 6000 simultaneous thermal analyzer (PerkinElmer, Waltham, MA, USA) using nitrogen as a purge gas (20 mL/min). Samples of 19 ± 2 mg were cut from multi-purpose specimens and put into open ceramic crucibles. The temperature program included an initial isotherm at 30 °C for 1 min, a heating step from 30 °C to 850 °C with a heating rate of 20 K/min, a second isotherm of 10 min at 850 °C during which the purge gas was changed from nitrogen to oxygen (at 20 mL/min), followed by a last isotherm of 1 min in nitrogen. Duplicates were done for each recyclate grade.

Density measurements were performed according to ISO 1183-1:2019 [46] method A (immersion) using a Sartorius CPA 225D lab balance with a buoyancy setup and de-ionized water with a temperature of 23 °C. Samples were cut from multi-purpose specimen sprue-sided shoulders. Per material, five samples were used from individual multi-purpose specimen for the calculation of average values and standard deviations. In the first step, the respective sample was weighed dry, measuring its mass in air *m_S,A_*. In the second step, the sample was immersed in deionized water and put below a buoyancy cage which was connected to the scale, enabling the measurement of the sample buoyancy in grams *m_S.IL_* without the need of a sinker. A wire was used to remove air bubbles. The temperature of the immersion liquid was recorded for the calculation of its density *ρ_IL_*. The sample density *ρ_S_* was calculated according to the following formula using the apparatus-specific correction variables *A* and *B*

(1) $$\rho_{s}=\frac{m_{S.A}*\rho_{IL}}{A*\left(m_{S.A}-m_{S.IL}\right)}+B$$

where *A* = 0.99983 and *B* = 0.0012.

The MFR was tested with an Mflow indexer (ZwickRoell, Ulm, GER) using granules (as delivered), a testing mass of 2.16 kg and temperatures of 190 °C and 230 °C for PE and PP materials, respectively, in accordance with ISO 1133 [47].

Charpy notched impact strength (NIS) measurements were used as a technological performance indicator that is sensitive towards the presence of defects. The tests were conducted according to ISO 179-1/1eA [48], using ten Type 1 specimens with a V-shaped notch and edgewise impact direction per recyclate. The specimens were taken from the center section of injection molded Type A multi-purpose specimens according to ISO 3167. Impactors of 5 J and 0.5 J were used for rPE and rPP materials, respectively. The tests were carried out at a temperature of 23 °C and a relative humidity of 50% and the specimens were stored under these conditions for at least 96 h prior to testing.

For the microscopic investigations by means of optical microscopy and ATR-FTIR imaging, pieces were cut out from the central part of multi-purpose specimens and fixed in transparent embedding resin. These samples were then polished in an automatic polishing unit using water and superfine sandpapers with grit up to 4000 to reveal the specimen cross-section and provide a flat surface for ATR-FTIR imaging.

An SZX16 stereo microscope (Olympus, Tokyo, Japan) was used to record optical images of the polished specimen cross-sections. A spotlight 400 imaging system (PerkinElmer) equipped with a germanium ATR-crystal was used together with a spectrum 100 FTIR spectrometer (PerkinElmer, Waltham, MA, USA) to record spatially-resolved FTIR-images of the polished specimen cross-sections in ATR-mode. Two locations within each cross-section were scanned, one in the center and another one where an inclusion was found by prior visual inspection. Where no inclusions were visually detectable, an arbitrarily chosen location was taken. The image area scanned was between 150 × 150 µm^2^ and 300 × 300 µm^2^, depending on the size of suspected inclusions. All images were scanned twice with a spatial resolution of 1.56 µm and a spectral resolution of 16 cm^−1^ in the wavenumber range from 4000 cm^−1^ to 720 cm^−1^.

## 3. Results

### 3.1. Compositional Analysis

#### 3.1.1. FTIR Spectroscopic Characterization and Imaging

**Figure 1:**
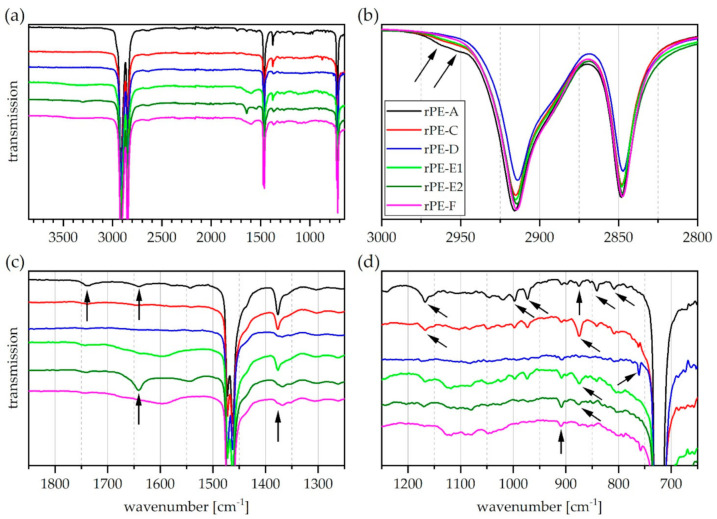
Graphical illustration of ATR-FTIR transmission spectra of rPE materials. (**a**) Entire wavenumber range investigated (**b**) magnification of CH_2_ and CH_3_ stretching vibration region (**c**) magnification of carbonyl and amide region and (**d**) magnification of bending and skeletal vibration region. Only one representative curve is shown per grade. Curves are stacked to improve visibility. Arrows indicate absorption bands of interest.

The ATR-FTIR spectra of all rPE materials are depicted in Figure 1. The spectra are presented in a stacked arrangement resulting from arbitrary shifts along the vertical axis to increase visibility. While Figure 1a provides a view of the entire wavenumber range investigated, Figure 1b–d are magnifications of specific regions of interest.

Some differences between the individual rPE spectra are already discernable in Figure 1a. A broad and quite flat peak in the wavenumber range between 3500 cm^−1^ and 3100 cm^−1^ is visible in the spectra of rPE-E1 and F together with another broad band at around 1600 cm^−1^. The latter is better observed in Figure 1c. This pattern is characteristic for hydroxyl groups encountered in diverse impurities and specifically for associated hydrogen bonds of surface bound water [49] whichmight be present due to hygroscopic additives or polar contaminants on the sample surface [42]. The spectrum of rPE-E2 contains a weak band centered around 3300 cm^−1^, which was not detected in the other rPE spectra. This band might originate from N-H stretching vibrations found in amides [49,50,51].

In Figure 1b, the spectra of rPE-A, C, and E1 exhibit a shoulder at above 2950 cm^−1^, which is not commonly present in pure PE. Closer inspection of Figure 1c reveals that this shoulder appears in conjunction with a pronounced peak at 1377 cm^−1^. This peak is absent in rPE-D, E2, and F (see black arrow). Both bands are assigned to different vibration modes of the CH_3_-group and likely indicate the presence of PP [50,51]. The band located at 1377 cm^−1^ can also be used as a marker to discern branched PE types such as PE-LD and PE-LLD from PE-HD [51,52]. Typical PE-HD would exhibit the highest peak between 1400 and 1300 cm^−1^ at about 1368 cm^−1^, without an additional band at 1377 cm^−1^ [52]. In the present case, the spectra of rPE-D, E2, and F have a local maximum at 1368 cm^−1^. However, it is accompanied by a shoulder at 1377 cm^−1^ as observed in PE-LLD materials. Whether this now indicates the presence of PE-LLD or PP or both, cannot be assessed with certainty based on these data.

Another feature in Figure 1c is a band of moderate intensity at around 1740 cm^−1^ in the spectrum of rPE-A. This is the characteristic signature of a carbonyl group found in esters, ketones, and aldehydes [49]. This band could be detected in the spectra of all other rPE materials as well, though with very low intensities. Carbonyl functionalities in polyolefins can be a sign of chemical aging or degradation of the polymer backbone [49,53] due to thermo-oxidative stress or UV-light exposure. Besides that, a number of common additives such as stearates are another possible origin for such bands [54]. Carbonyl-containing polymers such as polyesters should be considered in this context as well. However, no specific spectral signatures that would corroborate the presence of, e.g., polyethylene terephthalate (PET) were detected in ATR-FTIR surface spectra of the recyclates.

Furthermore, there is a strong peak at 1640 cm^−1^ (Figure 1c) in the spectrum of rPE-E2, which appears together with a weaker band at 1540 cm^−1^. The same pattern, though much weaker and without the band at 3300 cm^−1^, is found in rPE-A. These bands can be assigned to amide functionalities that might stem from polyamide (PA) [49] or amide-based additives such as slip agents [54] that are sometimes found in recyclates [11,55].

The low wavenumber region is depicted in Figure 1d. Further absorption bands that are typical for PP [49,50] can be found in this region, specifically in the spectra of rPE-A, C, and E1. These bands are located at 1167, 997, 973, 841, and 808 cm^−1^ [49,50] and they are of low intensity. Moreover, all rPE spectra exhibit a very weak band located at 909 cm^−1^, which is indicative of vinyl groups [49,53]. Such an unsaturated group is rather common in PE-LD and PE-LLD [51], whereas in PE-HD it can be interpreted as a sign of degradation [47]. Notably, a band at 890 cm^−1^, which in conjunction with the 909 cm^−1^ band can be used to differentiate PE-LD from PE-LLD [51,52], was not found in any spectrum.

A band of low intensity at 875 cm^−1^ is present in the spectra of rPE-A, C, E1, and E2. It is strongest in rPE-C and E1 and can be attributed to the presence of calcium carbonate (CaCO_3_) [49,54,56], a typical filler for polyolefins. A weak but sharp band at 760 cm^−1^ can be observed in the spectra of rPE-D and rPE-F. This band might originate from the presence of a mono-substituted aromatic ring such as in polystyrene [49]. However, many different additives and pigments contain such a group too [54].

**Figure 2:**
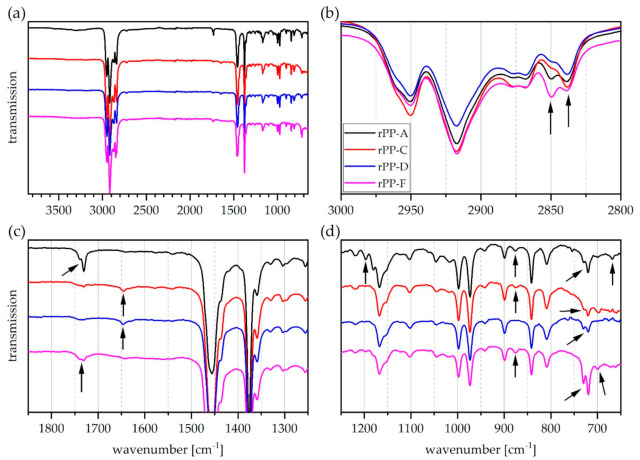
Graphical illustration of ATR-FTIR transmission spectra of rPP materials. (**a**) Entire wavenumber range investigated, (**b**) magnification of CH_2_ and CH_3_ stretching vibration region, (**c**) magnification of carbonyl and amide region, and (**d**) magnification of bending and skeletal vibration region. Only one representative curve is shown per grade. Curves are stacked to improve visibility. Arrows indicate absorption bands of interest.

The ATR-FTIR spectra of all rPP materials are depicted in Figure 2. Overall, the situation is comparable to that of the rPE materials described above. The rPP spectra clearly resemble typical virgin PP spectra (Figure 2a). However, again, a number of differences and peculiarities can be observed, some of which are similar or even equivalent to those observed in the rPE spectra in Figure 1.

Similarly to some rPEs, a broad and flat peak between 3600 cm^−1^ and 3100 cm^−1^ was observed in the rPP-A spectrum and to some extent in rPP-F. In the case of rPP-A, this flat peak carries a number of superimposed individual peaks in it. The respective maxima are located at 3540, 3398, 3300, and 3224 cm^−1^ (not shown). An unambiguous assignment seems unlikely, but the band at 3300 cm^−1^ may stem from N-H stretching vibrations typical for polyamides [50] and the occurrence of peaks at 3398 cm^−1^ and 3200 cm^−1^ has been associated with surface-bound fatty amide slip agents [11,55]. As mentioned above, broad IR bands above 3000 cm^−1^ may also originate from hydroxyl groups and hydrogen bonds in impurities or surface bound water.

The magnification of the C-H stretching vibration region in Figure 2b reveals interesting differences in relative intensities of the neighboring peaks at 2850 cm^−1^ and 2838 cm^−1^ (see black arrows). The former is stronger than the latter in the rPP-F spectrum, whereas the opposite is the case for rPP-C and D. Both bands are attributed to the symmetric C-H stretching vibration of a CH_2_-group, but the one at 2850 cm^−1^ is found in PE while the band at 2838 cm^−1^ occurs in PP [50]. This indicates that rPP-F might contain some PE.

A carbonyl peak is present in all rPP spectra (see Figure 2c), but it is particularly strong in rPP-A and F. In these two instances it appears as a twin peak with the major absorption at 1730 cm^−1^ and a minor peak or a shoulder at 1740 cm^−1^. As indicated above, a carbonyl functionality in polyolefins may arise from thermo-oxidative or photochemical degradation as well as from the presence of additives based on esters.

In Figure 2d, a twin peak at 730/720 cm^−1^ is clearly visible in all rPP spectra, but their relative intensity differs among grades. It is strongest in rPP-A and F, but less pronounced in rPP-C and D. This pattern is typically found in PE materials [49,50,56], but should be absent in PP homopolymers. It may appear in PP copolymers [53], but the intensity is typically very low due to low molar concentration of ethylene units. The detection of this twin peak is hence attributed to polyolefin cross-contamination by PE fractions within the rPP materials and it is in agreement with the pattern at 2850/2838 cm^−1^ described above.

Regarding bands of very low intensity in Figure 2d, it can be said that there is peak at 875 cm^−1^ (indicating calcium carbonate) in all rPP spectra except rPP-D. Furthermore, the same rPP grades exhibit a very weak single band at 700 cm^−1^. This band might stem from contamination with polystyrene [50,56]. A band at 670 cm^−1^ was detected in the rPP-A spectrum and it seems to appear together with two more bands of low intensity at 1018 cm^−1^ and 3676 cm^−1^. This pattern can be assigned to talc [49], another common inorganic filler in polyolefin materials. The spectrum of rPP-A contains additional bands at 1197 cm^−1^ and 1180 cm^−1^ (see black arrow) that were not found in such clearly visible manner in any other recyclate. These bands could not be reasonably assigned.

**Figure 3:**
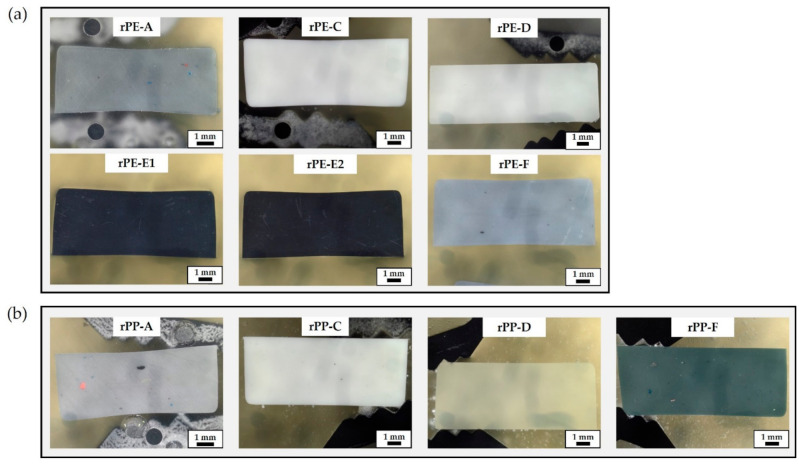
Collection of optical microscopy images of (**a**) rPE materials and (**b**) rPP materials. Images show polished cross-sections of injection molded multi-purpose specimens embedded in resin.

A collection of microscopic images showing polished cross-sections of injection molded specimens of each recyclate grade is provided in Figure 3. The rPE materials are shown in Figure 3a and the rPP materials in Figure 3b. Each cross-section is approximately 4 mm × 10 mm in size. It is evident that the recyclates differ in color. The materials rPE-A, rPE-F, and rPP-A are grey and they contain discrete inclusions of diverse colors, shapes, and sizes. The materials rPE-C, rPE-D, and rPP-C are white and some dark spots can be identified in the rPP-C cross-section only. The material rPP-D is best referred to as natural, perhaps with a yellowish shading but a homogeneous appearance, while rPP-F contains numerous inclusions and its overall appearance is a mix of dark colors. The materials rPE-E1 and E2 are the only black recyclates in this work. No inclusions are visually detectable in these grades at the magnification chosen for Figure 3.

In the phase of sourcing the samples for this work from different recycling companies, all suppliers were asked to provide their high-quality grades with highest purity (notably from post-consumer, not post-industrial waste streams). Undeniably, there is a quite wide range of different purity levels in the market, at least in terms of visual purity and quality.

An ATR-FTIR imaging technique was used to further characterize and potentially identify some of the inclusions present in the sample cross-sections. Selected images of each recyclate grade are compiled in Figure 4, where the rPEs are found in Figure 4a and the rPPs are found in Figure 4b. Where a clear identification was possible based on band assignments using the references [49,50,51], the respective inclusion is labeled. A question mark indicates inclusions that could not be identified. The corresponding ATR-FTIR spectra are provided in the supplementary information together with additional ATR-FTIR images and spectra for each recyclate material (see Figures S1 to S20).

Rather big particles of polyamide (PA) were detected in rPE-C and E2 as well as in rPP-A and D. The bands used for assignment were 3280, 1640, and 1536 cm^−1^. Exemplary spectra are spectrum “4” in Figure S3, spectrum “5” in Figure S9, spectrum “5” in Figure S13, and spectrum “4” in Figure S17. Significant PE inclusions were found in rPP-A, C, and F. Sample spectra can be observed in Figure S13 (“4”), Figure S14 (“4”), Figure S19 (“3”), and Figure S20 (“4”). Interestingly, no big inclusions of PP could be clearly detected in the rPE materials, despite characteristic PP bands in the ATR-FTIR spectra of rPE-A, C, and E1 depicted in Figure 1d. Ethylene-vinyl-acetate copolymer (EVA) was detected in rPE-E1 and F. The bands used for assignment were 1736, 1240, and 1016 cm^−1^. Sample spectra can be observed in Figure S7 (“4”) and Figure S11 (“5”). Besides foreign polymers, inorganic inclusions were found too. Mainly calcium carbonate was detected, especially in rPE-C, E1, and F as well as in rPP-A, C, D, and F. The bands used for assignment were 1792 cm^−1^ and 872 cm^−1^ together with a broad band at around 1400 cm^−1^. These bands always occur as additional or superimposed bands in the respective polymer spectra. Examples can be seen in Figure S4 (“3”), Figure S6 (“4”), Figure S11 (“4”), Figure S13 (“3”), and Figure S18 (“4”). Due to the occasional occurrence of broad and intense peaks centered around 1000 cm^−1^, we assume the presence of talcum too.

However, unambiguous identification of inclusions and disperse phases was difficult or even impossible in many cases. The rather coarse spectral resolution in the ATR-FTIR imaging mode and the apparent mixing of different components often resulted in relatively broad and unspecific bands that likely consisted of several superimposed peaks. The fact that many spectra could not be clearly assigned suggests that the chemical complexity of the recyclates analyzed in this work is in fact very high. The range of minor components present in each recyclate definitely extends way beyond polyolefins (PE, PP), polyamides, and calcium carbonate.

Furthermore, the range of particle shapes and sizes seems to be wide too. Many particles are in the lower micrometer range, where obtaining an unbiased FTIR spectrum is already challenging. Other inclusions exceeded 100 µm in size and some are visible to the bare eye (see for example rPP-A in Figure 3b).

#### 3.1.2. Thermo-Analytical Characterization

**Figure 5:**
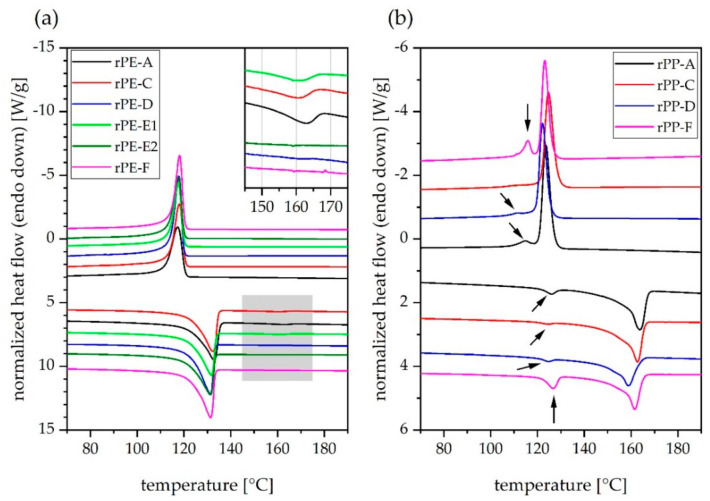
DSC curves of (**a**) rPE and (**b**) rPP materials, respectively. Upper curves are obtained from cooling scans, lower curves from second heating scans. Image (**a**) contains a magnification of the PP melting temperature range (grey area) in the upper right corner. Curves have been stacked and shifted arbitrarily along vertical axis to enhance comparability. Endothermal direction is down.

In the DSC thermograms depicted in Figure 5, most recyclates revealed two distinct melting peaks in the second heating scan (as well as in the first one, which is not shown). Furthermore, two separate crystallization peaks in the corresponding cooling curves could be observed. This was the case for three out of six rPE materials and all four rPP materials investigated (see black arrows in Figure 5b). Relevant data derived from DSC analysis including peak temperatures and enthalpy values for melting and crystallization events are compiled in Table 3.

The peak temperatures of the major melting events in the DSC curves of the rPE materials are all between 131 °C and 132 °C, whereas those of the minor melting peaks ranged from 159 °C to 162 °C. The former is in good agreement with typical melting peak temperatures of PE-HD [20], while the latter is attributed to the melting of a PP fraction [16,20]. For the rPP materials, the peak temperatures of the major melting peaks were in the range of 159 °C to 164 °C, whereas the minor melting events have peak temperatures between 124 °C and 127 °C. In this case, the former corresponds well with PP [16,20], while the latter fits to both PE-HD and PE-LLD. Due to the fact that PE-LLD is mainly used in film applications [19], it can be assumed that the present PE phase is likely PE-HD, and the comparatively moderate melting peak temperatures are a consequence of the low volume fraction and the mixing with PP as reported in the literature [57]. A definite answer to whether PE-HD or PE-LLD (or a mix) is present, requires the use of more sensitive characterization techniques, such as temperature rising elution fractionation (TREF).

The melting enthalpies of the PE fractions of the rPE materials range from 173 J/g to 201 J/g. In a pure PE-HD material these values would correspond to a degree of crystallinity between 59% and 69% [58]. The melting enthalpy values of the PP fractions of the rPP materials range from 57 J/g to 74 J/g. This would correspond to a degree of crystallinity between 28% and 36% in a pure PP material [58]. However, the actual degrees of crystallinity of both the PE and the PP fractions within the respective recyclates are higher than that, because the enthalpy values stated herein are expressed in Joules per gram of recyclate. Since the mass of the pure PE fraction within the rPE materials is unknown, the enthalpy measured in the PE melting temperature range is divided by the total sample mass, including any potentially present non-PE mass fractions such as those of foreign polymers or inorganic substances. The same applies to the rPP materials investigated herein.

With regards to melting enthalpies, the level of polyolefin cross-contamination by PP fractions within the rPE materials seems to be low. Average melting enthalpy values of 4.0, 3.7, and2.6 J/g were detected for rPE-A, C, and E1, respectively, and no such determination was possible for rPE-D, E2, and F. This is in good agreement with the respective ATR-FTIR spectra of rPE-A, C, and E1 (see the shoulder at 2950 cm^−1^ in Figure 1b, the peak at 1377 cm^−1^ in Figure 1c, as well as bands at 1167 cm^−1^, 997 cm^−1^, 973 cm^−1^, and 841 cm^−1^ in Figure 1d). Contrary to that, the minor melting peaks detected in all rPP materials differ significantly in size (see Figure 5b). While rPP-C exhibited the lowest enthalpy value of only 1 J/g, rPP-F reached about 12 J/g in the PP melting range. The level of polyolefin cross-contamination by a PE-HD fraction within an rPP material is hence the lowest in rPP-C and highest in rPP-F. This is in good agreement with ATR-FTIR results discussed above (see shape of the twin peak at 2850/2838 cm^−1^ in Figure 2b and relative size of the twin peak at 730/720 cm^−1^ in Figure 2d).

**Figure 6:**
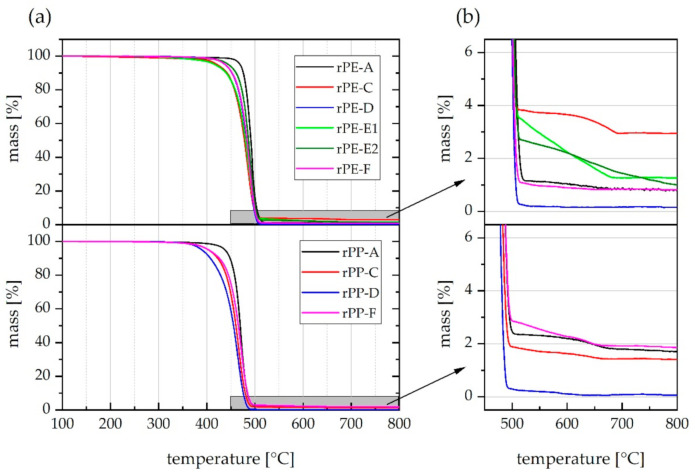
Pyrolysis curves obtained from TGA. (**a**) Entire temperature range, (**b**) magnification of the second decomposition step after polymer backbone degradation. Curves of rPE materials are shown in the upper half of (**a**,**b**), while curves of rPP materials are shown in the lower half. One representative curve is shown per material grade.

The curves obtained from TGA are depicted in Figure 6. One representative curve is shown per recyclate grade. It can be observed that the main decomposition step of rPP materials in Figure 6a occurs at slightly lower temperatures, than that of rPE materials. This is due to the fact that PP is thermally less stable than PE [59]. However, no sample exhibited pronounced loss of mass at temperatures below 300 °C. Such premature loss of mass could indicate the presence of humidity, organic volatiles, or foreign polymers that tend to cleave certain chemical groups before decomposition of the polymer backbone, such as the release of H-Cl from polyvinylchloride (PVC) [59].

Differences among the recyclates become evident in Figure 6b that illustrates the higher temperature range, after completion of polymer backbone decomposition. Only the pyrolysis curves of rPE-D and rPP-D drop down to a level close to zero as it is expected for unfilled polyolefins [59]. In the case of rPE-C and rPP-A, a pronounced second decomposition step can be observed at around 650 °C, while rPE-E1 and rPE-E2 show a more linear decline in residual mass with further increasing temperature (though with obviously different slopes). The step-like loss of mass at around 650 °C can be linked to the cleavage of CO_2_ from CaCO_3_. As a matter of facts, the extent of this change in mass can be used to estimate the initial content of calcium carbonate due to a simple stoichiometric relation based on the molecular masses of CaCO_3_ and CO_2_ according to Equation (2) [59]

(2) $$m_{CaCO3}=\frac{∆m}{0.44}$$

where *m_CaCO3_* is the content of calcium carbonate in wt.% and ∆*m* is the change of mass before and after suspected CO_2_ release, e.g., between 520 °C and 800 °C.

**Figure 7:**
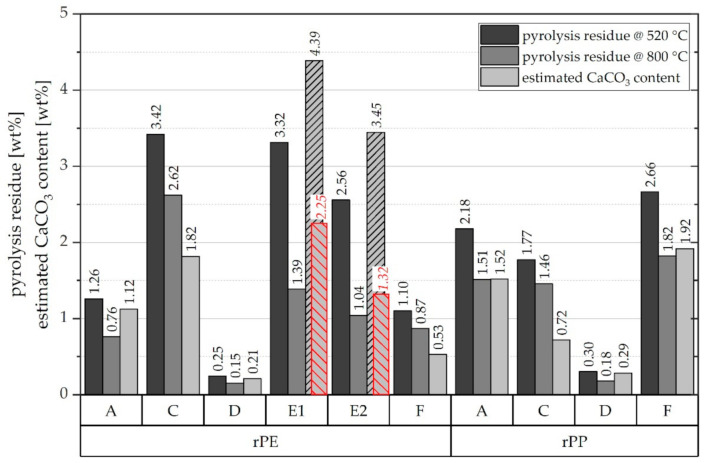
Mean values (*n* = 2) of pyrolysis residue at 520 °C and 800 °C as well as estimated content of CaCO_3_ for all investigated rPE and rPP materials.

The pyrolysis residue right after polymer decomposition at 520 °C, the ‘final’ pyrolysis residue at 800 °C, and the estimated amount of CaCO_3_ are illustrated in Figure 7.

Average pyrolysis residue levels range from 0.3 to 3.4 wt.%. A value below 1 wt.% seems to be the exception as it was found in one rPE and one rPP grade only, notably from the same supplier (D). The findings further indicate that calcium carbonate is definitely a prominent—if not the dominant—fraction among the inorganics found in rPE and rPP materials. In the analyzed samples, it constitutes between 41% and 94% of the pyrolysis residue. However, it is crucial to consider the shape of the TGA curve between 500 °C and 800 °C. Applying the calcium carbonate estimation method described above in a straight-forward manner to rPE-E1 and rPE-E2 obviously produces misleading results. The obtained estimates of 4.39 and 3.45 wt.% of CaCO_3_ for rPE-E1 and rPE-E2, respectively, are higher than the actual pyrolysis residues initially determined. The respective bars in Figure 7 are hence shaded. The reason for this mismatch is that the observed loss of mass of rPE-E1 and E2 at temperatures above 500 °C cannot be allocated to the cleavage of CO_2_ from CaCO_3_ alone. While this reaction likely does take place too, because ATR-FTIR measurements indicate the presence of CaCO_3_ (see small peaks at 875 cm^−1^ in Figure 1d), there is at least one more process involved which is probably responsible for the different shape of the rPE-E1 and E2 curves visible in Figure 6b. As these two materials are the only ones that are black (see Figure 3), it seems plausible to assume the presence of a typical pigment such as carbon black.

Usually, carbon black should remain unaffected in a TGA conducted under pyrolytic conditions, i.e., with nitrogen as a purge gas. However, the carbon black might have reacted with residual oxygen in the pyrolysis residue or it might have been sucked off from the open ceramic crucible leading to the observed linear loss of mass. A consecutive flushing with pure oxygen for 10 min at 800 °C resulted in a very moderate further loss of mass of 0.13 and 0.30 percentage points down to 1.23 and 0.74 wt.% for rPE-E1 and E2, respectively, compared to the residual mass at 800 °C in nitrogen. Taking these new values and assuming they represent CaO (the residual solid product of CaCO_3_ decomposition) would result in more plausible estimates of the CaCO_3_ contents of 2.25 and 1.32 wt.% for rPE-E1 and E2, respectively. These values are illustrated as red dashed bars in Figure 7. However, the real CaCO_3_ content could be lower than that due to the presence of other thermally stable inorganic compounds. This uncertainty is a disadvantage of using the final residual mass instead of the delta m between 520 °C and 800 °C.

### 3.2. Technological Characterization

**Figure 8:**
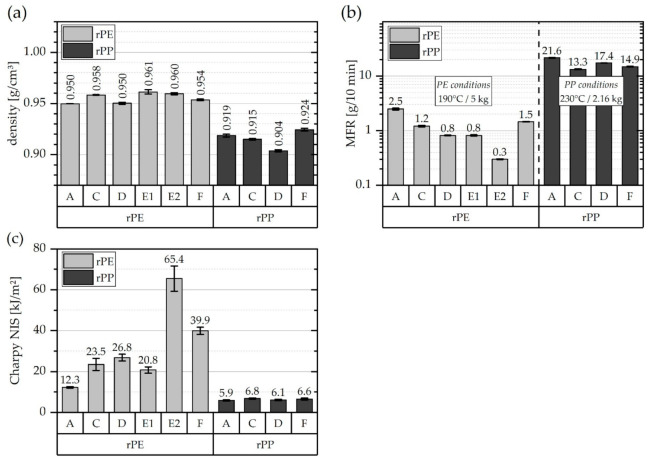
Graphical illustration of (**a**) density values, (**b**) melt flow rate (MFR) values, and (**c**) Charpy notched impact strength (NIS) values of all investigated rPE and rPP materials, respectively.

This section deals with the characterization of properties, that are of high technological relevance for polyolefin materials. These properties are summarized in Figure 8 and include (a) the density of the resin according to ISO 1183 [46], (b) the melt flow rate (MFR) at standard testing conditions according to ISO 1133 [47], and (c) the Charpy notched impact strength (NIS) according to ISO 179 [48] at 23 °C.

The rPE materials have density values between 0.950 g/cm^3^ (rPE-A & rPE-C) and 0.961 g/cm^3^ (rPE-E1). The average density over all rPEs is 0.956 g/cm^3^. There is little variation between suppliers and grades. Furthermore, these numbers are within the range typically expected for unfilled high-density polyethylene (PE-HD) resins [19,20]. The density values of the rPP materials varied between 0.904 g/cm^3^ (rPP-D) and 0.924 g/cm^3^ (rPP-F). The average density over all rPPs is 0.916 g/cm^3^. With the exception of rPP-D, these numbers are situated rather at the top end of the density value range typically expected for unfilled PP resins [16,20]. The spread between lowest and highest density is slightly higher in the rPP group than in the rPE group. Moreover, it is worth mentioning that for both resin families, rPE as well as rPP, the highest densities were measured for grades that exhibited a higher amount of inorganics, expressed as the pyrolysis residue at 520 °C, as depicted in Figure 6b and Figure 7. Lower density values were determined for grades that exhibited lower pyrolysis residues in the TGA.

The results from MFR testing are plotted in Figure 8b. Note the logarithmic representation of the vertical axis. Materials of the rPE group have been tested under typical PE testing conditions (190 °C, 5 kg), whereas the rPP materials have been tested under typical PP testing conditions (230 °C, 2.16 kg) [47]. All rPEs can be characterized as high-viscosity PEs with relatively low MFR values ranging from as low as 0.3 g/10 min (rPE-E2) up to 2.5 g/10 min (rPE-A). While this is little difference numerically, it is actually about one order of magnitude. As a matter of facts, MFR values are best compared on a logarithmic scale. There is hence a considerable difference among different rPE grades and suppliers.

The rPPs materials reached MFR values between 13 g/10 min (rPP-C) and 22 g/10 min (rPP-A). While this is a relevant difference in absolute terms, the difference on a logarithmic scale is almost negligible (see. Figure 8b). Hence, the rPP grades investigated in this work can all be characterized as rather easy-flowing PP grades suitable for standard injection molding operations. This is in agreement with the statements made by the respective recyclate suppliers who were asked to provide low-MFR PE-HD grades and medium to high-MFR PP grades.

Overall, the standard deviations observed in MFR testing were rather low. Furthermore, MFR testing is a simple but potent method for subjective quality assessments, especially when dealing with recycled plastics [60]. In the present case, the extruded MFR strands showed relatively smooth surface characteristics, no die clogging occurred, and no excessive formation of bubbles, fumes, or irritating odor was observed during MFR tests, all of which can be indicators of poor recyclate quality [60].

The Charpy notched impact strength (NIS) at room temperature is illustrated for all analyzed recyclates in Figure 8c. The rPE materials exhibited Charpy NIS values ranging from 12 kJ/m^2^ (rPE-A) up to 65 kJ/m^2^ (rPE-E2). The specimens of rPE-E1 showed a hinge break type of failure, while specimens of rPE-F and rPE-E2 showed hinge break and occasional partial break. Contrary to that, rPE-A, rPE-C, and rPE-D as well as all rPP materials exhibited complete break. The respective Charpy NIS values of the rPPs are significantly lower than those of the rPEs and range from 5.9 kJ/m^2^ (rPP-A) to 6.8 kJ/m^2^ (rPP-C). Comparing these values to the data listed in Table 1, it seems that the rPE materials can compete with typical virgin PE-HD grades, while the rPP materials are rather situated at the lower performance end in terms of impact strength of virgin resins. Notably, the Charpy NIS of the rPPs shows very little variation among different suppliers, whereas the rPEs show great differences between suppliers and grades (compare for example rPE-E1 vs. rPE-E2). One explanation for these differences in impact strength may be found in the average molecular weight of the rPEs. While no direct measurements of molecular weight distributions were carried out within this work, the differences in MFR values indirectly suggest that the rPEs differ significantly in average polymer chain length. Analogously, the similar MFR values of the rPPs (on a logarithmic scale) would indicate rather similar average molecular weight and this would fit well to the rather narrow range of Charpy NIS values. However, besides molecular mass and polydispersity, Charpy NIS of PP is also dependent on polymer architecture (co-monomers, tacticity, and chain defects) and micro-structural morphology [16,61,62] as well as (legacy) fillers and stress-concentrating defects [41,60].

### 3.3. Structure–Property Relationships

This section deals with structure–property relationships and correlations derived from the data discussed in Section 3.1 and Section 3.2. Emphasis is put on correlations of technological properties of polyolefins including density, MFR, and Charpy NIS among each other as well as with relevant compositional characteristics such as inorganics content and polyolefin cross-contamination.

**Figure 9:**
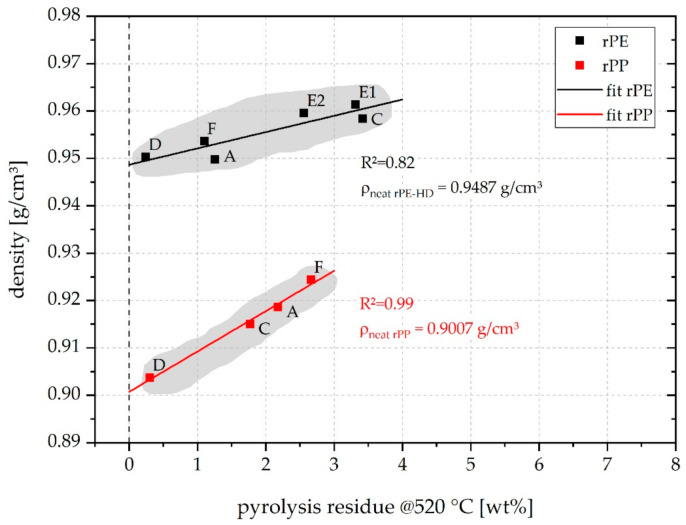
Plot of density vs. pyrolysis residue at 520 °C of all analyzed recyclates, including linear fit functions.

A correlation was sought between the recyclate density and the pyrolysis residue determined via TGA which was assumed to be the content of inorganics stemming from contamination (dirt) and/or legacy fillers. A linear regression was applied to fit the data of both the rPE and the rPP materials. The resulting plot is shown in Figure 9. The recyclate density is clearly affected by the content of inorganics. This seems plausible as all inorganic fillers typically used for polyolefins and especially calcium carbonate which was identified by means of ATR-FTIR and TGA (see Section 3.1) have higher densities than both PE and PP [63].

The R^2^ obtained from fitting the rPE data was only about 0.82. Nevertheless, the value derived for neat, i.e., unfilled, rPE-HD by extrapolation to zero pyrolysis residue was 0.9487 g/cm^3^. This is within the range of typical density values of PE-HD resins having a degree of crystallinity of about 65% [20]. Interestingly, when rPE-A, C, and E1 are excluded from the linear regression, the correlation becomes significantly better, reaching an R^2^ of 0.9998. This way, a very similar value for the extrapolated neat rPE-HD density of 0.9492 g/cm^3^ is obtained. rPE-A, C, and E1 are the three grades for which a PP melting event could be observed and corresponding PP melting enthalpies could be determined in the DSC. This highlights that both inorganics content and polyolefin cross-contamination, even at seemingly low nominal levels of a few percent by mass, significantly affect overall recyclate density. This finding might be of particular interest for rPE materials, because besides the MFR, the density is one of the prime characteristics to differentiate PE grades in industrial practice [19,20] and relevant standardization [64].

The linear regression applied to the rPP data yielded an R^2^ of 0.99. The neat rPP density obtained by extrapolation to zero pyrolysis residue was derived as 0.9007 g/cm^3^. This is in very good agreement with the typically expected density of virgin PP [16]. Interestingly, the slope of the rPP density fit function is much steeper than that of the rPE data. This is likely due to the fact that the rPP materials contain some PE-HD as shown in Section 3.1. In rPP, this polyolefin cross-contamination by PE-HD tends to further increase the overall density of the recyclate on top of the effect of inorganics fillers, while the opposite is the case for rPE-HD contaminated by PP, because PP has a lower density than PE-HD.

**Figure 10:**
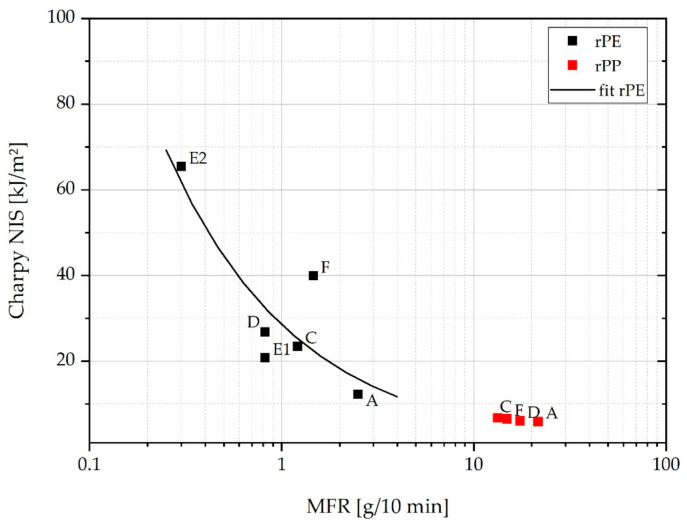
Plot of Charpy NIS vs. MFR of all analyzed recyclates in semi-logarithmic representation.

In practice, there is an entire array of parameters ranging from molecular architecture to morphology and presence of rigid or elastomeric phases that can have a pronounced influence on the impact strength of polyolefins. A particularly fundamental structure–property relationship exists between toughness and the mean molecular mass (or the molecular mass distribution as a whole) [16,62]. The latter is inversely related to MFR. The respective data of Charpy NIS and MFR of all investigated rPE and rPP grades is plotted in a log-linear representation in Figure 10. Indeed, an inverse correlation can be observed, whereas the rPE data are significantly more scattered than the rPP data. To some extent, this can be explained by the fact that the rPE data cover a much wider range of Charpy NIS values, from 12 to 65 kJ/m^2^, while the four rPP grades show essentially the same impact strength of roughly 6 to 7 kJ/m^2^. In addition, the rPE materials are more diverse in terms molecular structure as demonstrated by the wide split of their MFR values in a logarithmic representation.

**Figure 11:**
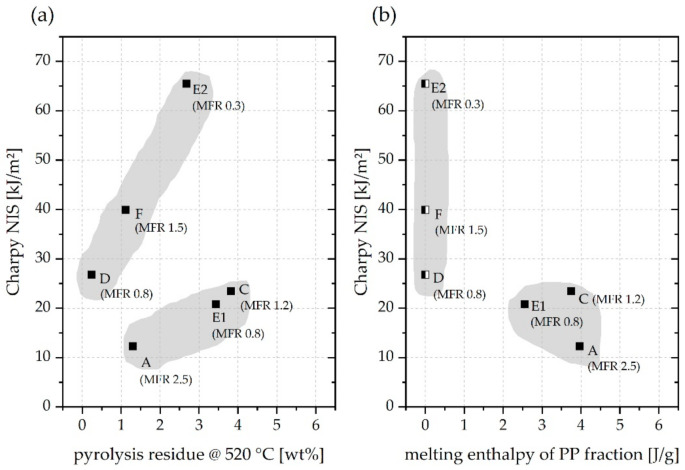
Plot of (**a**) Charpy NIS vs. pyrolysis residue at 520 °C and (**b**) Charpy NIS vs. melting enthalpy of the PP fraction of rPE materials. Only rPE data are shown. Half-filled squares indicate the melting enthalpy of PP could not be determined.

Drawing specific attention to the impact performance of the rPE materials, it seems reasonable to consider compositional aspects of these materials on top of the influence of MFR. A plot of Charpy NIS versus pyrolysis residue at 520 °C is depicted in Figure 11a. There seems to be no detrimental effect of a certain level of inorganics content. As a matter of facts, those grades of rPE with more pyrolysis residue even achieved higher Charpy NIS values. It is true that mineral fillers, especially calcium carbonate, can significantly enhance impact toughness [63]. However, a strong toughening effect is usually observed either at (much) higher mineral loading levels than determined in the present work [63,65] or when a very fine nanocomposite-like dispersion is achieved [66]. Regardless of that, neither MFR (Figure 10) nor the level of inorganics (Figure 11b) seem to sufficiently explain the apparent clustering of rPE-A, C, and E1 on the one hand and rPE-D, E2, and F on the other hand, as illustrated by the grey areas.

Figure 11b depicts a plot of Charpy NIS versus melting enthalpy of the PP fraction within the rPE materials. This representation suggests that comparatively high Charpy NIS values above 25 kJ/m^2^ are only achieved by rPE grades which combine both, a low MFR value and a (very) low level of polyolefin cross-contamination by PP. In the cases of rPE-D, E2, and F no PP melting enthalpy could be determined from DSC measurements. This per se does not guarantee total absence of PP due to limited sensitivity of DSC towards trace components, hence the half-filled squares in Figure 11b. In fact, ATR-FTIR spectroscopy and ATR-FTIR imaging revealed traces of PP even in those grades that appear rather pure in the DSC. However, the overall level of PP contamination in rPE-D, E2, and F is likely very low. Their Charpy NIS values range from 27 to 65 kJ/m^2^, whereas the lowest value is not associated with the highest MFR, because rPE-F deviates from this simple ranking.

Accordingly, the presented data set seems to be too small to draw absolutely unequivocal conclusions. Ideally, rPE materials with very-low MFR values below 0.8 g/10 min and significant PP fractions should have been included in the analysis to confirm the assumption of PP contamination limiting the impact performance of rPE materials. However, such materials were not obtained from the market survey. Nevertheless, there is evidence supporting a potential correlation between PP contamination and reduced impact performance in rPE materials. A negative effect of small fractions of PP on the mechanical behavior of PE materials has been documented in studies employing virgin PE and PP grades [57]. Van Belle et al. [37] have reported a transition from a ductile to a brittle fracture behavior and low ultimate elongation in tensile tests of blends of virgin PE-HD and virgin PP even at low PP content. This confirms earlier findings by Teh [67] and Lovinger and Williams [68]. A reduction in both Charpy and Izod impact strength upon blending PP and PE-HD has also been reported by Tai et al. [69]. A very recent study [70] that specifically analyzed post-consumer PE-HD waste fractions with different levels of PP contamination supports the finding of reduced impact strength.

Although the presence of foreign polymers other than PP adds to the overall complexity of the situation discussed in the context of this work, it still seems plausible to assume that polymer purity is one of the decisive factors for good impact performance of rPE materials.

## 4. Discussion

The extensive characterization of rPE and rPP materials discussed herein demonstrates that commercial PO recyclates are complex multi-component systems. Polyolefin cross-contamination—i.e., PE phases in rPP materials and vice versa—is a common phenomenon. The consequences of this PO cross-contamination issue are likely manifold and seem to range at least from density bias to an apparent negative effect on (notched) impact strength of rPE materials. Moreover, the scientific literature provides evidence for further negative effects especially on mechanical properties of PO materials [37,57,70,71,72].

This being said it can be regarded as problematic that no universally accepted and widely applicable method for highly precise and reproducible quantitation of minor fractions of either PE or PP in recycled PO materials is available yet. Despite this deficiency and a remaining gap between quantitation results obtained from different methods—like DSC, FTIR spectroscopy, and nuclear magnetic resonance spectroscopy—recent studies have put more emphasis on this issue [73,74,75].

Low quantities of inorganics, especially calcium carbonate, were frequently found in commercial post-consumer recyclates. These inorganics seem to have no detrimental effect on toughness, but they cause density bias on top of deviations due to PO cross-contamination. Categorization of materials by density is a well-established concept for virgin PE materials and is of high technical and practical relevance, also from a specification point of view. In view of the results discussed herein (see Figure 9), it remains in doubt whether this concept can be readily extended to post-consumer rPE materials.

Particular attention should be drawn to the frequent occurrence of incompatible inclusions and foreign polymers within post-consumer PO recyclates. Although most rPE and rPP materials investigated in this work were treated by melt filtration according to supplier information, many particle-like objects were found inside them. Some of them were even bigger than the stated filter mesh size. These inclusions very likely act as stress concentrators, thereby reducing mechanical performance.

The literature provides some coverage of processing related aspects of melt filtration systems in polymer processing [76] and recycling [38,39,76]. Methods for calculating the pressure drop at the filter, the average residence time of the melt within the filter, or the self-cleaning efficiency [77,78], can be found there. Furthermore, it has been shown that melt filtration of recycled post-consumer PP is key to achieve higher performance levels, e.g., in solid-state drawing [71]. However, we are not aware of any work that would systematically elaborate on the effectiveness of different melt filtration systems in removing specific contaminants, especially polymeric ones. It seems too simplistic to assume that the maximum size of inclusions and defects encountered in a mechanically recycled plastic such as rPE or rPP is roughly equivalent to the mesh size of the used filter. This neither accounts for the deformability, i.e., the elastic and viscous behavior of disperse polymer phases at typical melt processing temperatures and pressure gradients in recycling operations. Deformable droplets and particles might be squeezed through the filter. Nor does it account for potential particle coalescence of foreign polymer droplets after passing through the filter. The same applies for non-steady state operating conditions as encountered during screen changing or filter flushing (depending on the filtration system used).

Recent studies suggest that the presence of inclusions and defects in post-consumer recyclates have a severe impact on mechanical properties like ultimate strain and toughness [60,70,71]. This is even the case in sandwich structures with virgin material skin layers [41]. There is hence a need to further characterize the role of such inclusions and defects, e.g., by means of fracture mechanical methods [11,12] in order to arrive at adequate assessments of part performance, service life, and reliability/safety.

Modeling inclusions in post-consumer recyclates, especially their distribution within and their interaction with the surrounding matrix as well as their mechanical, thermo-mechanical, and fracture-mechanical behavior will be of interest in this context. Such efforts may necessitate the application of advanced characterization methods with higher spatial resolution than ATR-FTIR imaging and additional features for in-depth chemical, thermal, and mechanical investigations. Potentially interesting methods could include atomic force microscopy (AFM) [79], coupled AFM techniques such as AFM-IR [80], or micro-thermal analysis [81] to name just a few.

## 5. Conclusions

The world is longing for more recycled plastics, recycled polyolefins from post-consumer streams in particular. This brings about the need to create robust understanding of the characteristics and qualities of such materials. Compositional analysis and establishment of structure–property relationships are two fundamentally necessary elements in the quest for tailor-made recyclates that will be required for a wide range of applications.

## Figures and Tables

**Figure 4:**
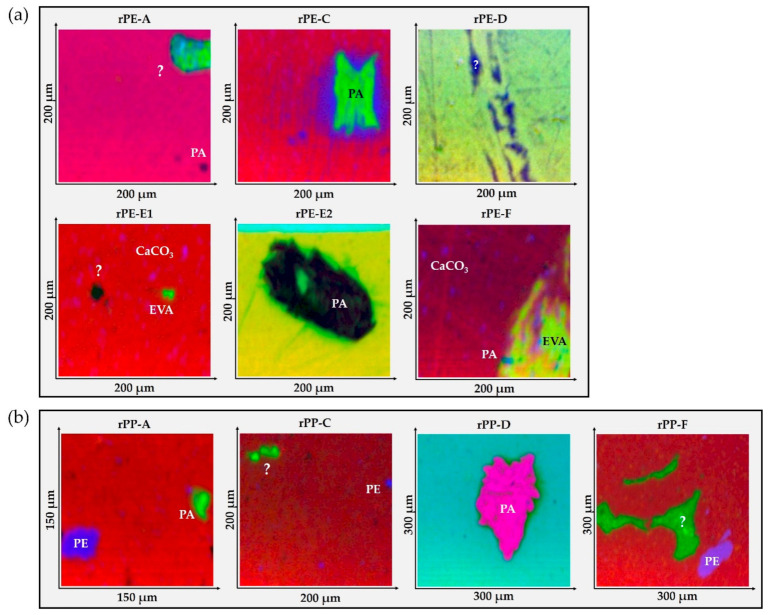
Collection of ATR-FTIR images in false color representation exhibiting typical inclusions found within the recyclates. (**a**) rPE materials and (**b**) rPP materials. Image size is between 150 × 150 µm and 300 × 300 µm. One representative image is shown per recyclate grade.

**Table 1:** Overview of relevant properties of virgin PE and PP resins including typical ranges for these properties. Data are taken from [16,19,20].

Polymer ^1^	MFR ^2^ (g/10 min)	Density (g/cm^3^)	Melting Temperature (°C)	Crystallinity (%)	Impact Strength ^3^ (kJ/m^2^)
PE-HD	<0.1–60	0.94–0.97	128–136	60–80	6–n.b.
PE-LLD	0.8–30	0.90–0.93	120–130	30–45	n.b.
PE-LD	0.4–90	0.915–0.935	105–115	40–50	n.b.
PP h. isotactic	0.3–1000	0.905–0.915	160–167	30–60	2–25
PP r.	0.3–100	0.900	135–150	not stated	5–50
PP heco	0.5–100	0.900–0.912	160–167	not stated	10–n.b. ^2^

^1^ HD, high density; LLD, linear low density; LD, low density; h., homopolymer; r., random copolymer; heco, heterophasic block copolymer. ^2^ Testing conditions are 190 °C/5.0 kg and 230 °C/2.16 kg for PE-HD and PP, respectively. ^3^ values refer to Charpy notched impact testing at 23 °C.

**Table 2:** Overview of rPE and rPP sample materials including relevant information retrieved from their respective technical data sheets.

Name	Polymer	MFR ^1^ (g/10 min)	Density ^2^ (g/cm^3^)	Ash Content ^2^ (wt.%)	Mesh Size (µm)
rPE-A	PE-HD	≤3	0.950	≤1	180
rPE-C	PE-HD	1.1–2.0	0.94–0.99	<5	not stated
rPE-D	PE-HD	1.0	0.95	≤0.5	≤100
rPE-E1	PE-HD	0.7–1.0	≥0.945	not stated	80
rPE-E2	PE-HD	0.2–0.5	≥0.945	not stated	80
rPE-F	PE-HD	1.5	0.95	not stated	not stated
rPP-A	PP	≥20	0.916	≤2	180
rPP-C	PP	10–15	0.85–0.95	<5	not stated
rPP-D	PP	18	0.90	≤0.5	≤100
rPP-F	PP	13	0.92	not stated	not stated

^1^ Testing conditions are 190 °C/5.0 kg and 230 °C/2.16 kg for PE-HD and PP, respectively. ^2^ Number of positions behind the decimal point are reported as in the respective data sheets.

**Table 3:** Mean values of melting peak temperature T_m_, melting enthalpy ∆H_m_, crystallization peak temperature T_c_, and crystallization enthalpy ∆H_c_ derived from DSC. Subscript numbers 1 and 2 indicate first and second peak observed in a given heating or cooling scan.

	T_m1_	∆H_m1_	T_m2_	∆H_m2_	T_c1_	∆H_c1_	T_c2_	∆H_c2_
	°C	J/g	°C	J/g	°C	J/g	°C	J/g
rPE-A	132.5	176.6	162.5	4.0	117.5	181.0 ^1^	-	-
rPE-C	131.9	177.7	160.4	3.7	118.2	181.5 ^1^	-	-
rPE-D	131.3	201.4	-	-	117.8	202.0	-	-
rPE-E1	131.3	173.1	159.0	2.6	117.7	177.7 ^1^	-	-
rPE-E2	131.0	179.1	-	-	117.7	181.1	-	-
rPE-F	131.5	190.8	-	-	118.1	189.2	-	-
								
rPP-A	126.1	4.5	164.0	70.7	123.6	88.7 ^2^ (79.2) ^3^	115.2	(9.5) ^3^
rPP-C	124.4	1.2	162.6	74.4	124.9	85.7 ^2^	110.3	-
rPP-D	124.5	2.1	158.9	56.7	122.1	77.9 ^2^	110.8	-
rPP-F	126.8	12.2	161.8	59.8	123.1	83.4 ^2^ (66.6) ^3^	115.8	(16.8) ^3^

^1^ Numbers refer to entire exothermal enthalpy change, including crystallization of minor PP fraction. ^2^ Numbers refer to entire exothermal enthalpy change, including a small amount from crystallization of minor PE fraction. ^3^ Numbers in brackets are derived from splitting overlapping peaks at 119 °C. Value thus obtained for ∆H_c1_ of PP fraction is likely underestimated, while ∆H_c2_ of PE fraction is likely overestimated.

## Data Availability

The data presented in this study are available in the Supplementary Material.

## Supplementary Materials

The following are available online at https://www.mdpi.com/article/10.3390/polym13101574/s1, Figure S1: ATR-FTIR imaging of rPE-A sample 1, Figure S2: ATR-FTIR imaging of rPE-A sample 2, Figure S3: ATR-FTIR imaging of rPE-C sample 1, Figure S4: ATR-FTIR imaging of rPE-C sample 2, Figure S5: ATR-FTIR imaging of rPE-D sample 1, Figure S6: ATR-FTIR imaging of rPE-D sample 2, Figure S7: ATR-FTIR imaging of rPE-E1 sample 1, Figure S8: ATR-FTIR imaging of rPE-E1 sample 2, Figure S9: ATR-FTIR imaging of rPE-E2 sample 1, Figure S10: ATR-FTIR imaging of rPE-E2 sample 2, Figure S11: ATR-FTIR imaging of rPE-F sample 1, Figure S12: ATR-FTIR imaging of rPE-F sample 2, Figure S13: ATR-FTIR imaging of rPP-A sample 1, Figure S14: ATR-FTIR imaging of rPP-A sample 2, Figure S15: ATR-FTIR imaging of rPP-C sample 1, Figure S16: ATR-FTIR imaging of rPP-C sample 2, Figure S17: ATR-FTIR imaging of rPP-D sample 1, Figure S18: ATR-FTIR imaging of rPP-D sample 2, Figure S19: ATR-FTIR imaging of rPP-F sample 1, Figure S20: ATR-FTIR imaging of rPP-F sample 2.
