# Supplementary material for: Characterization of Composition and Structure–Property Relationships of Commercial Post-Consumer Polyethylene and Polypropylene Recyclates
Source: Polymers (Basel). 2021 May 14;13(10):1574. doi: 10.3390/polym13101574 (PMC8156794; doi:10.3390/polym13101574)
Supplement: Supplementary file 1 [file polymers-13-01574-s001.zip › Supplementary Materials.pdf]

Supplementary Materials to

Characterization of composition and structure-property relationships of commercial post-consumer polyethylene and polypropylene recyclates

Markus Gall [1,*], Paul J. Freudenthaler [1], Joerg Fischer [1] and Reinhold W. Lang [1]

[1]Institute of Polymeric Materials and Testing, Johannes Kepler University Linz, Altenberger Straße 69, 4040 Linz, Austria; ipmt@jku.at
*Correspondence: markus.gall@jku.at; Tel.: +43-732-2468-6626

This file contains supplementary figures S1 to S20. These figures each show a false color representation of an ATR-FTIR image in (a) which was recorded from a location on the polished cross-section of a multi-purpose specimen made from the respective recyclate material. Section (b) of each figure depicts the summary spectra obtained from averaging all individual ATR-FTIR spectra (one per pixel) recorded within the areas marked "1" to "6". Relevant bands of the spectra are labeled with wavenumbers to facilitate chemical group assignment. However, a rather coarse spectral resolution of 16 cm^{-1} was chosen for these measurements, hence wavenumbers may deviate slightly from typical literature values. Two figures are provided per recyclate materials, i.e. sample 1 and sample 2 of rPE-A, etc.

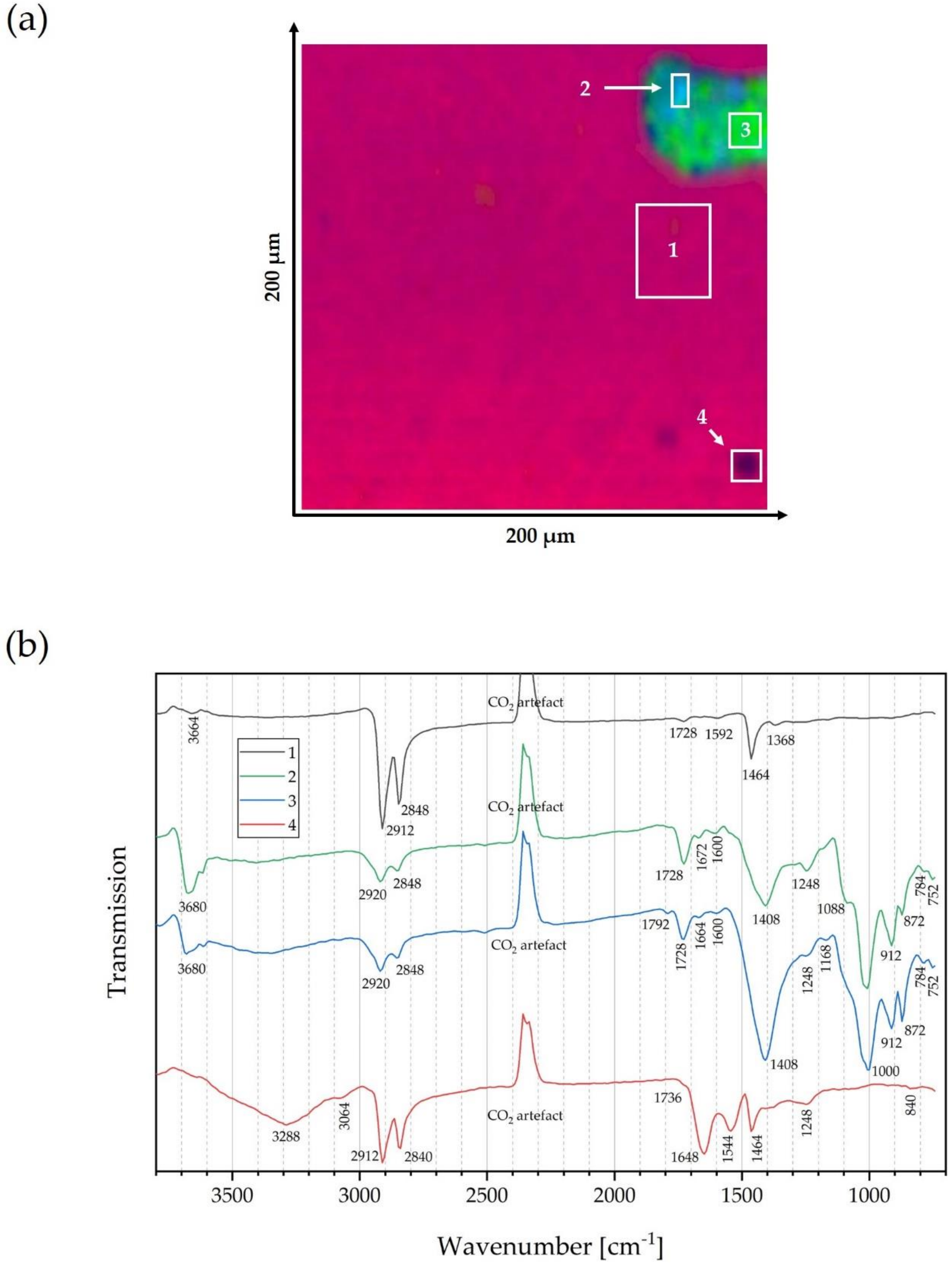

Figure S1. ATR-FTIR imaging of rPE-A sample 1. (a) ATR-FTIR image in false color representation recorded from a polished specimen cross-section exhibiting typical inclusions. This image is also shown in Fig. 4 (a). (b) Summary spectra obtained from the regions 1 to 4 shown in (a) including wavenumber assignments of relevant bands.

(a)

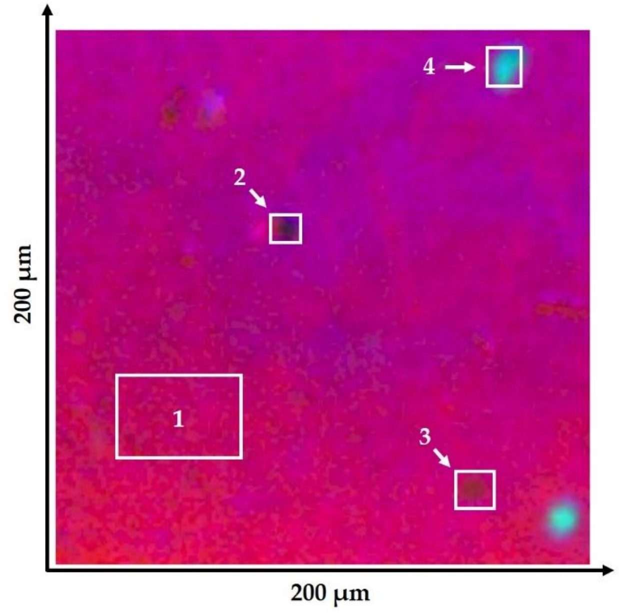

(b)

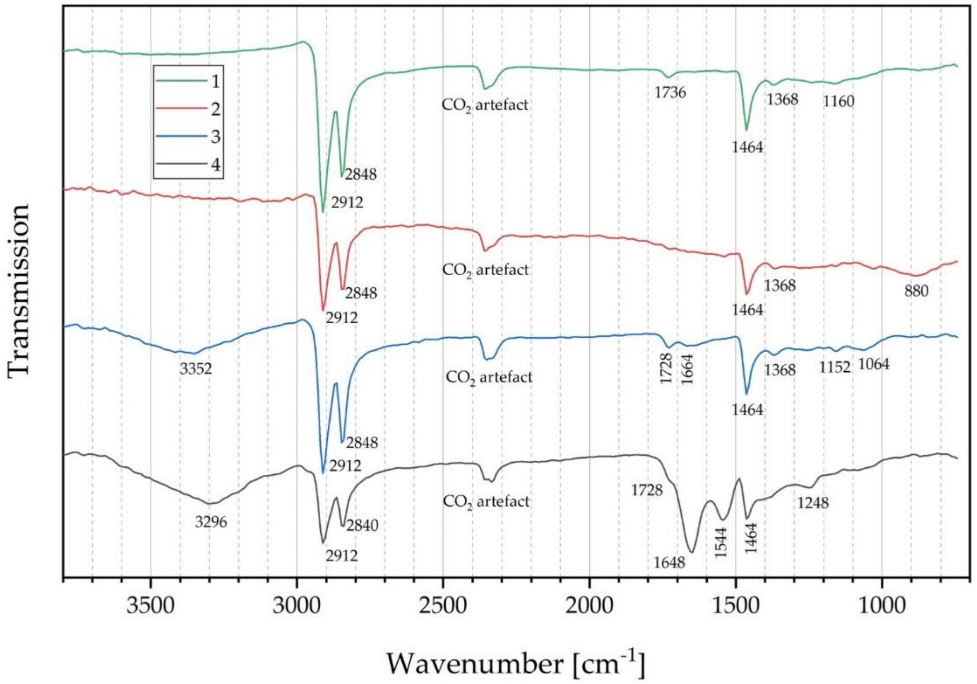

Figure S2. ATR-FTIR imaging of rPE-A sample 2. (a) ATR-FTIR image in false color representation recorded from a polished specimen cross-section exhibiting typical inclusions. (b) Summary spectra obtained from the regions 1 to 4 shown in (a) including wavenumber assignments of relevant bands.

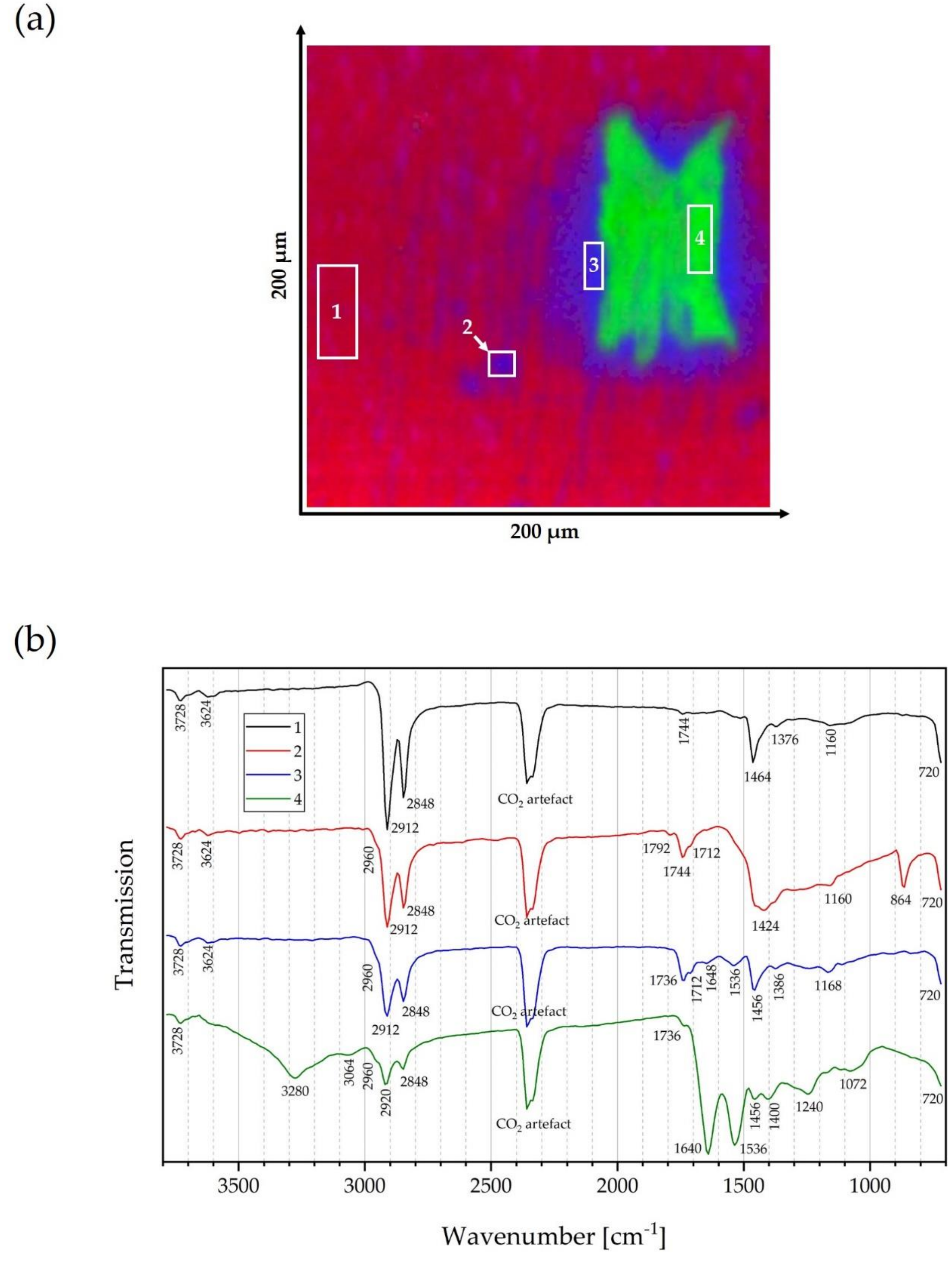

Figure S3. ATR-FTIR imaging of rPE-C sample 1. (a) ATR-FTIR image in false color representation recorded from a polished specimen cross-section exhibiting typical inclusions. This image is also shown in Fig. 4 (a). (b) Summary spectra obtained from the regions 1 to 4 shown in (a) including wavenumber assignments of relevant bands.

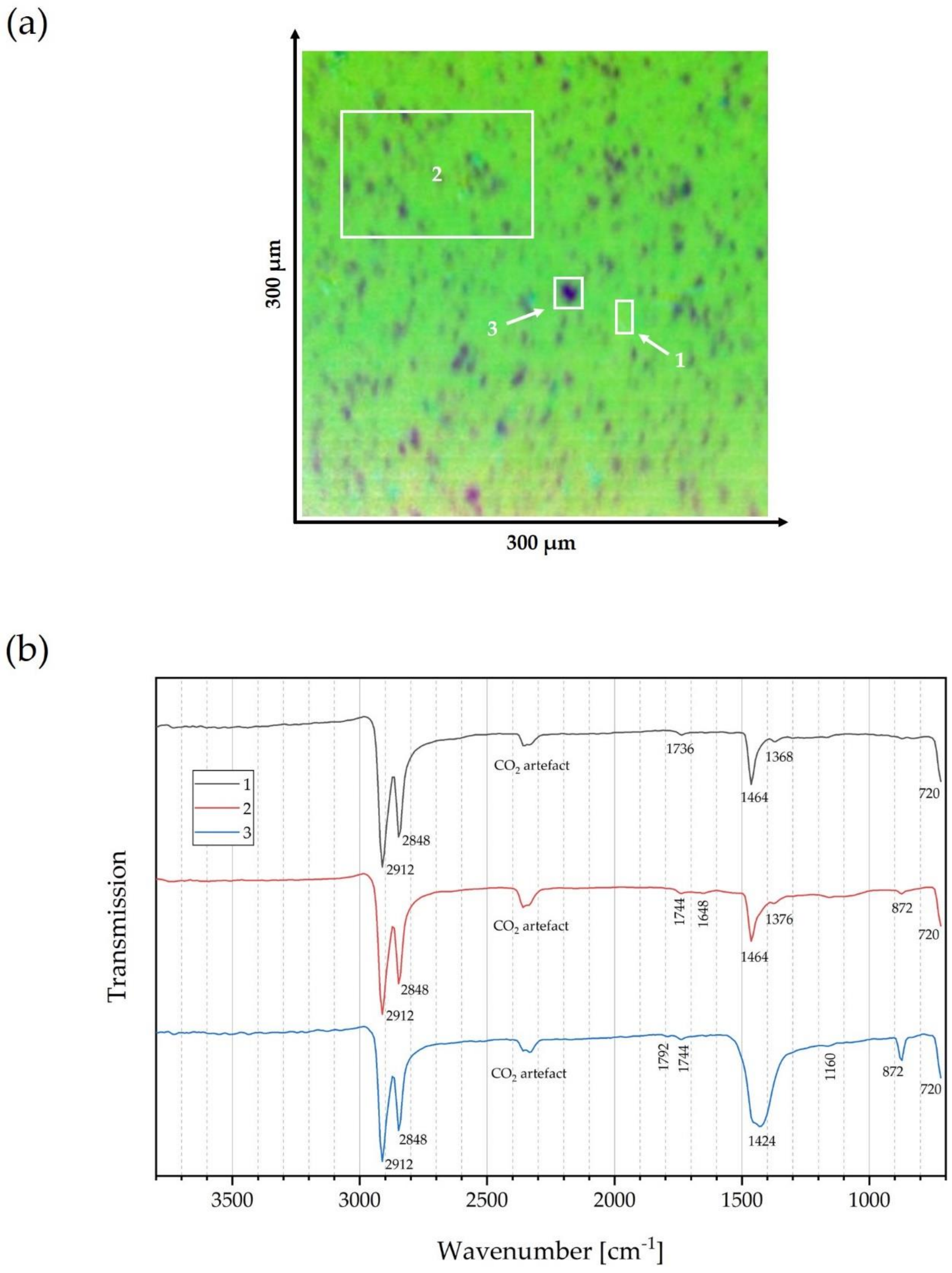

Figure S4. ATR-FTIR imaging of rPE-C sample 2. (a) ATR-FTIR image in false color representation recorded from a polished specimen cross-section exhibiting typical inclusions. (b) Summary spectra obtained from the regions 1 to 3 shown in (a) including wavenumber assignments of relevant bands.

(a)

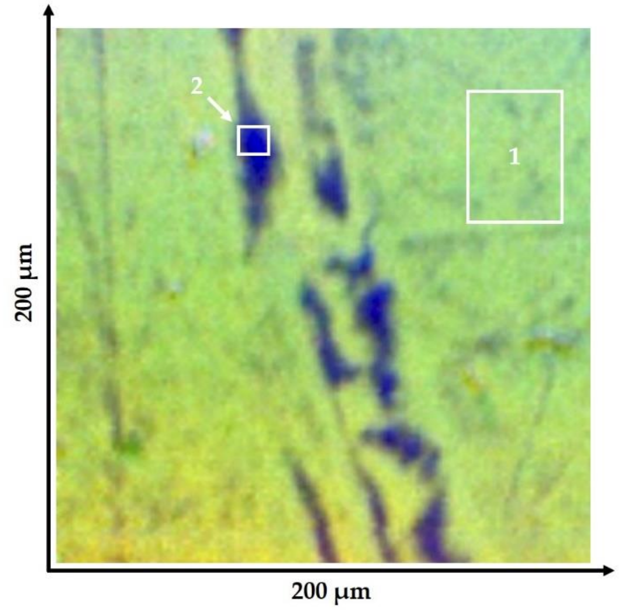

(b)

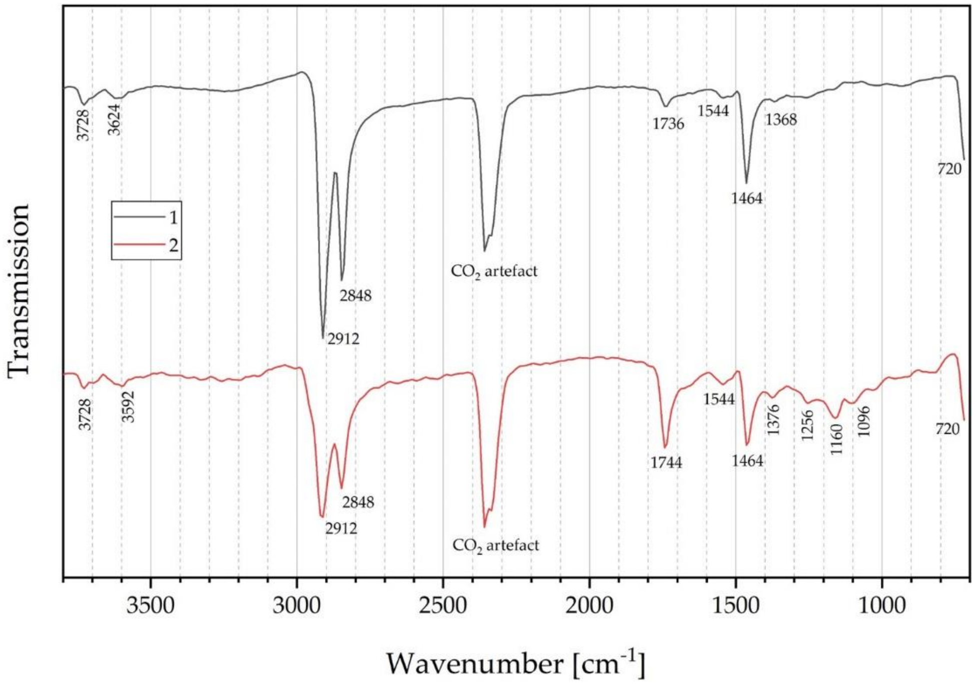

Figure S5. ATR-FTIR imaging of rPE-D sample 1. (a) ATR-FTIR image in false color representation recorded from a polished specimen cross-section exhibiting typical inclusions. This image is also shown in Fig. 4 (a). (b) Summary spectra obtained from the regions 1 to 2 shown in (a) including wavenumber assignments of relevant bands.

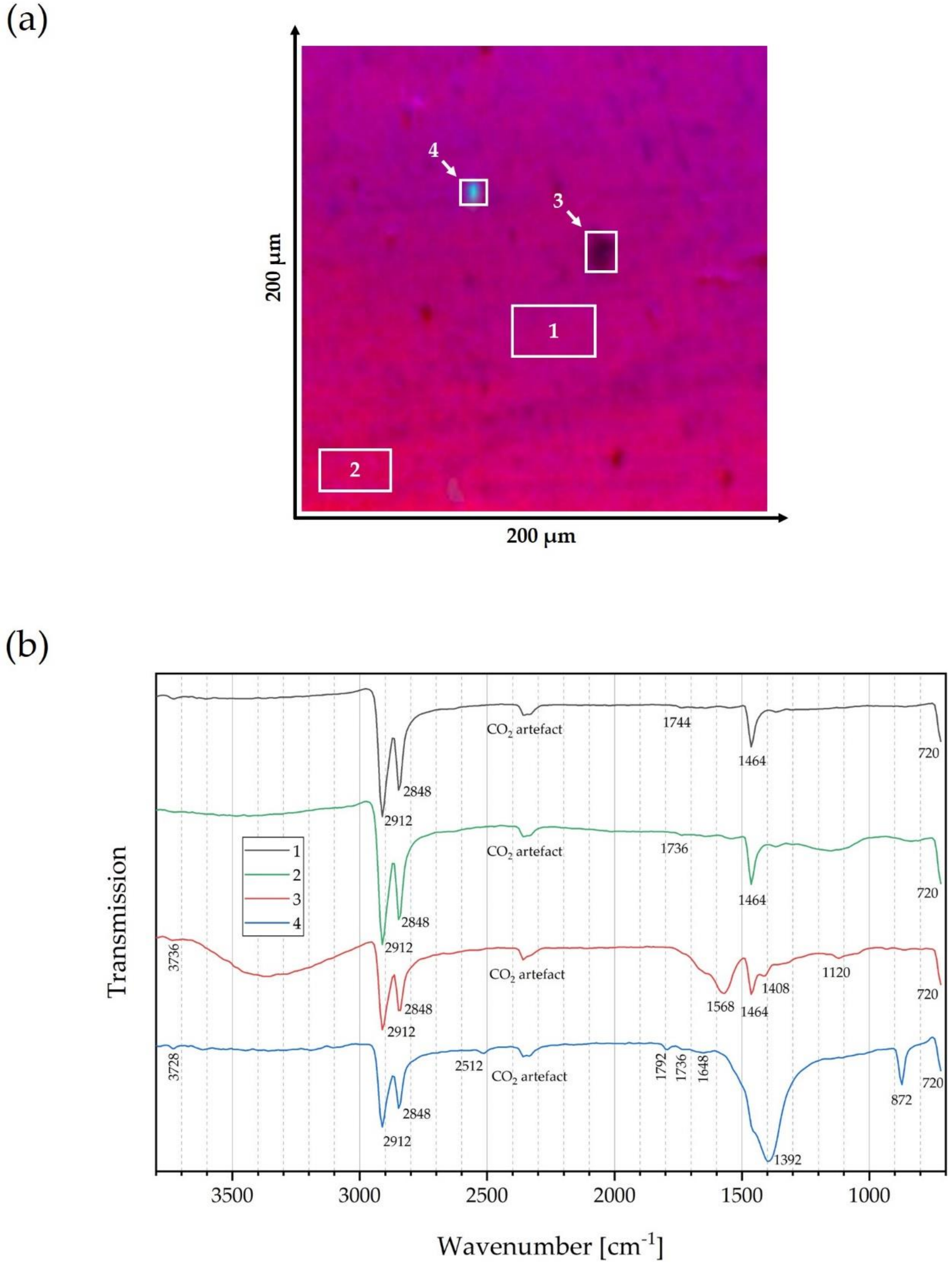

Figure S6. ATR-FTIR imaging of rPE-D sample 2. (a) ATR-FTIR image in false color representation recorded from a polished specimen cross-section exhibiting typical inclusions. (b) Summary spectra obtained from the regions 1 to 4 shown in (a) including wavenumber assignments of relevant bands.

(a)

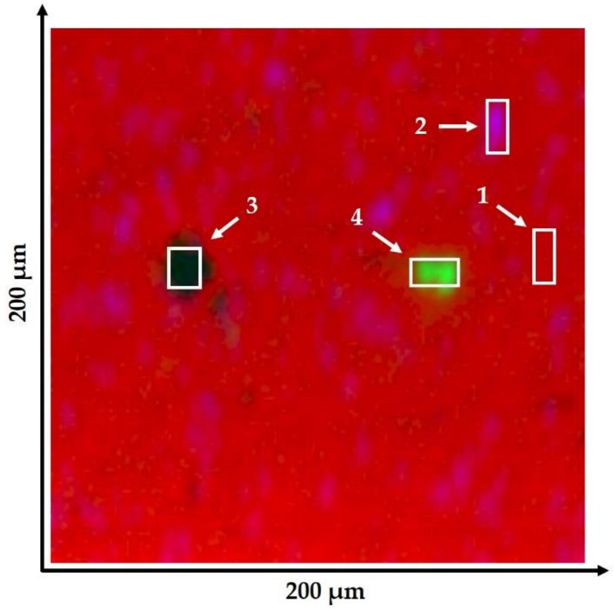

(b)

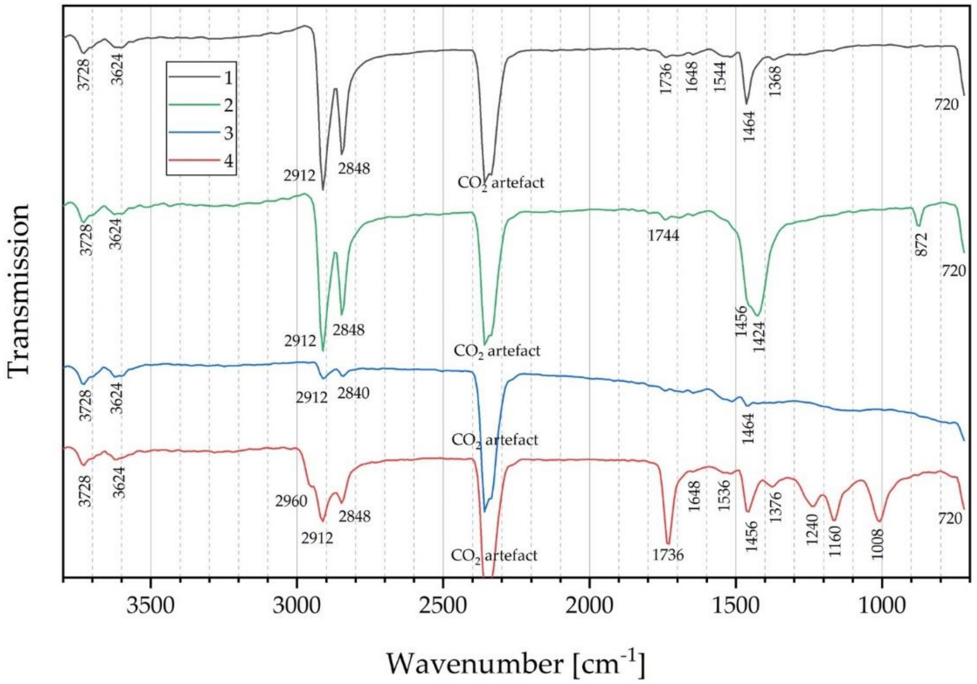

Figure S7. ATR-FTIR imaging of rPE-E1 sample 1. (a) ATR-FTIR image in false color representation recorded from a polished specimen cross-section exhibiting typical inclusions. This image is also shown in Fig. 4 (a). (b) Summary spectra obtained from the regions 1 to 4 shown in (a) including wavenumber assignments of relevant bands.

(a)

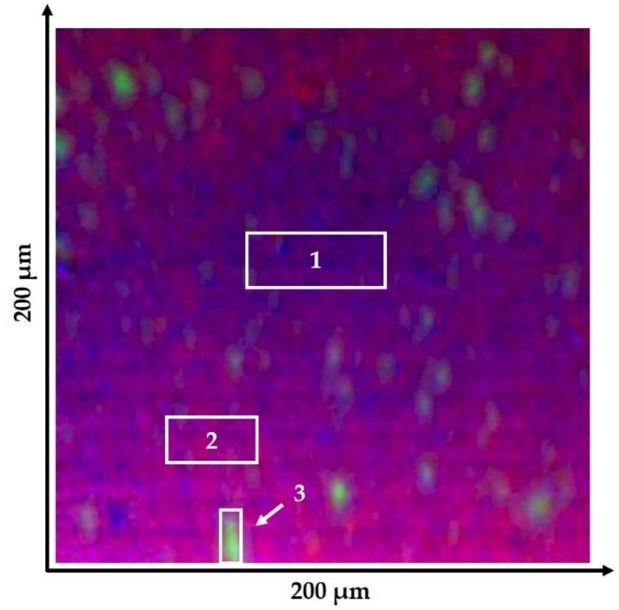

(b)

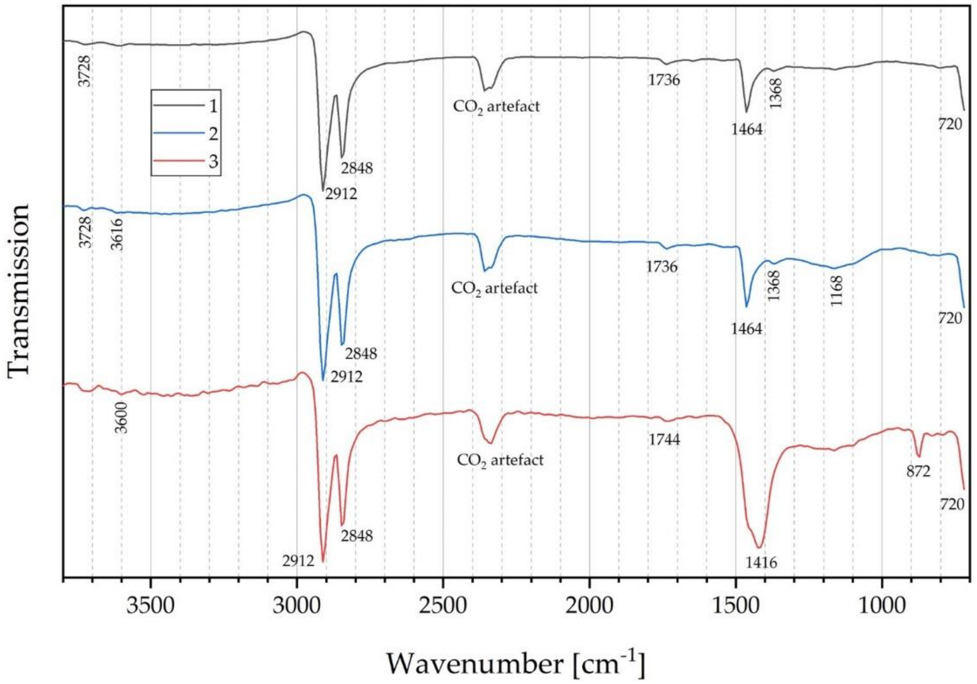

Figure S8. ATR-FTIR imaging of rPE-E1 sample 2. (a) ATR-FTIR image in false color representation recorded from a polished specimen cross-section exhibiting typical inclusions. (b) Summary spectra obtained from the regions 1 to 3 shown in (a) including wavenumber assignments of relevant bands.

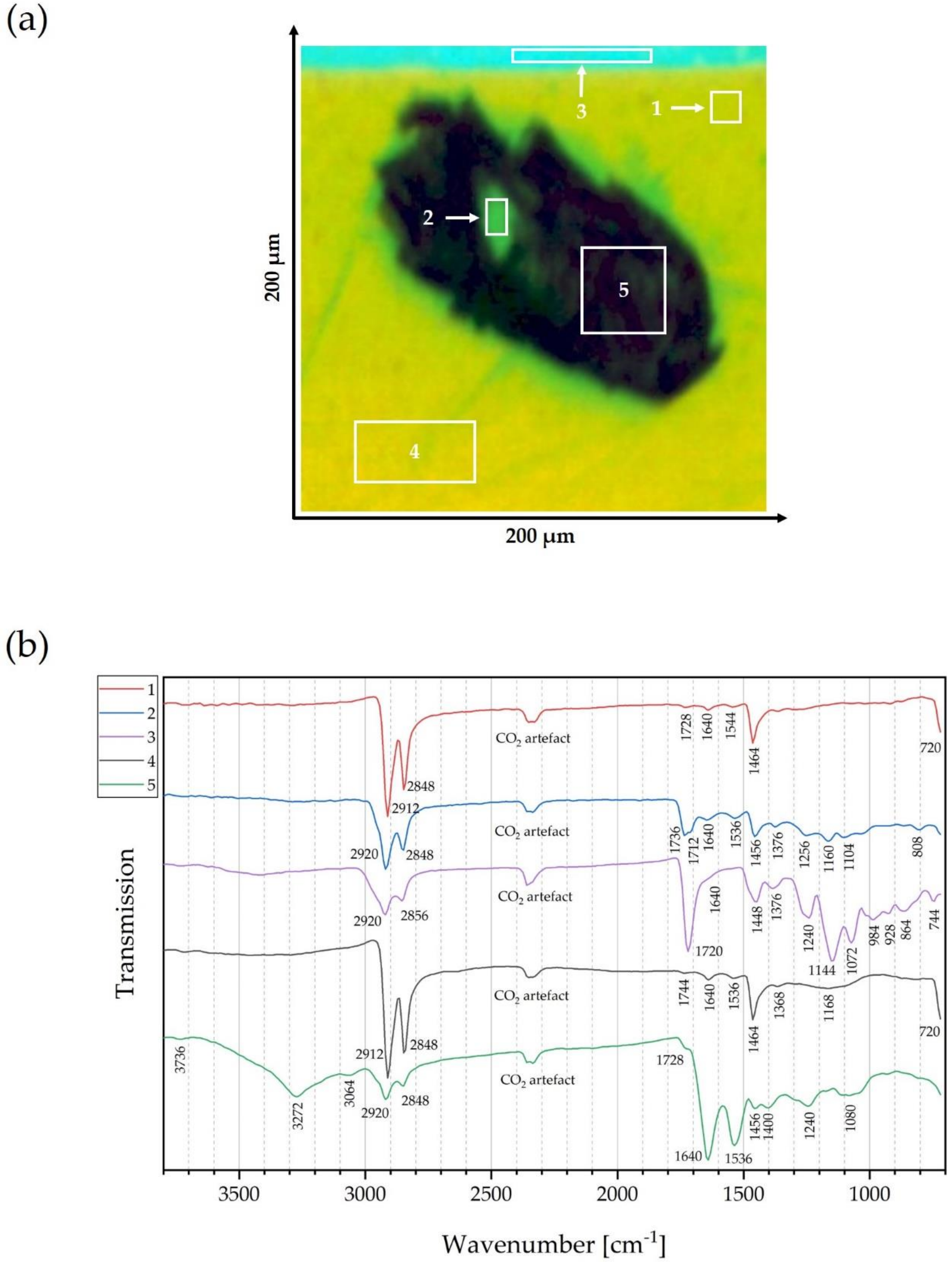

Figure S9. ATR-FTIR imaging of rPE-E2 sample 1. (a) ATR-FTIR image in false color representation recorded from a polished specimen cross-section exhibiting typical inclusions. This image is also shown in Fig. 4 (a). (b) Summary spectra obtained from the regions 1 to 5 shown in (a) including wavenumber assignments of relevant bands. Region 3 is the resin used for embedding the sample and is hence not part of the rPE-E2 material.

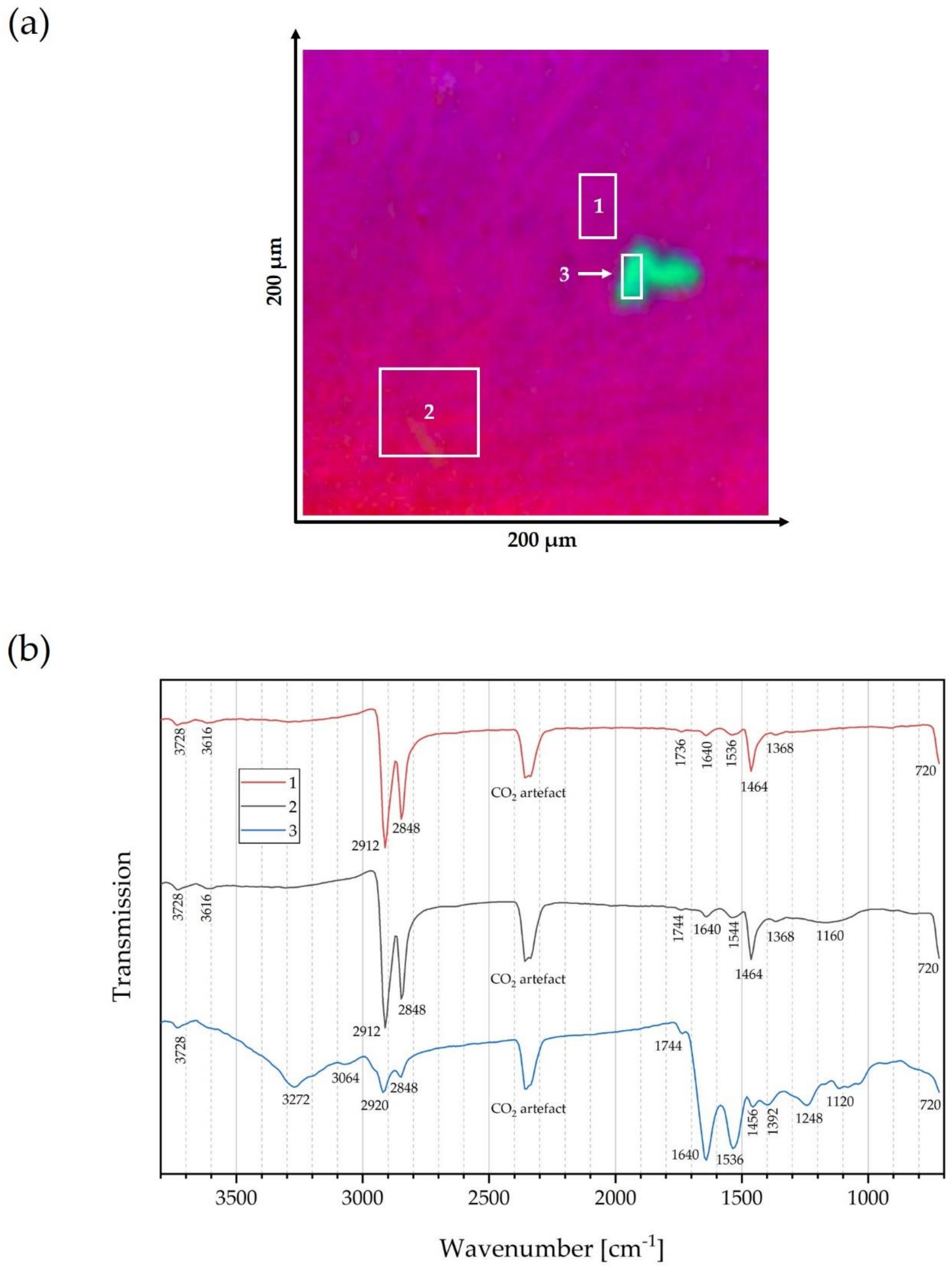

Figure S10. ATR-FTIR imaging of rPE-E2 sample 2. (a) ATR-FTIR image in false color representation recorded from a polished specimen cross-section exhibiting typical inclusions. (b) Summary spectra obtained from the regions 1 to 3 shown in (a) including wavenumber assignments of relevant bands.

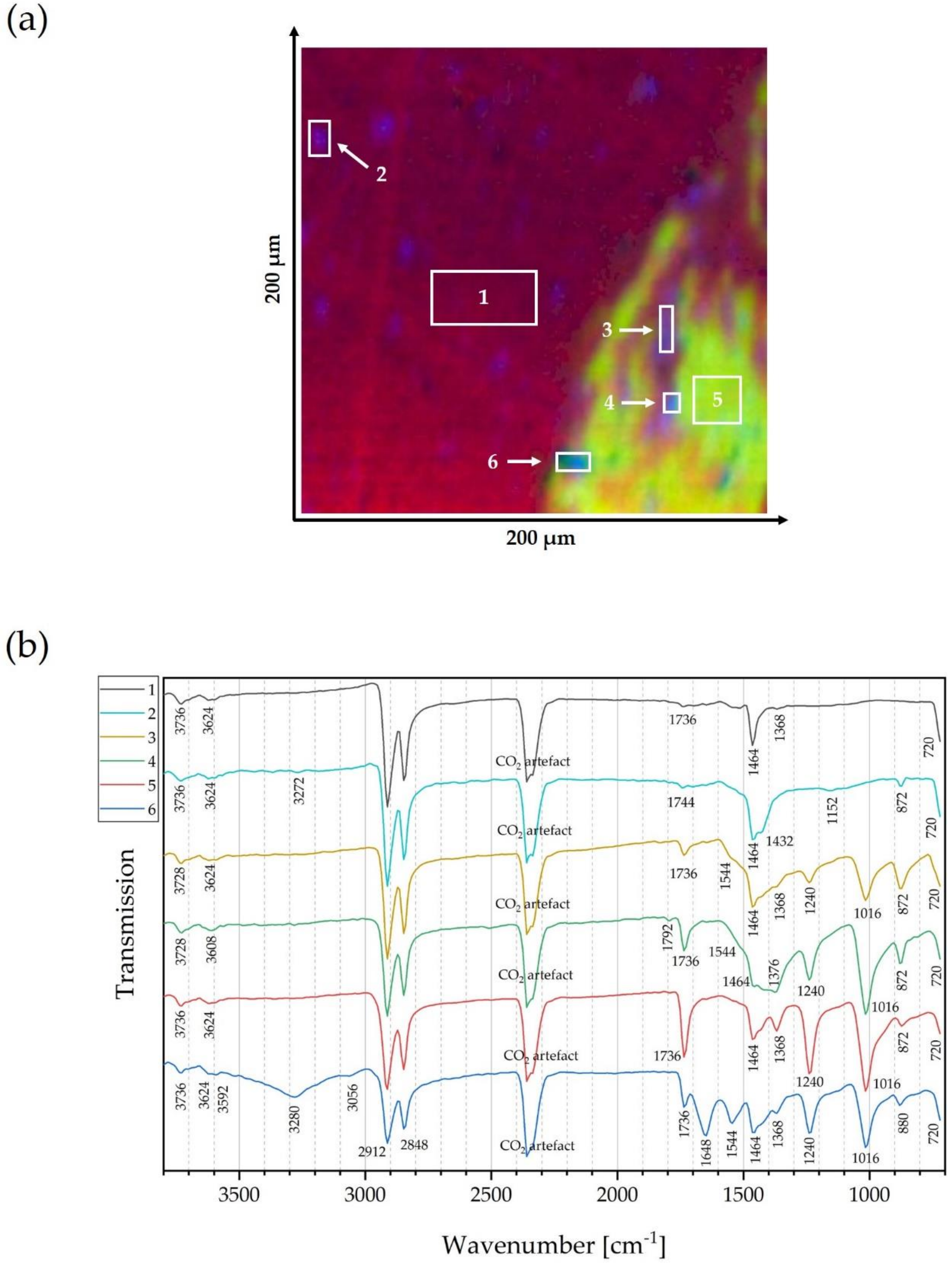

Figure S11. ATR-FTIR imaging of rPE-F sample 1. (a) ATR-FTIR image in false color representation recorded from a polished specimen cross-section exhibiting typical inclusions. This image is also shown in Fig. 4 (a). (b) Summary spectra obtained from the regions 1 to 6 shown in (a) including wavenumber assignments of relevant bands.

(a)

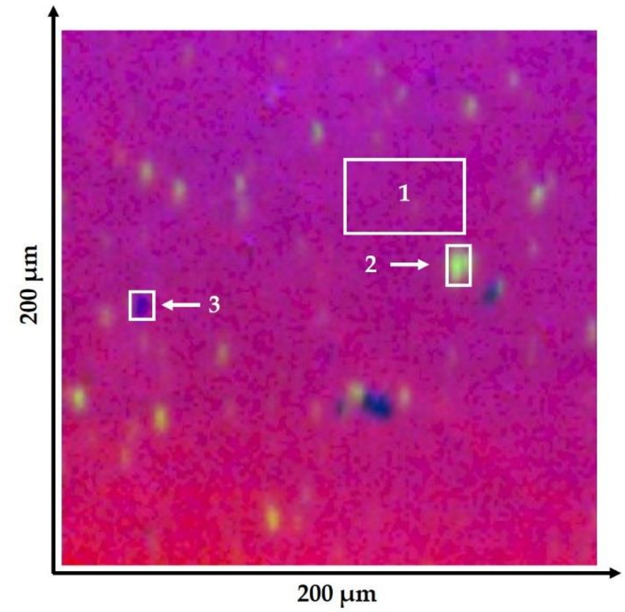

(b)

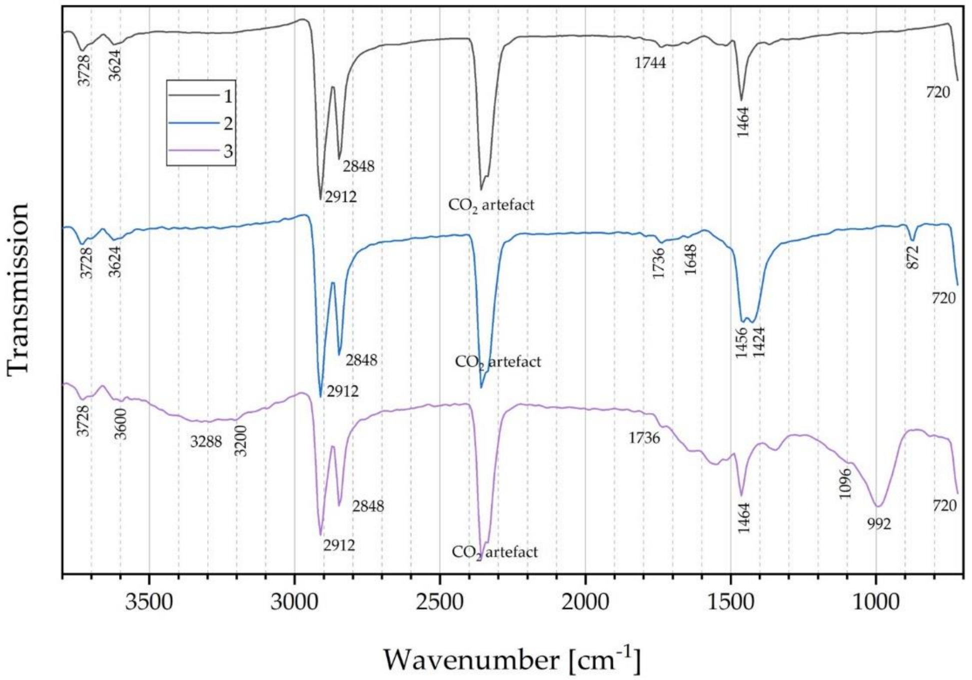

Figure S12. ATR-FTIR imaging of rPE-F sample 2. (a) ATR-FTIR image in false color representation recorded from a polished specimen cross-section exhibiting typical inclusions. (b) Summary spectra obtained from the regions 1 to 3 shown in (a) including wavenumber assignments of relevant bands.

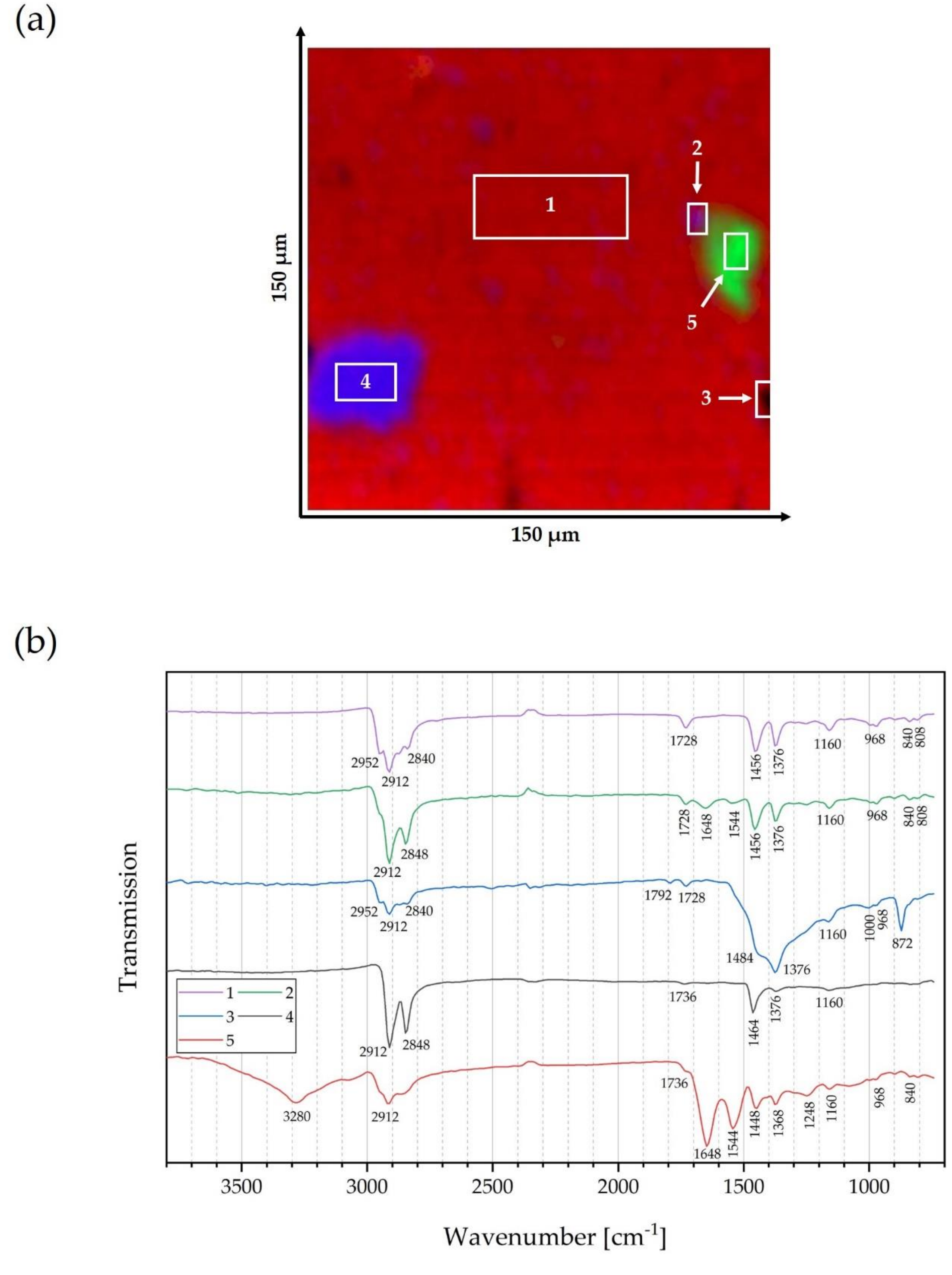

Figure S13. ATR-FTIR imaging of rPP-A sample 1. (a) ATR-FTIR image in false color representation recorded from a polished specimen cross-section exhibiting typical inclusions. This image is also shown in Fig. 4 (b). (b) Summary spectra obtained from the regions 1 to 5 shown in (a) including wavenumber assignments of relevant bands.

(a)

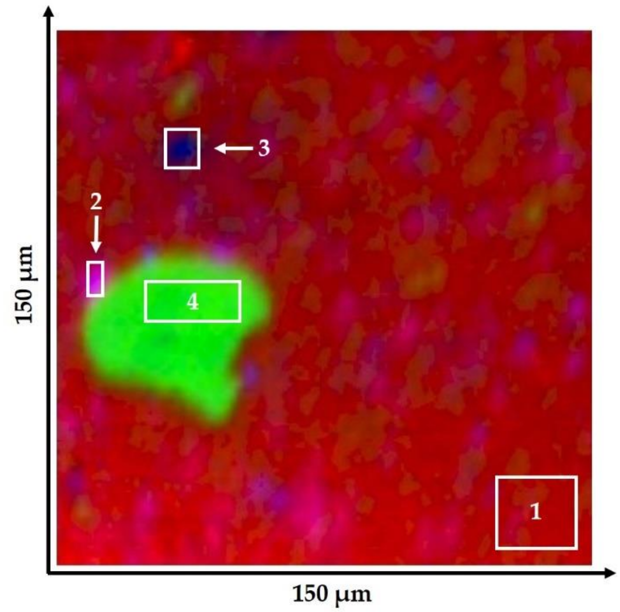

(b)

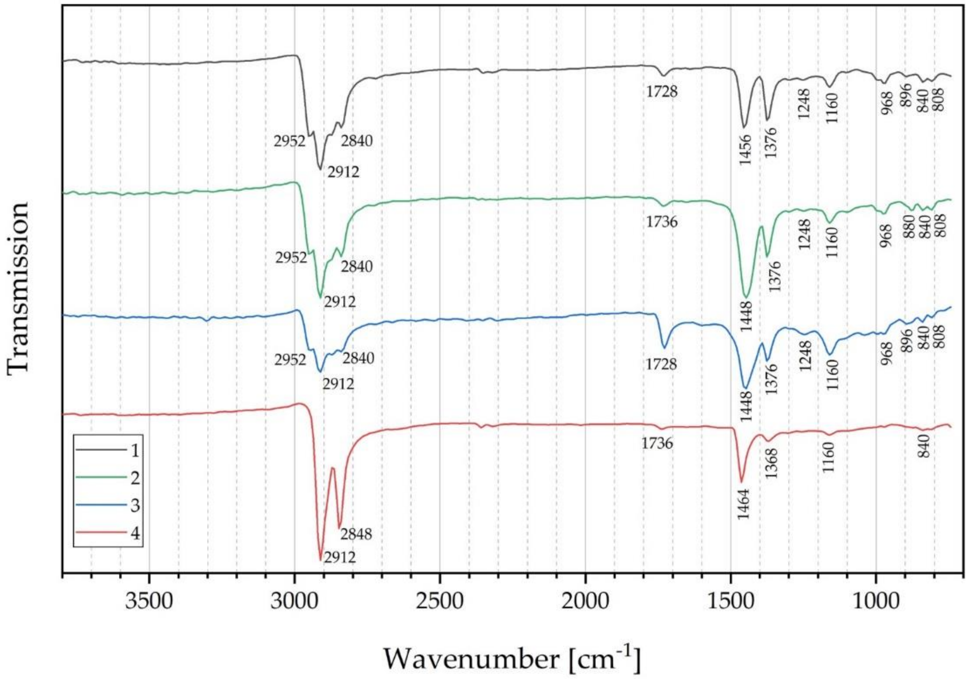

Figure S14. ATR-FTIR imaging of rPP-A sample 2. (a) ATR-FTIR image in false color representation recorded from a polished specimen cross-section exhibiting typical inclusions. (b) Summary spectra obtained from the regions 1 to 4 shown in (a) including wavenumber assignments of relevant bands.

(a)

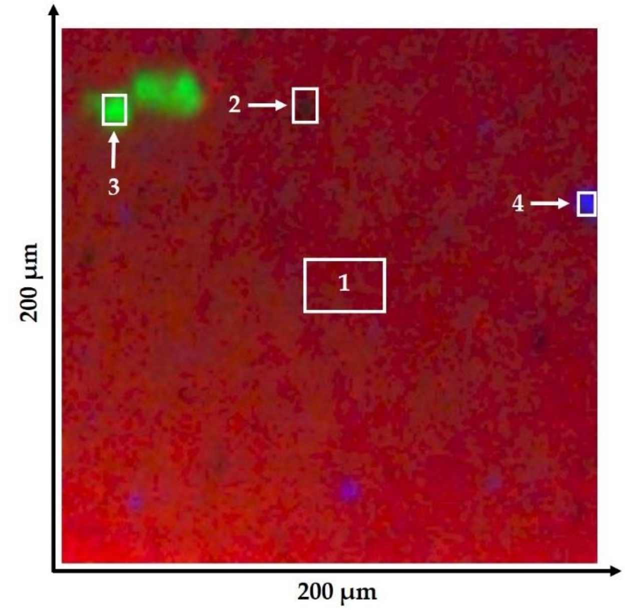

(b)

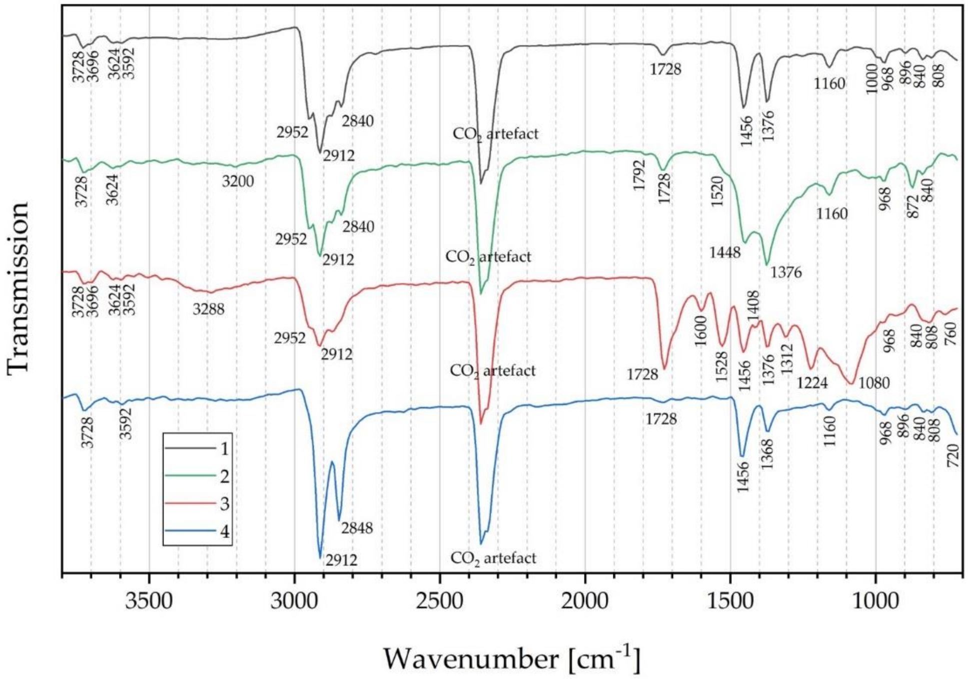

Figure S15. ATR-FTIR imaging of rPP-C sample 1. (a) ATR-FTIR image in false color representation recorded from a polished specimen cross-section exhibiting typical inclusions. This image is also shown in Fig. 4 (b). (b) Summary spectra obtained from the regions 1 to 4 shown in (a) including wavenumber assignments of relevant bands.

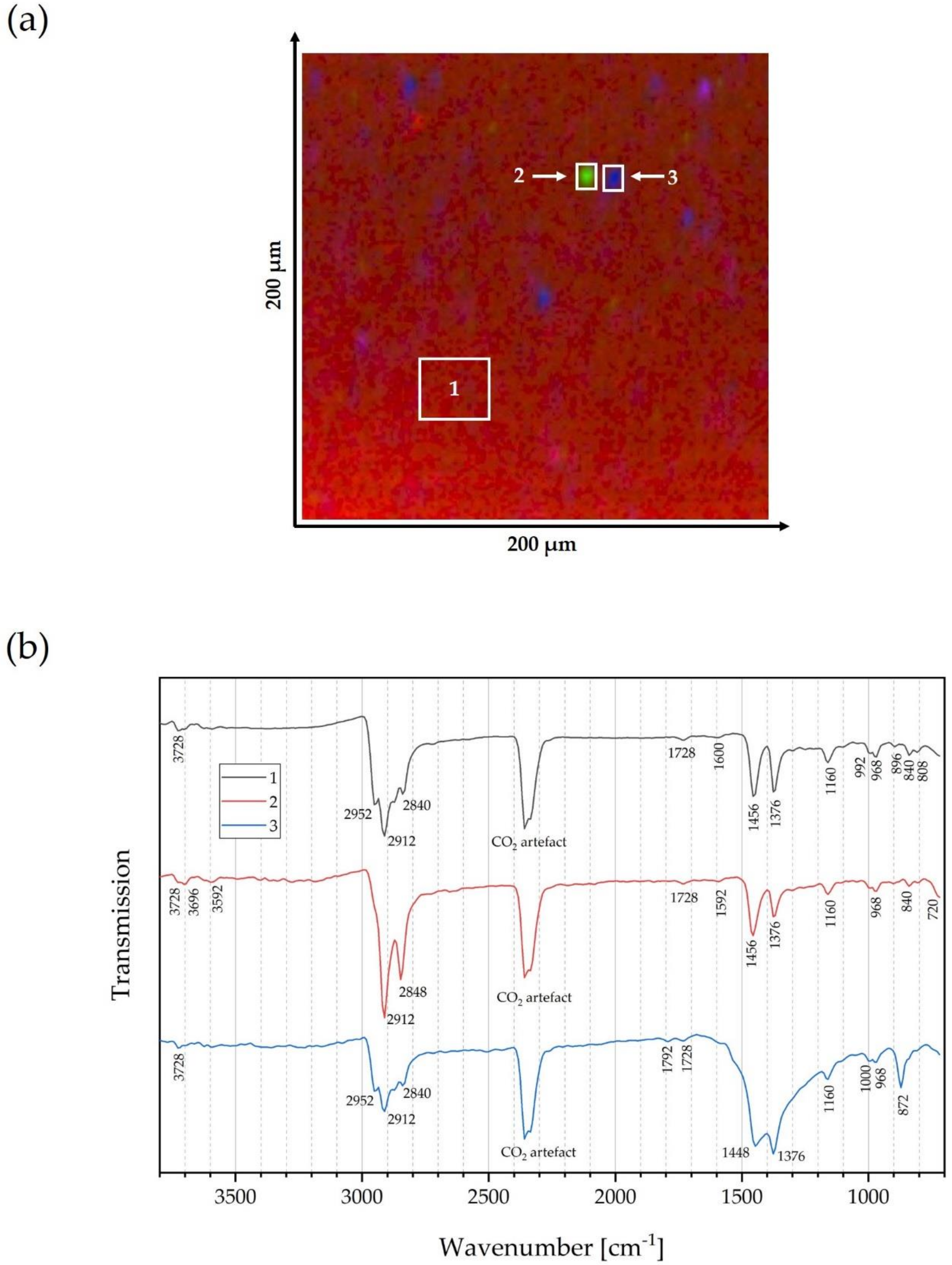

Figure S16. ATR-FTIR imaging of rPP-C sample 2. (a) ATR-FTIR image in false color representation recorded from a polished specimen cross-section exhibiting typical inclusions. (b) Summary spectra obtained from the regions 1 to 3 shown in (a) including wavenumber assignments of relevant bands.

(a)

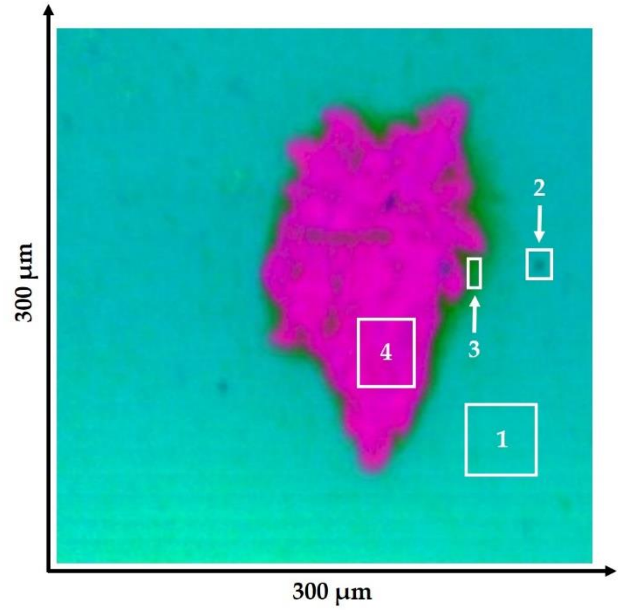

(b)

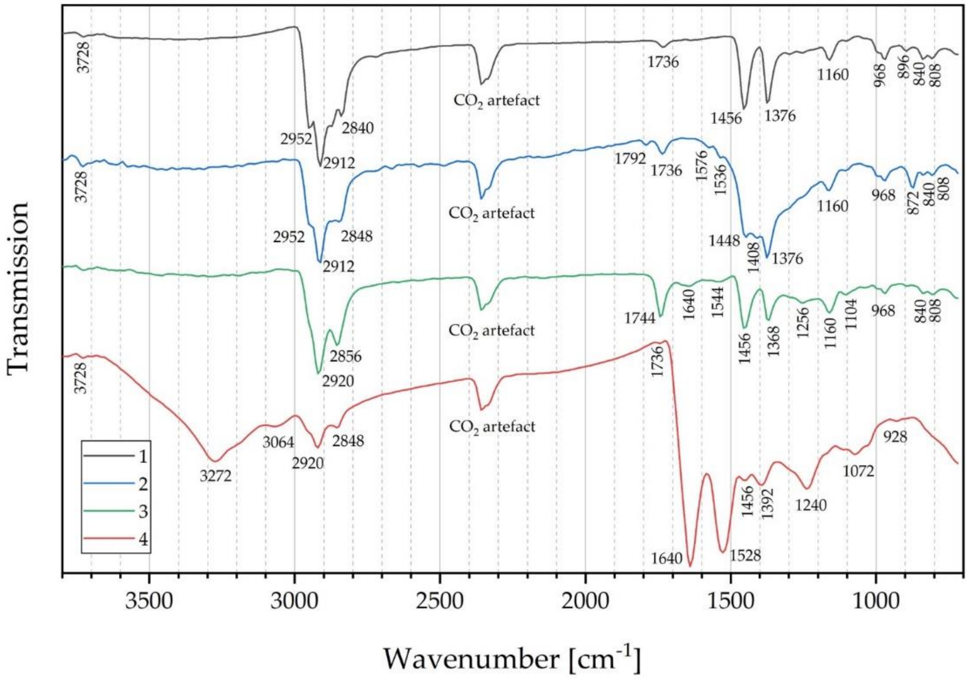

Figure S17. ATR-FTIR imaging of rPP-D sample 1. (a) ATR-FTIR image in false color representation recorded from a polished specimen cross-section exhibiting typical inclusions. This image is also shown in Fig. 4 (b). (b) Summary spectra obtained from the regions 1 to 4 shown in (a) including wavenumber assignments of relevant bands.

(a)

(b)

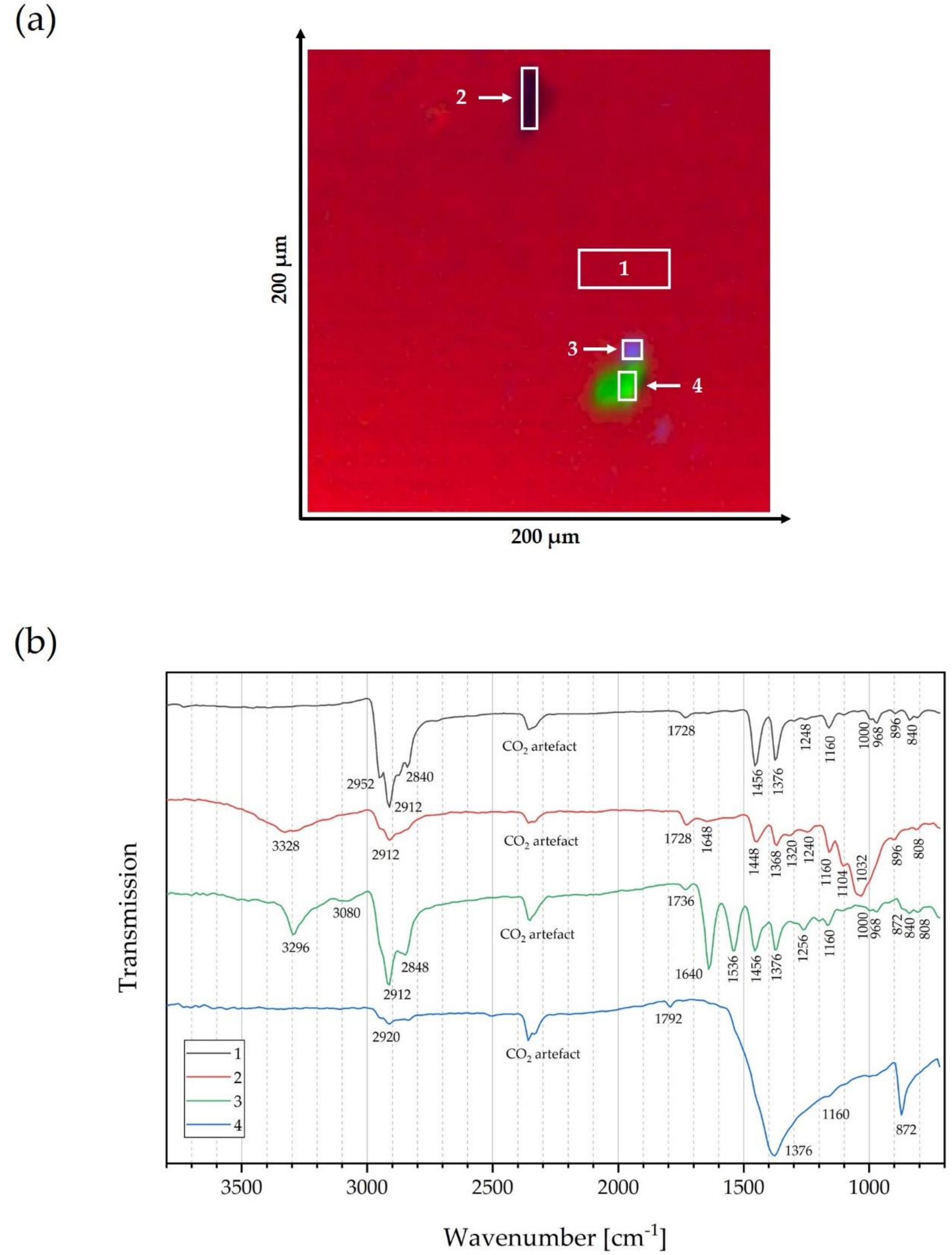

Figure S18. ATR-FTIR imaging of rPP-D sample 2. (a) ATR-FTIR image in false color representation recorded from a polished specimen cross-section exhibiting typical inclusions. (b) Summary spectra obtained from the regions 1 to 4 shown in (a) including wavenumber assignments of relevant bands.

(a)

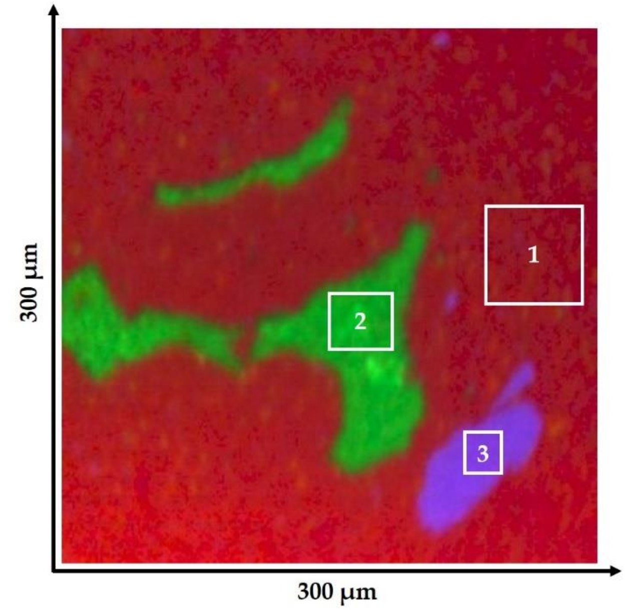

(b)

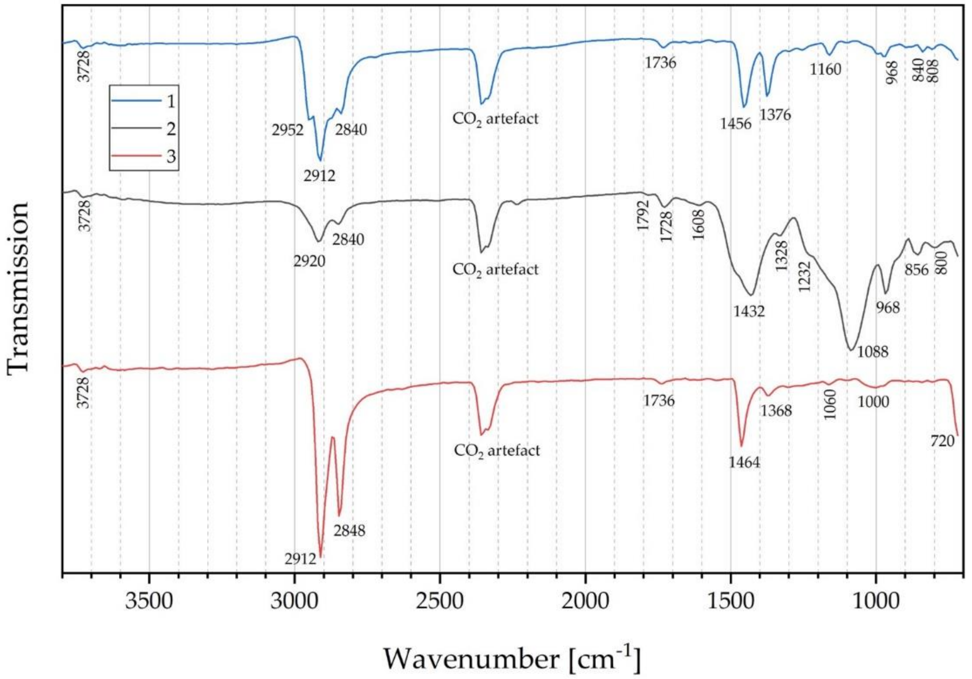

Figure S19. ATR-FTIR imaging of rPP-F sample 1. (a) ATR-FTIR image in false color representation recorded from a polished specimen cross-section exhibiting typical inclusions. This image is also shown in Fig. 4 (b). (b) Summary spectra obtained from the regions 1 to 3 shown in (a) including wavenumber assignments of relevant bands.

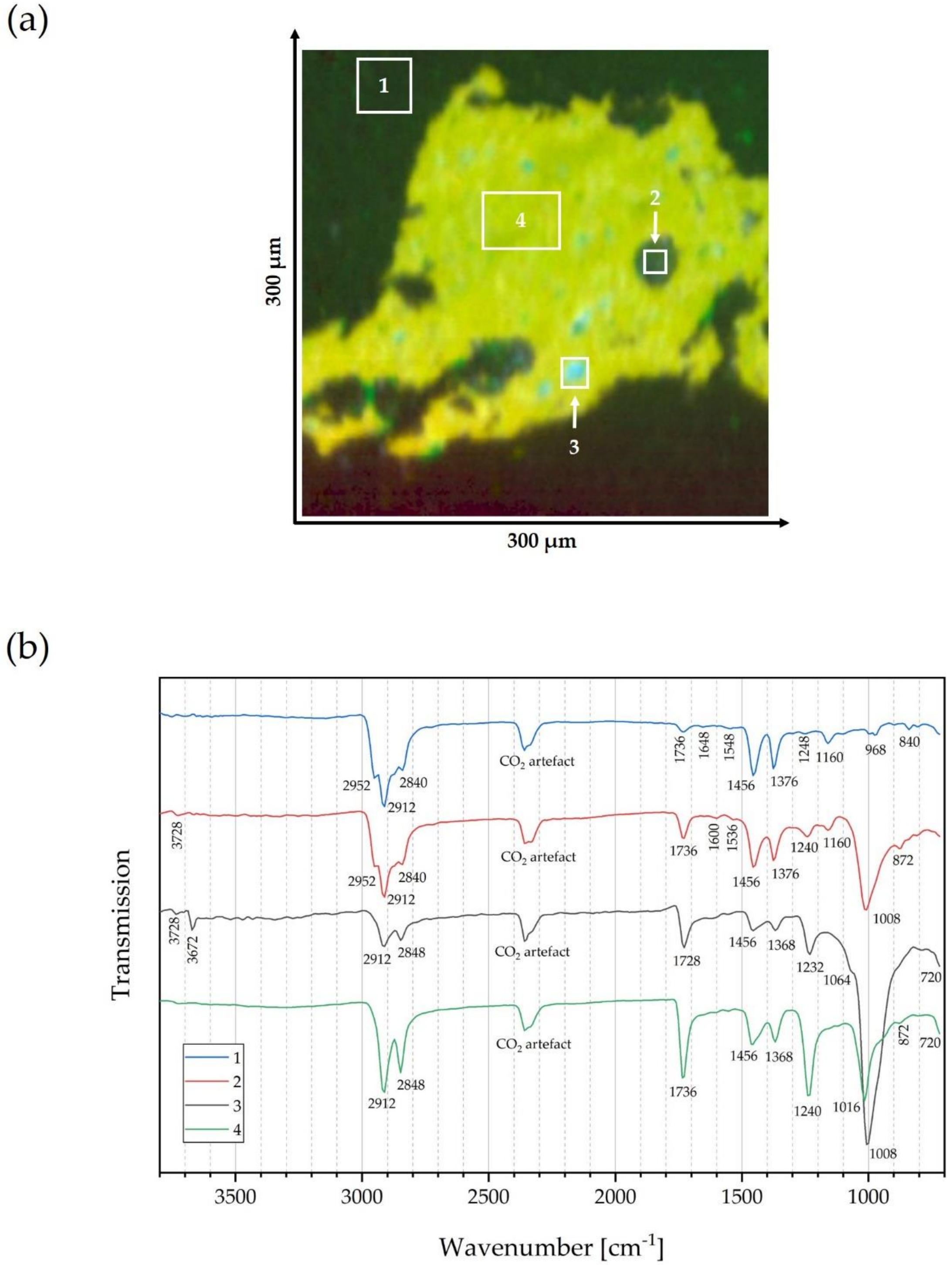

Figure S20. ATR-FTIR imaging of rPP-F sample 2. (a) ATR-FTIR image in false color representation recorded from a polished specimen cross-section exhibiting typical inclusions. (b) Summary spectra obtained from the regions 1 to 4 shown in (a) including wavenumber assignments of relevant bands.
