# Supplementary figures and images for: Characterization of Composition and Structure–Property Relationships of Commercial Post-Consumer Polyethylene and Polypropylene Recyclates
Source: Polymers (Basel). 2021 May 14;13(10):1574. doi: 10.3390/polym13101574 (PMC8156794; doi:10.3390/polym13101574)

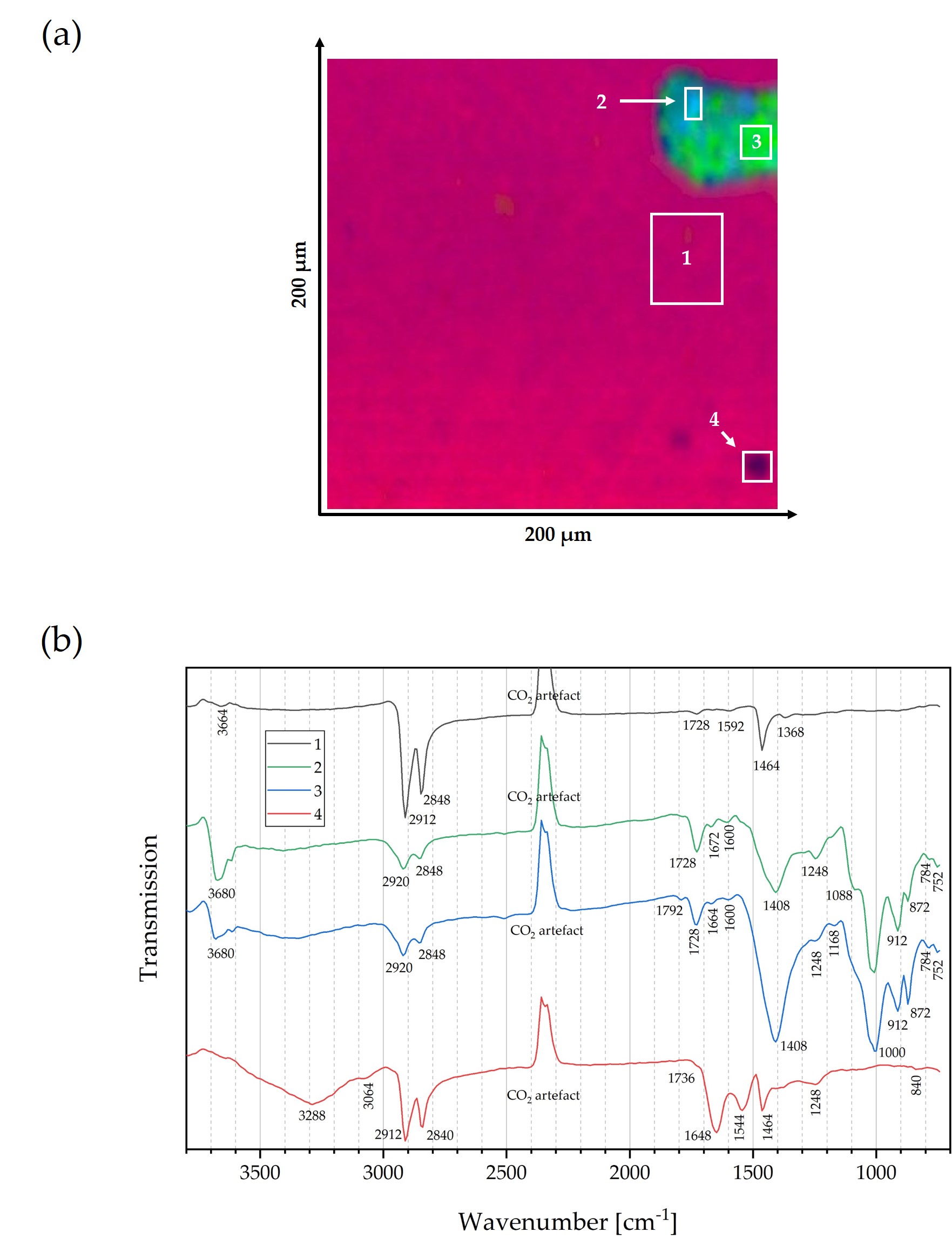

Supplement: Supplementary file 1 [file polymers-13-01574-s001.zip › S1 rPE-A sample 1.jpg]

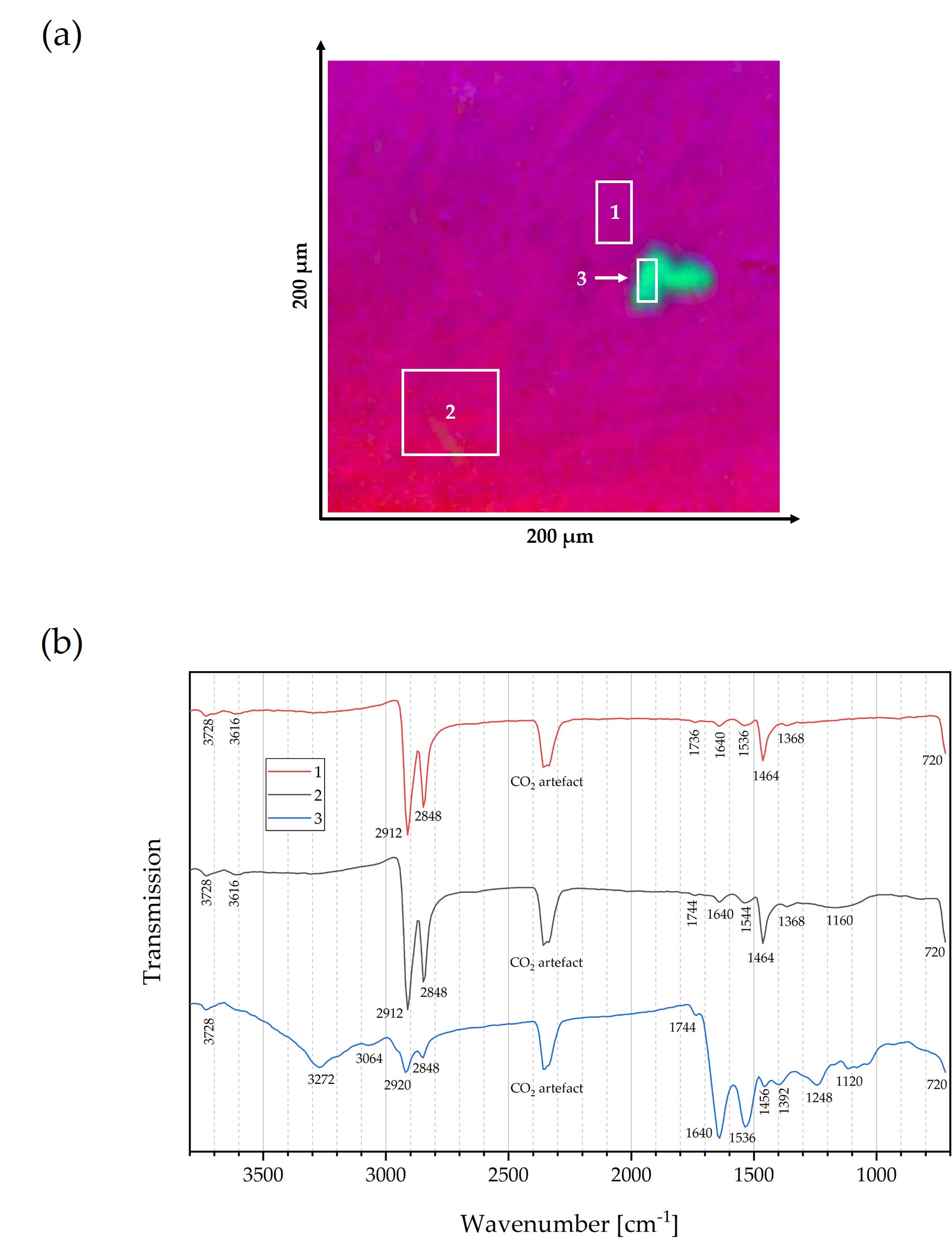

Supplement: Supplementary file 1 [file polymers-13-01574-s001.zip › S10 rPE-E2 sample 2.jpg]

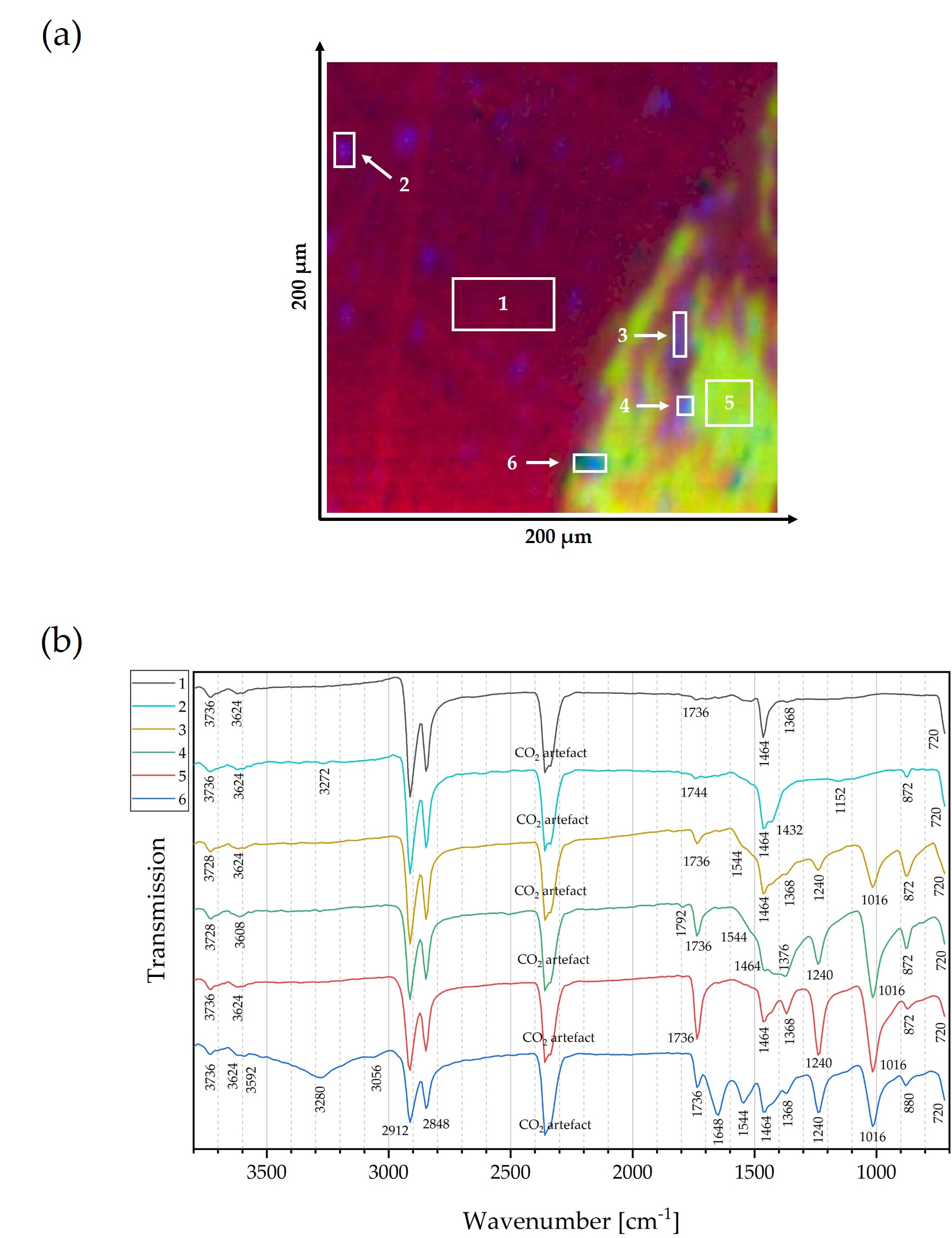

Supplement: Supplementary file 1 [file polymers-13-01574-s001.zip › S11 rPE-F sample 1.jpg]

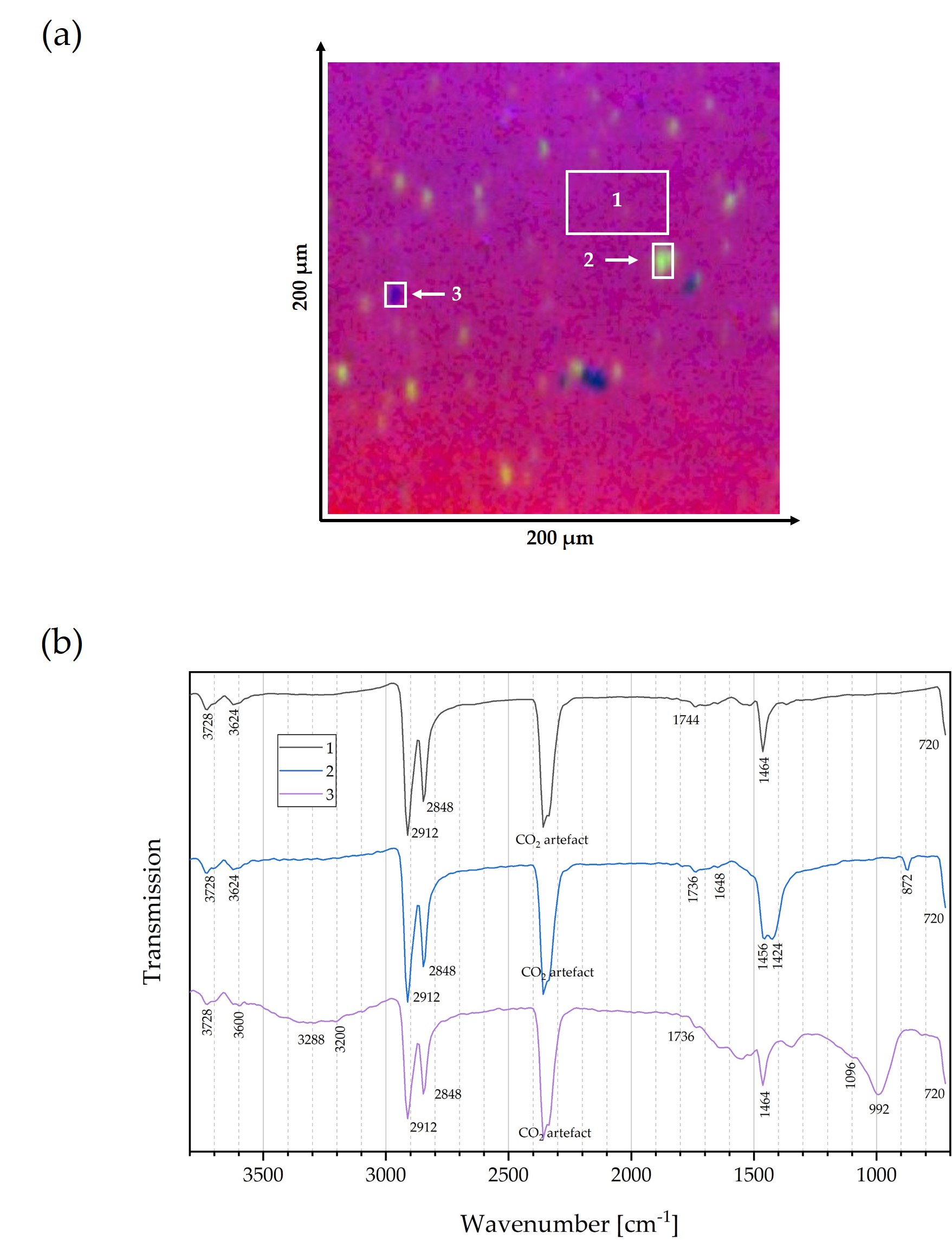

Supplement: Supplementary file 1 [file polymers-13-01574-s001.zip › S12 rPE-F sample 2.jpg]

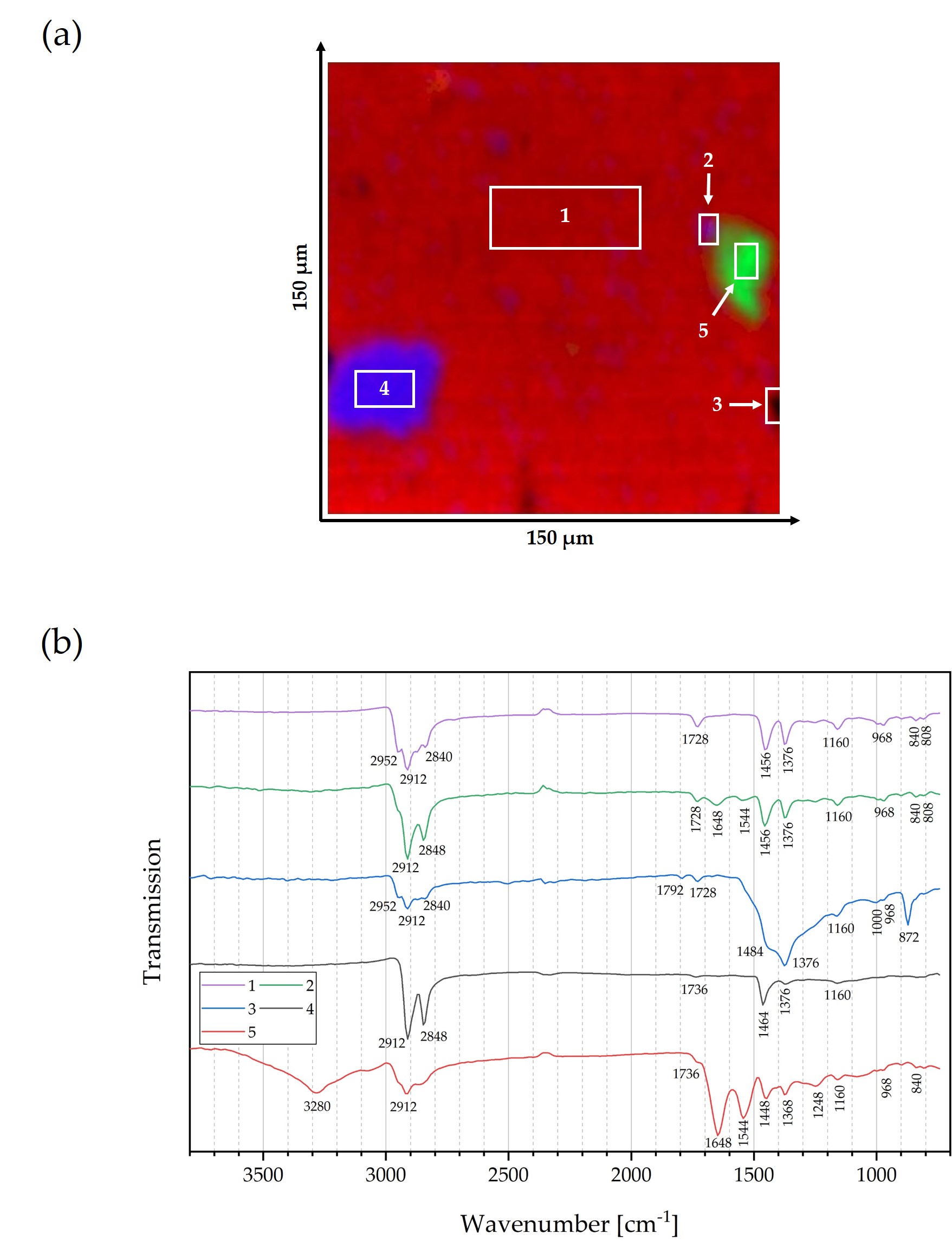

Supplement: Supplementary file 1 [file polymers-13-01574-s001.zip › S13 rPP-A sample 1.jpg]

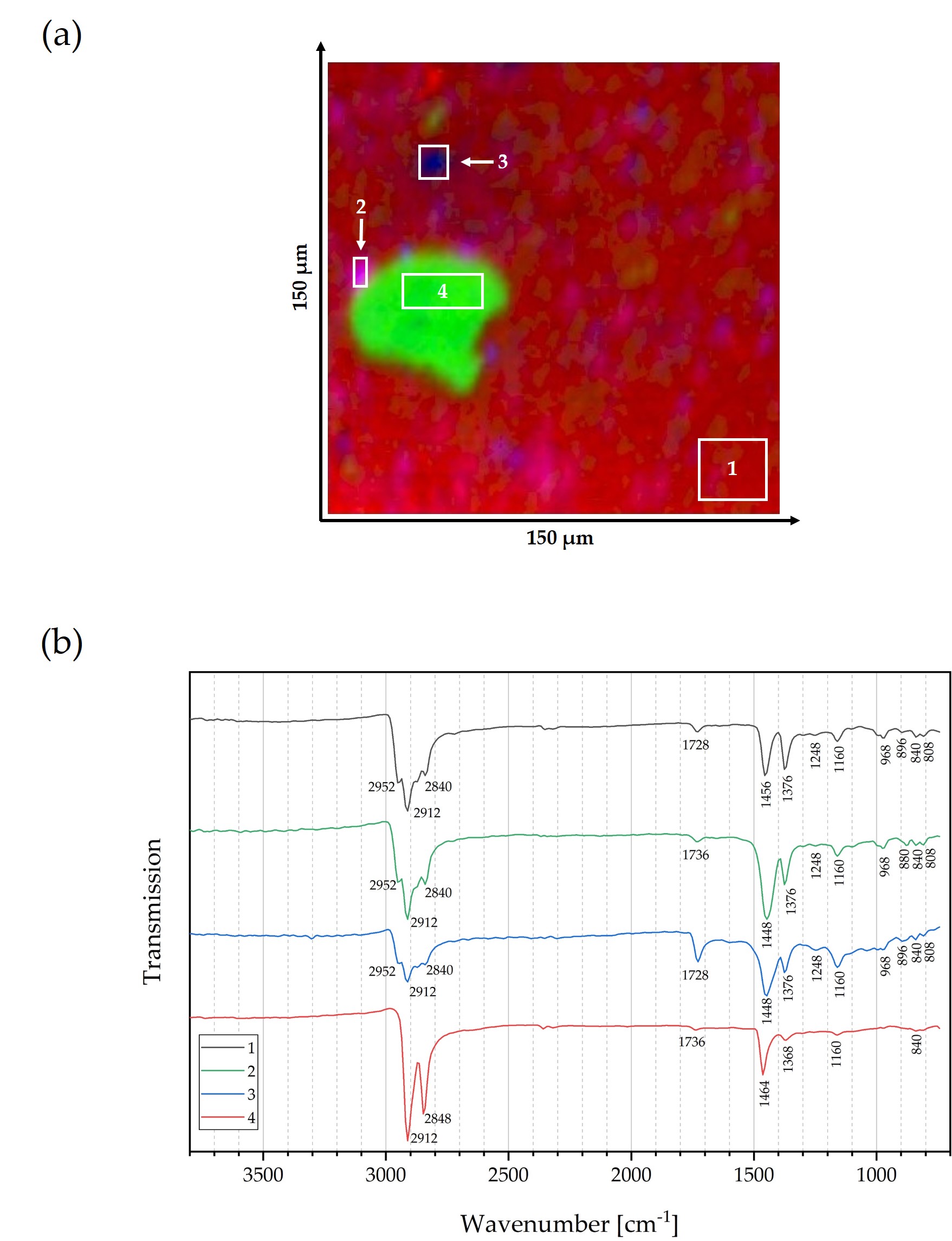

Supplement: Supplementary file 1 [file polymers-13-01574-s001.zip › S14 rPP-A sample 2.jpg]

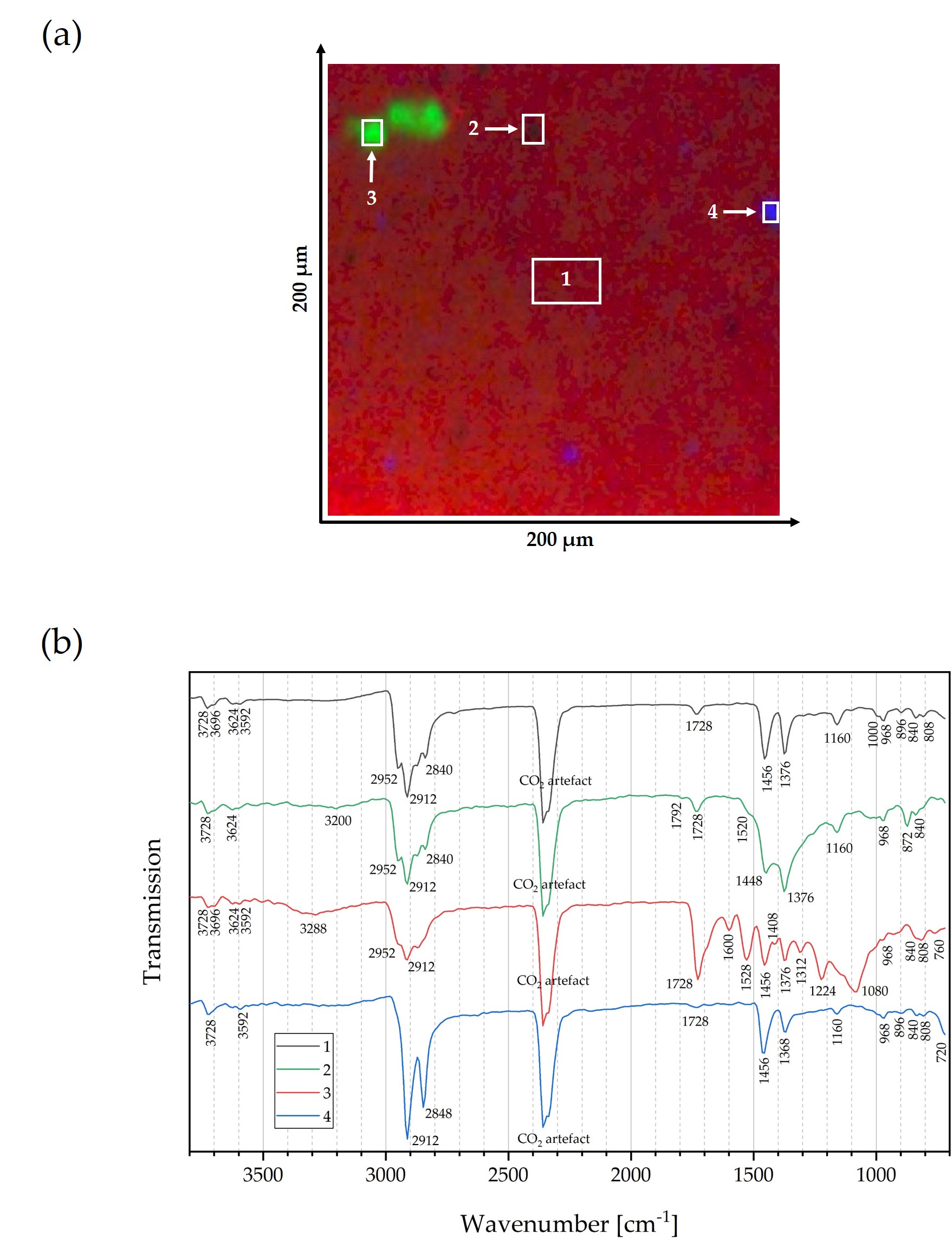

Supplement: Supplementary file 1 [file polymers-13-01574-s001.zip › S15 rPP-C sample 1.jpg]

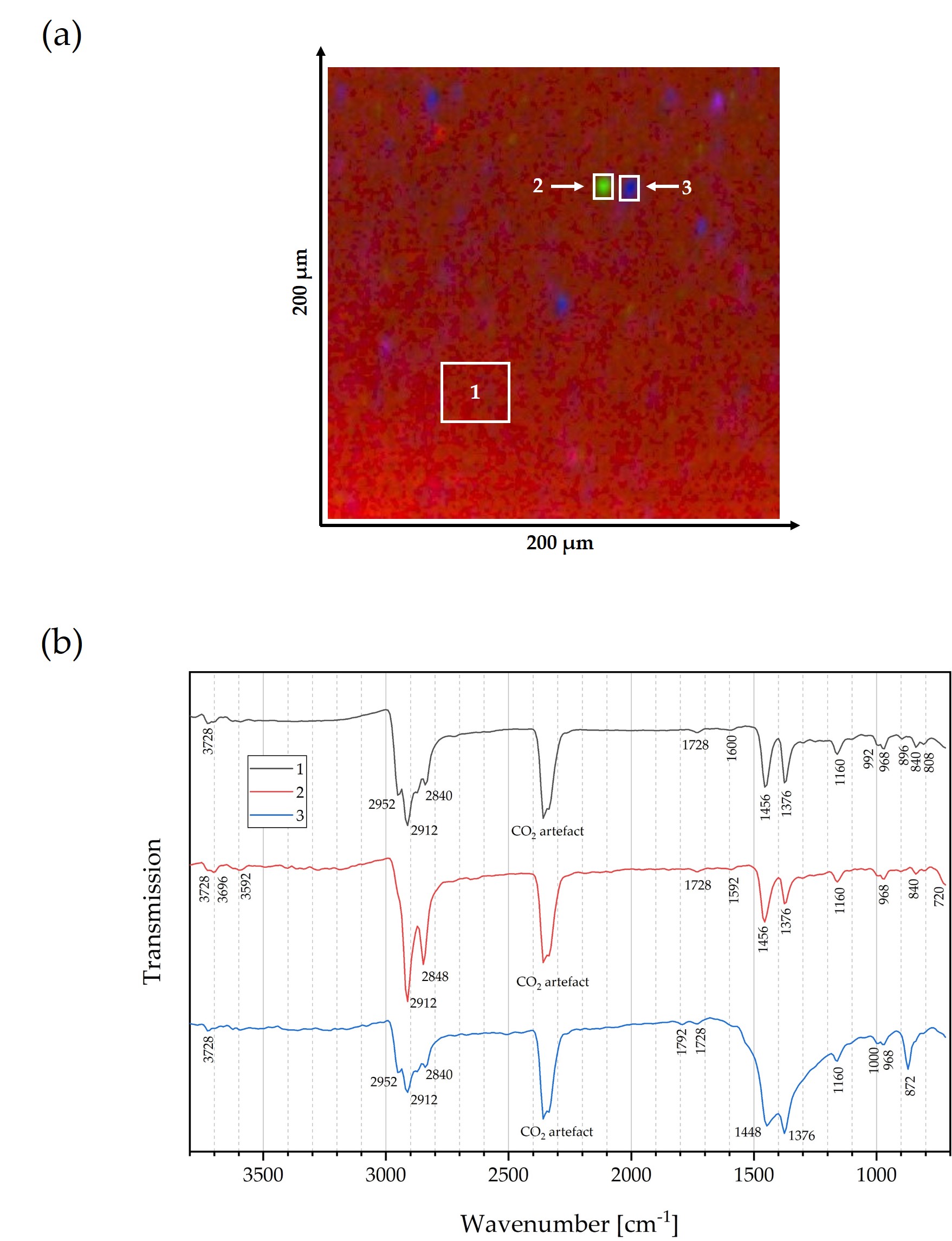

Supplement: Supplementary file 1 [file polymers-13-01574-s001.zip › S16 rPP-C sample 2.jpg]

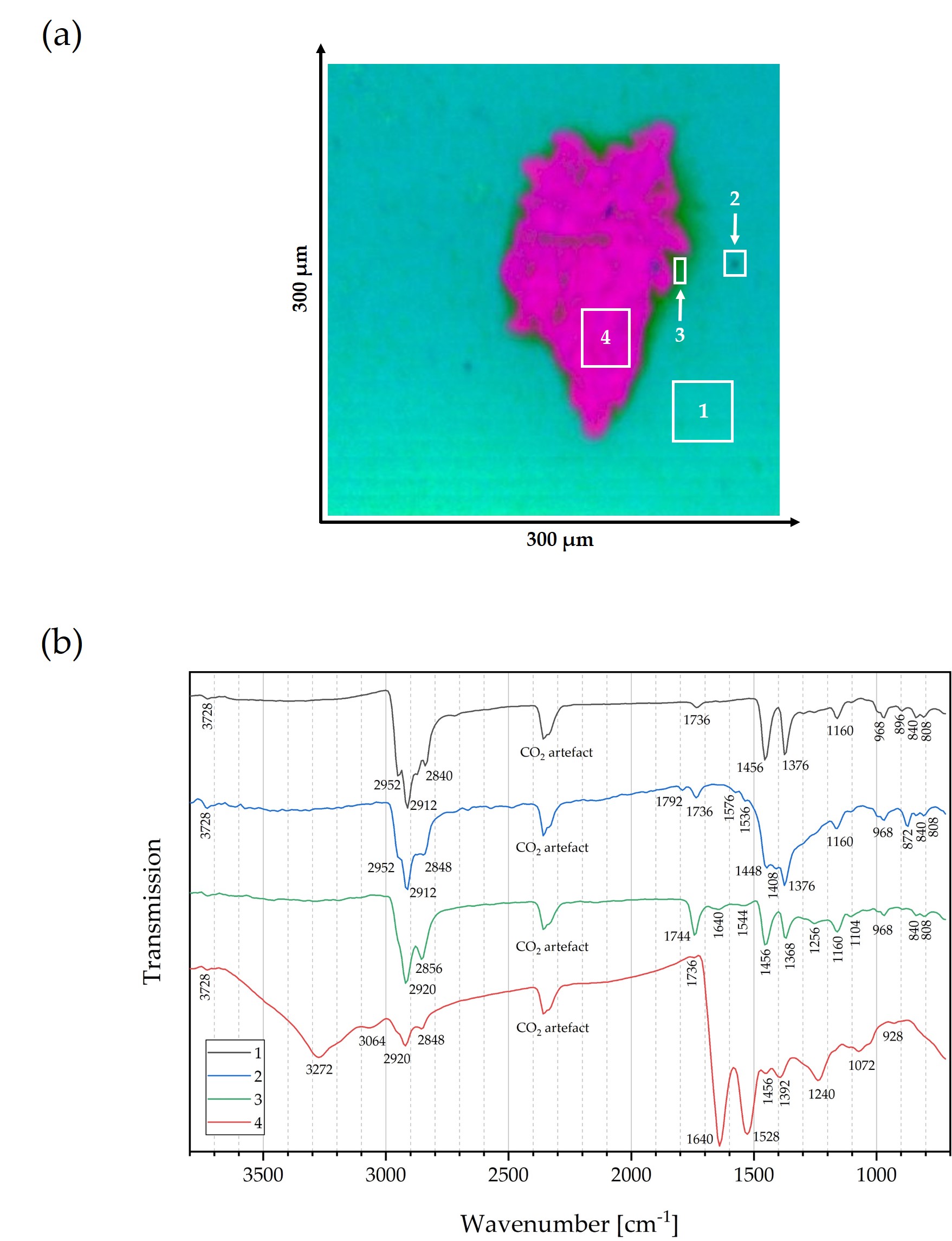

Supplement: Supplementary file 1 [file polymers-13-01574-s001.zip › S17 rPP-D sample 1.jpg]

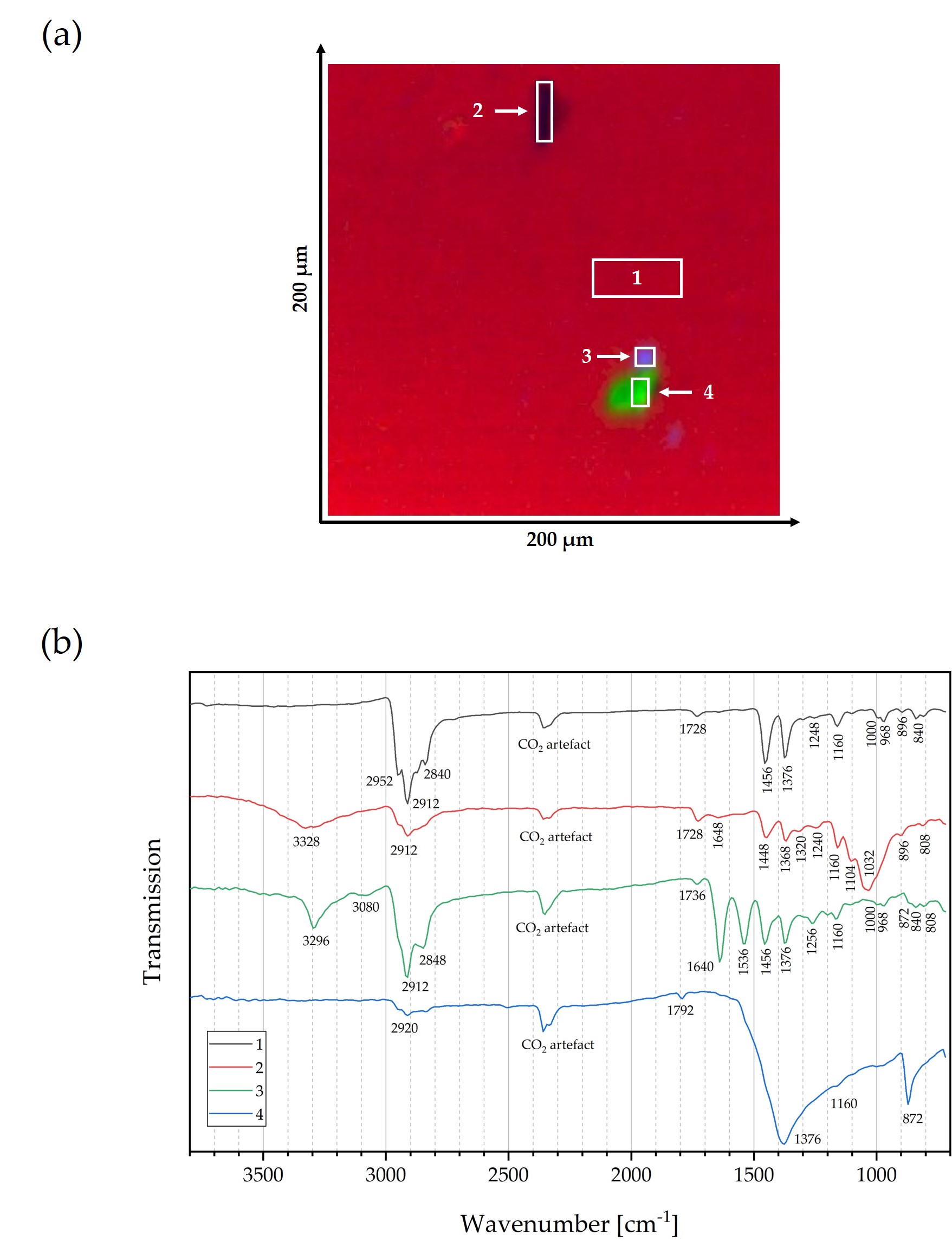

Supplement: Supplementary file 1 [file polymers-13-01574-s001.zip › S18 rPP-D sample 2.jpg]

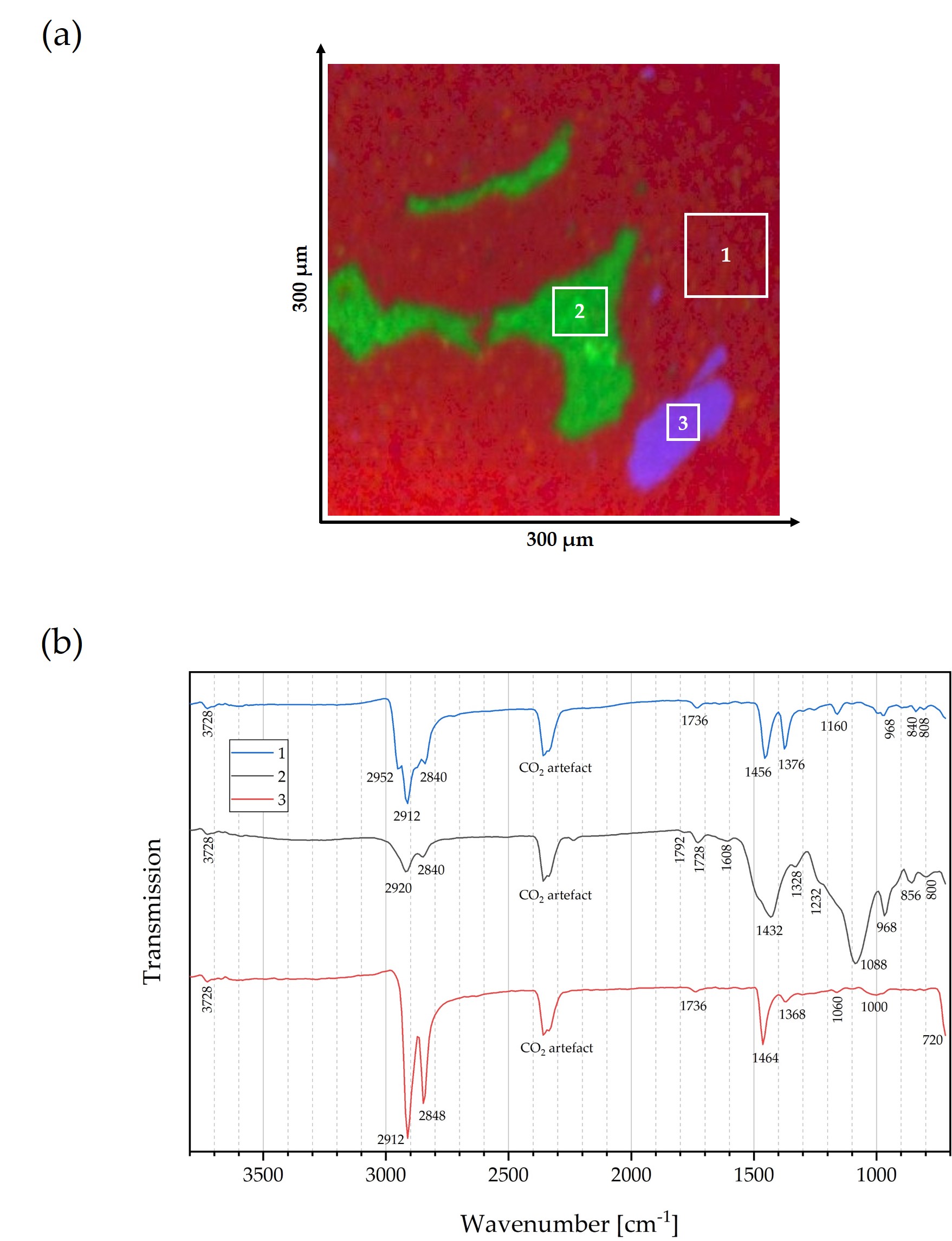

Supplement: Supplementary file 1 [file polymers-13-01574-s001.zip › S19 rPP-F sample 1.jpg]

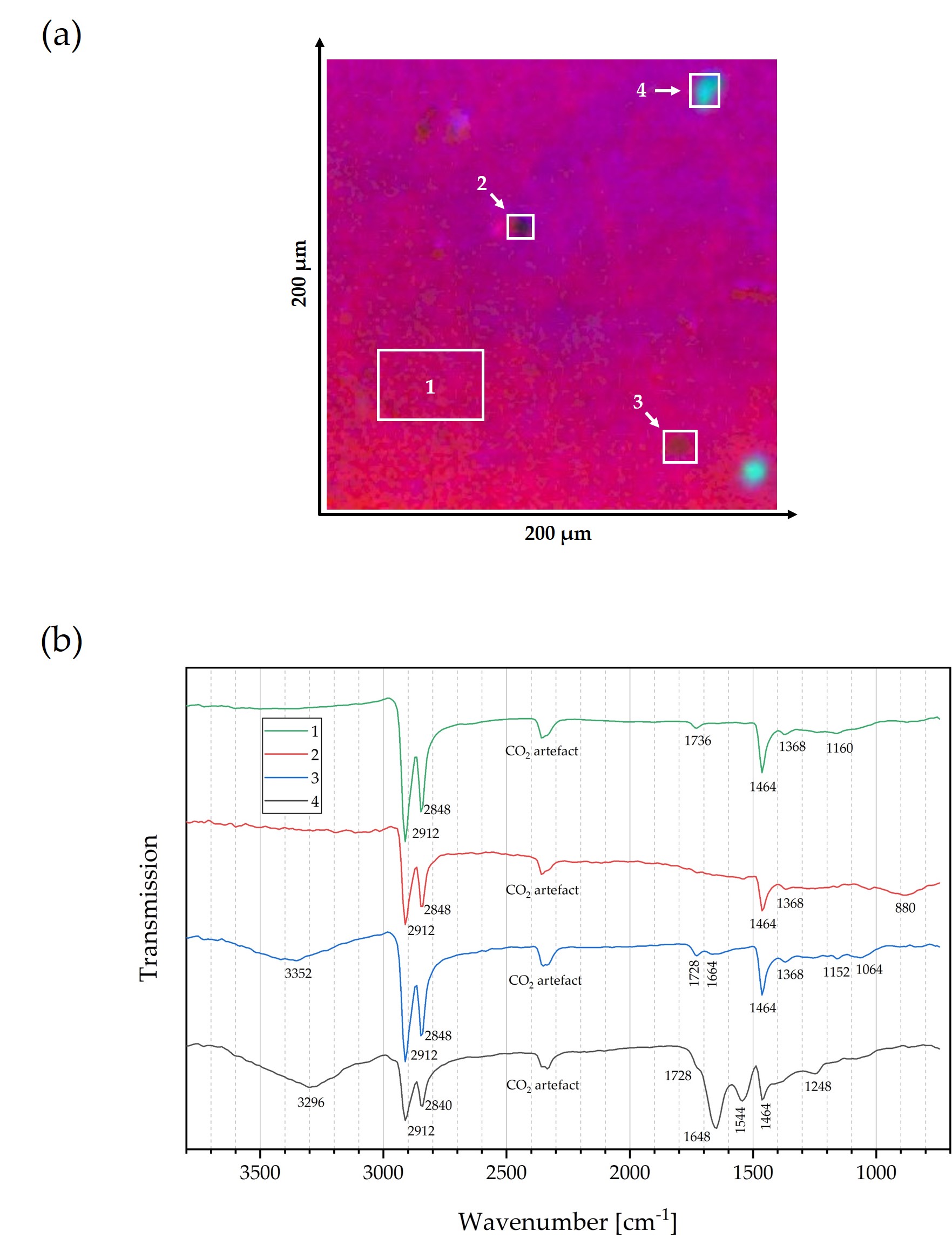

Supplement: Supplementary file 1 [file polymers-13-01574-s001.zip › S2 rPE-A sample 2.jpg]

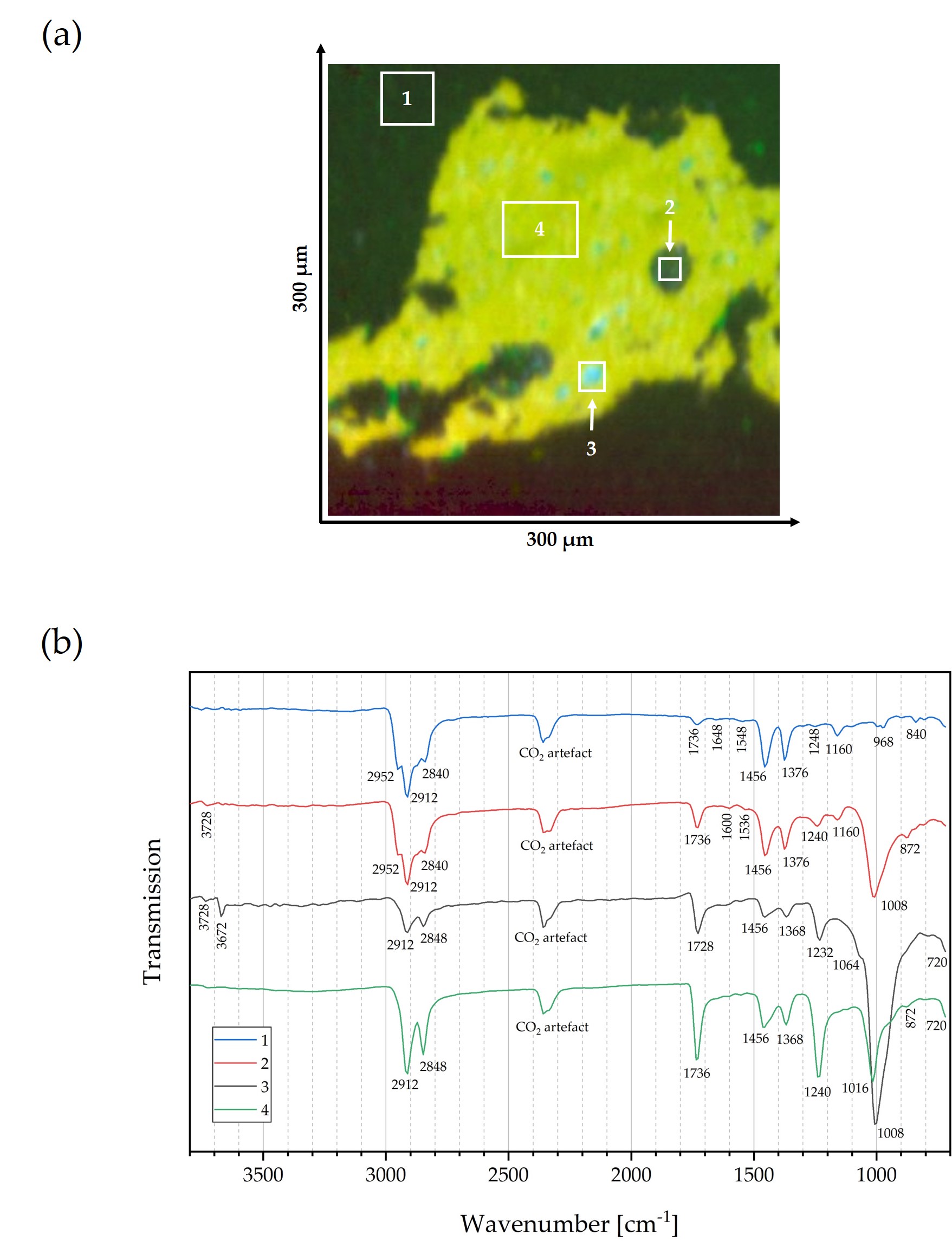

Supplement: Supplementary file 1 [file polymers-13-01574-s001.zip › S20 rPP-F sample 2.jpg]

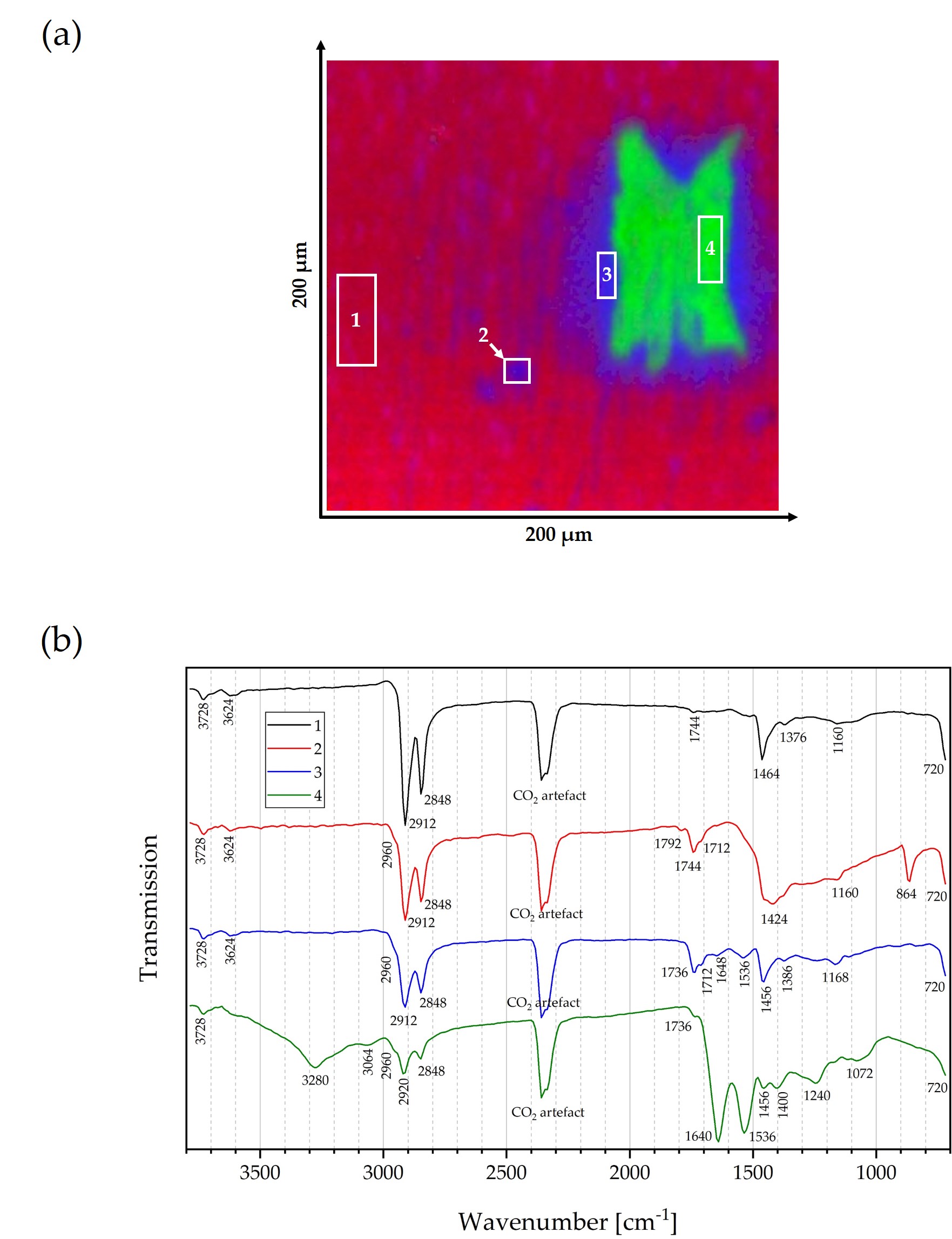

Supplement: Supplementary file 1 [file polymers-13-01574-s001.zip › S3 rPE-C sample 1.jpg]

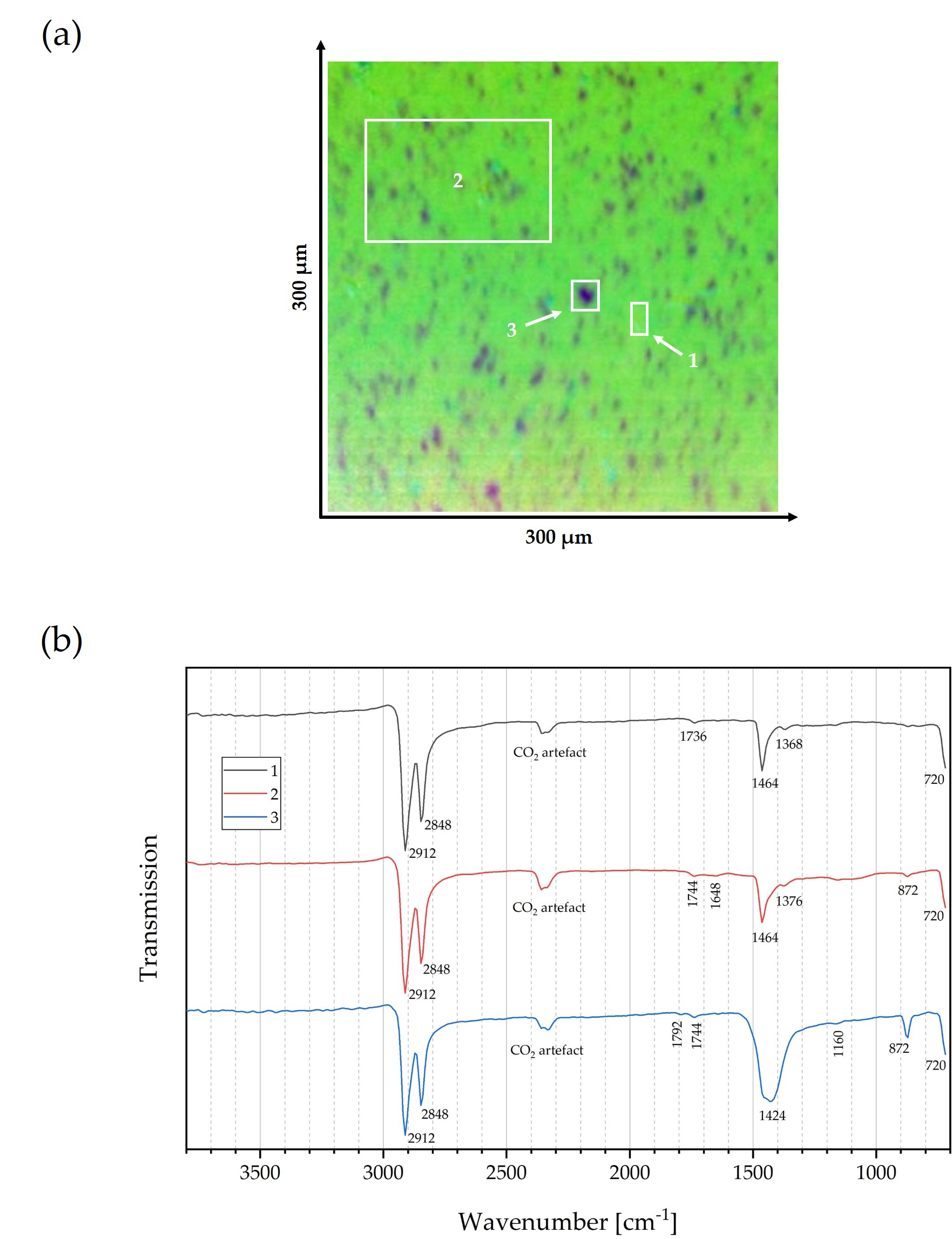

Supplement: Supplementary file 1 [file polymers-13-01574-s001.zip › S4 rPE-C sample 2.jpg]

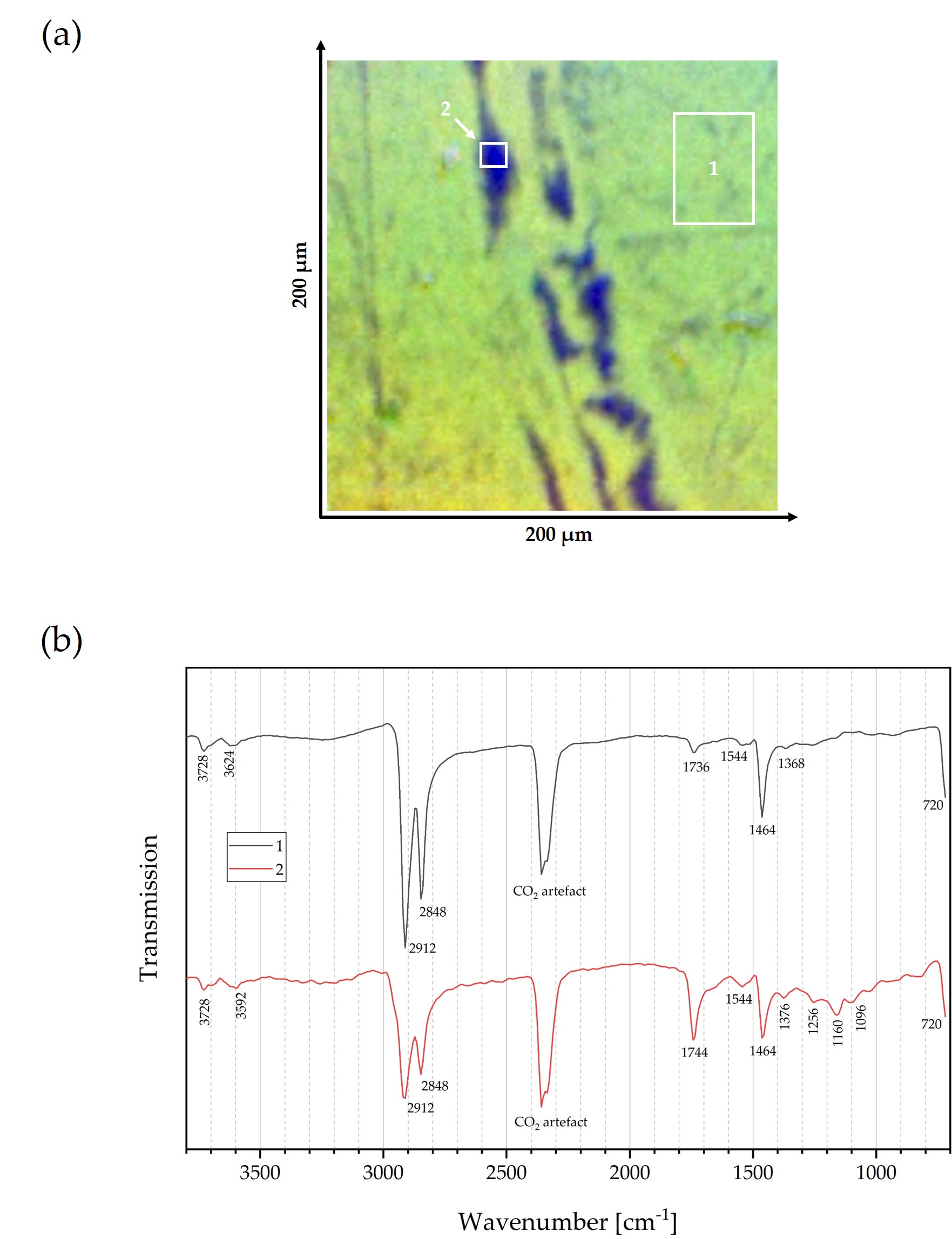

Supplement: Supplementary file 1 [file polymers-13-01574-s001.zip › S5 rPE-D sample 1.jpg]

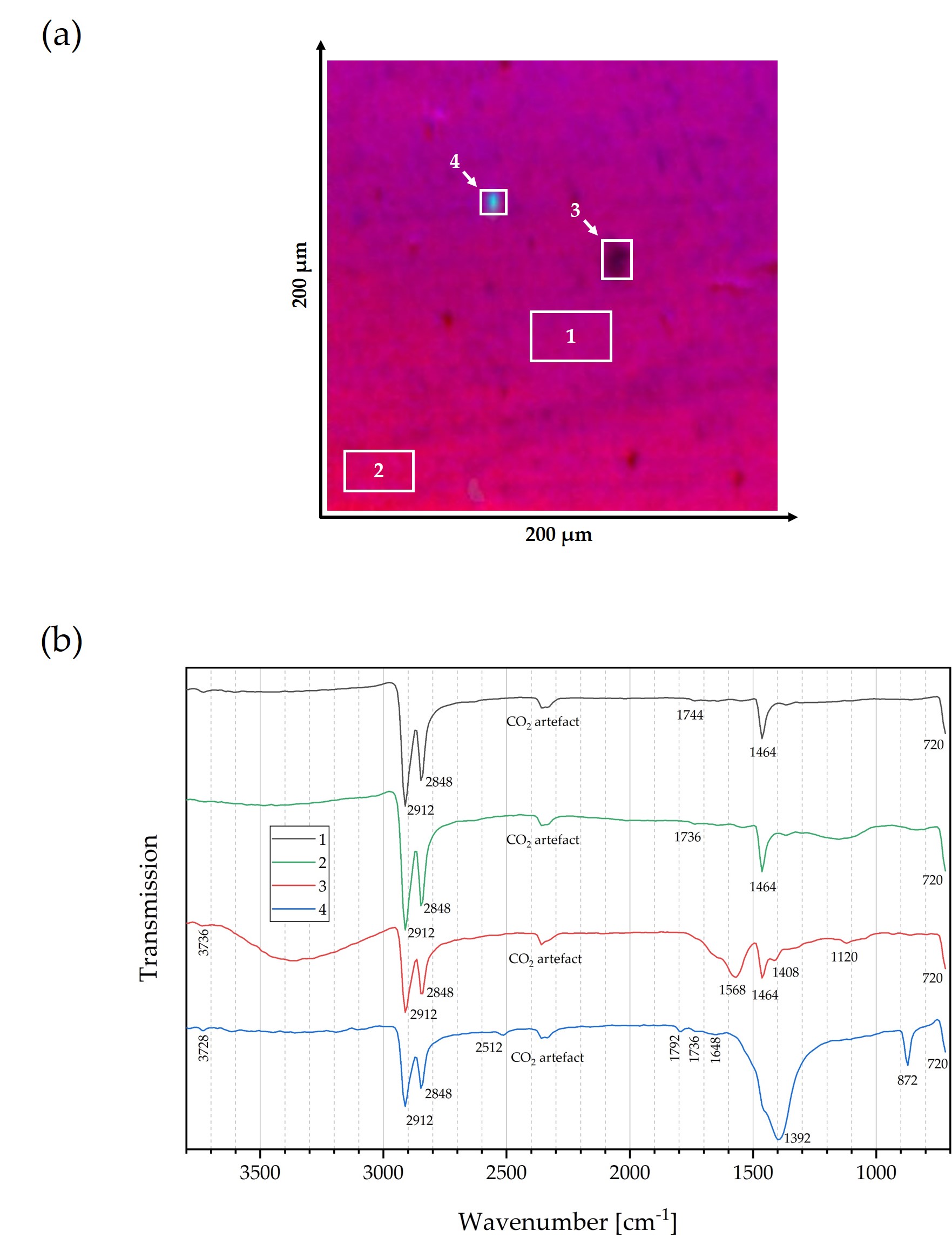

Supplement: Supplementary file 1 [file polymers-13-01574-s001.zip › S6 rPE-D sample 2.jpg]

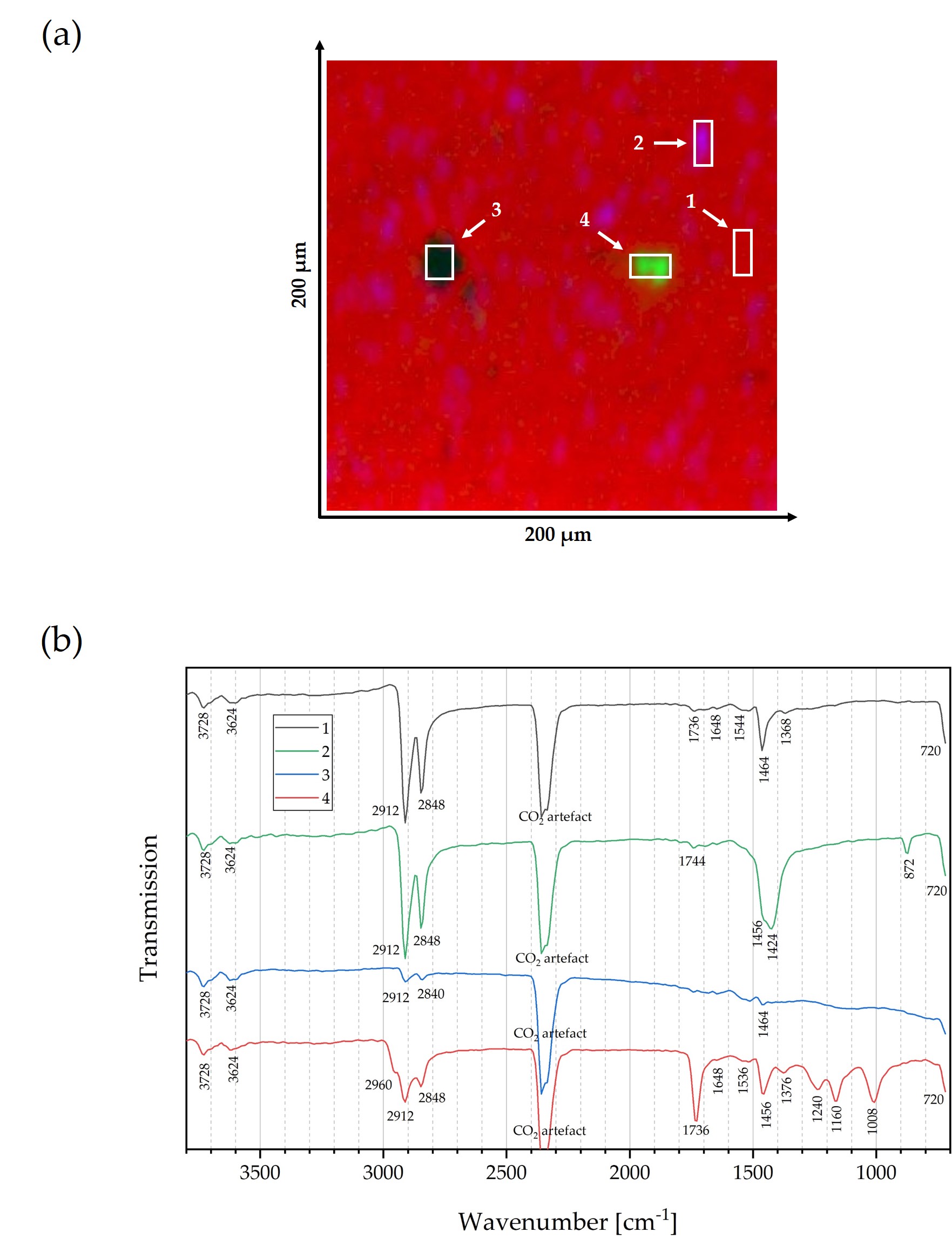

Supplement: Supplementary file 1 [file polymers-13-01574-s001.zip › S7 rPE-E1 sample 1.jpg]

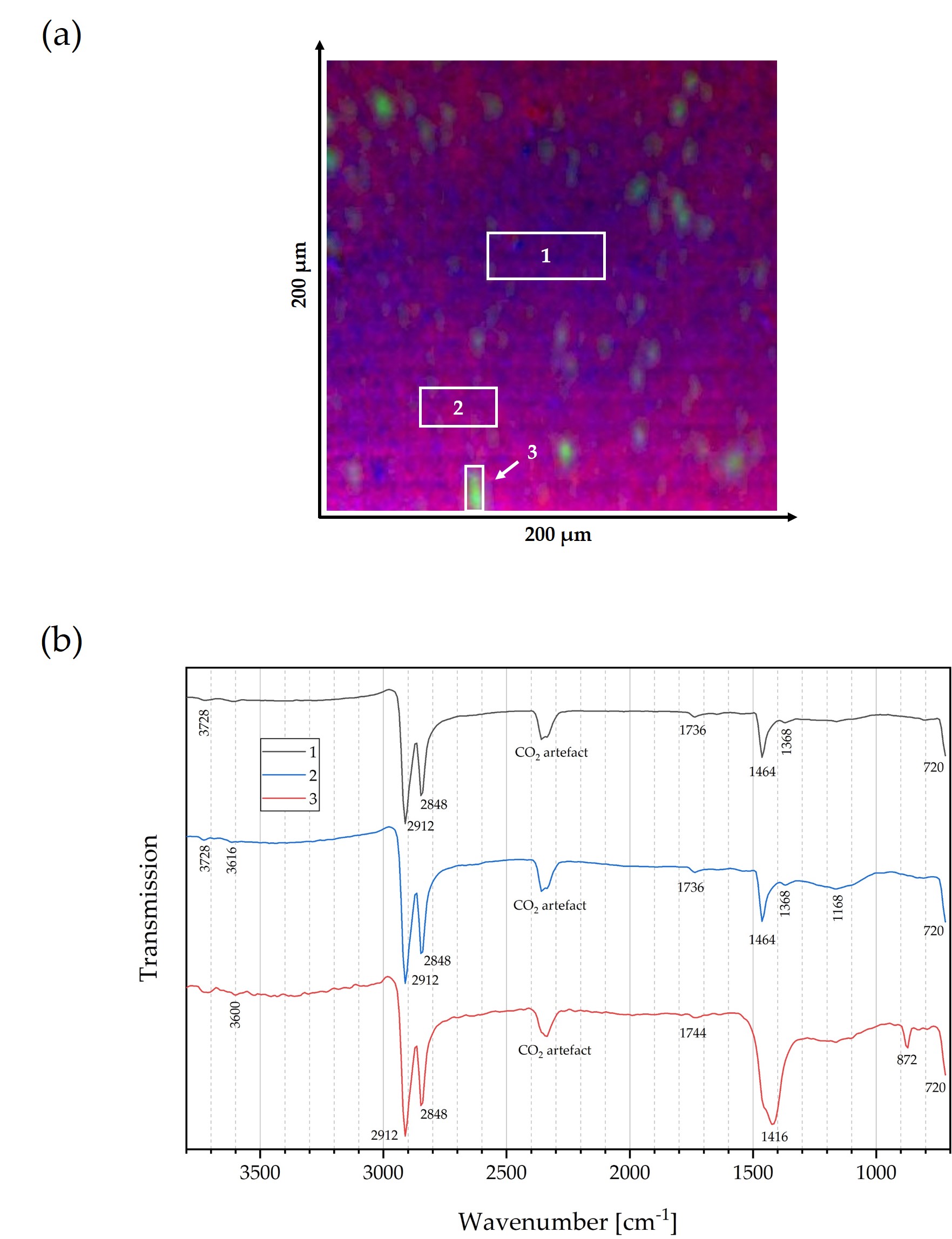

Supplement: Supplementary file 1 [file polymers-13-01574-s001.zip › S8 rPE-E1 sample 2.jpg]

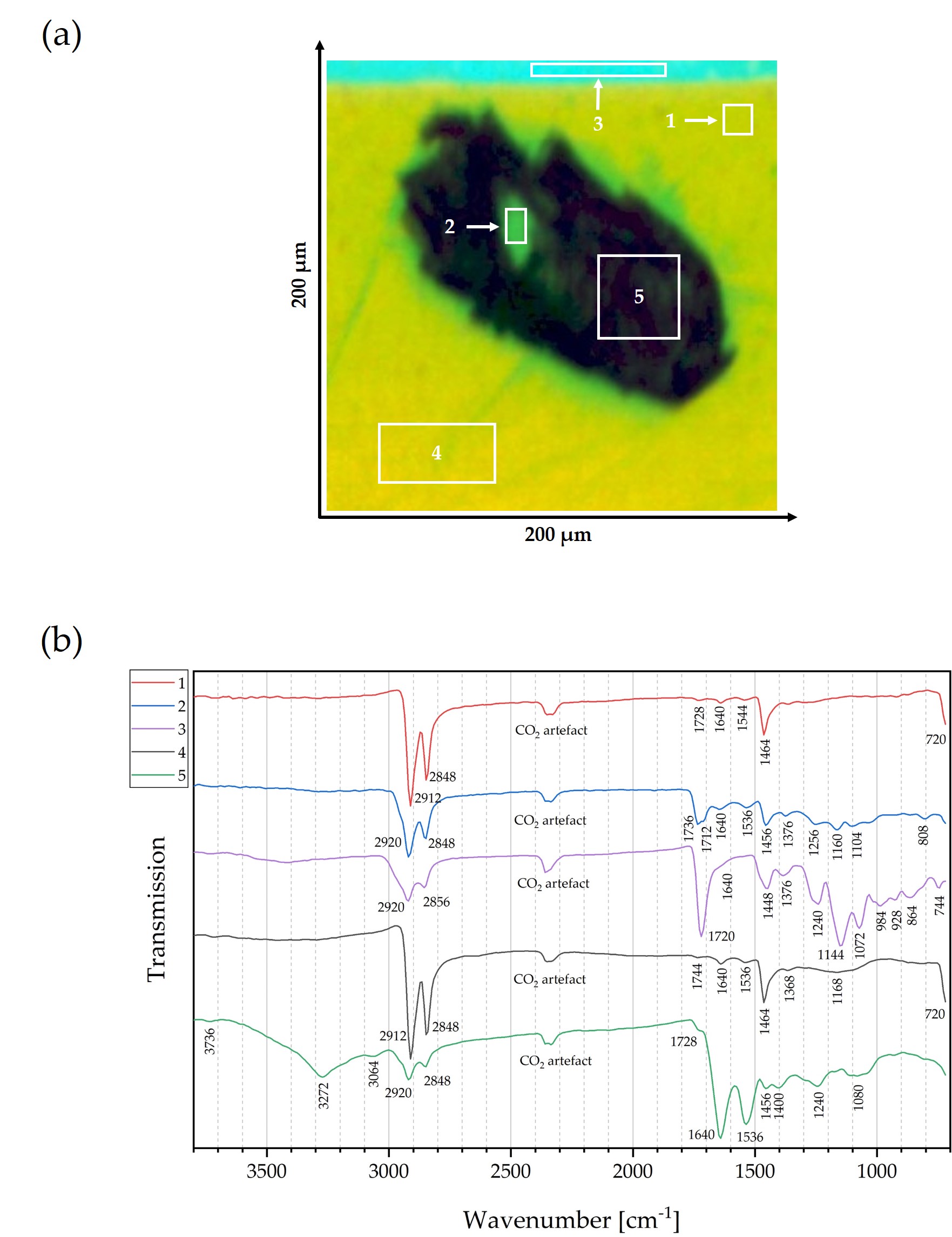

Supplement: Supplementary file 1 [file polymers-13-01574-s001.zip › S9 rPE-E2 sample 1.jpg]
